# Transient Structural Dynamics of Glycogen Phosphorylase from Nonequilibrium Hydrogen/Deuterium-Exchange Mass Spectrometry

**DOI:** 10.1021/jacs.3c08934

**Published:** 2023-12-29

**Authors:** Monika Kish, Dylan P. Ivory, Jonathan J. Phillips

**Affiliations:** † Living Systems Institute, Department of Biosciences, University of Exeter, Stocker Road, Exeter EX4 4QD, U.K.; ‡ Alan Turing Institute, British Library, London NW1 2DB, U.K.

## Abstract

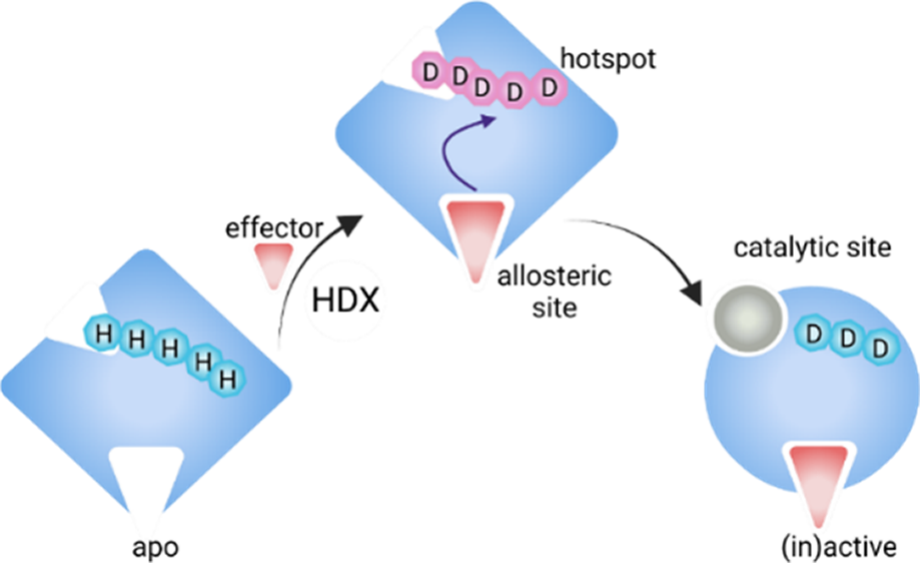

It remains a major challenge to ascertain the specific structurally dynamic changes that underpin protein functional switching. There is a growing need in molecular biology and drug discovery to complement structural models with the ability to determine the dynamic structural changes that occur as these proteins are regulated and function. The archetypal allosteric enzyme glycogen phosphorylase is a clinical target of great interest to treat type II diabetes and metastatic cancers. Here, we developed a time-resolved nonequilibrium millisecond hydrogen/deuterium-exchange mass spectrometry (HDX-MS) approach capable of precisely locating dynamic structural changes during allosteric activation and inhibition of glycogen phosphorylase. We resolved obligate transient changes in the localized structure that are absent when directly comparing active/inactive states of the enzyme and show that they are common to allosteric activation by AMP and inhibition by caffeine, operating at different sites. This indicates that opposing allosteric regulation by inhibitor and activator ligands is mediated by pathways that intersect with a common structurally dynamic motif. This mass spectrometry approach uniquely stands to discover local transient structural dynamics and could be used broadly to identify features that influence the structural transitions of proteins.

## Introduction

Allostery refers to the transfer of signal between two sites of a protein, resulting in a change in the catalytic activity of the protein. These sophisticated structural transitions are fundamental to receptor transduction,^1^ cell signaling,^2^ and metabolic regulation.^3^ Despite extensive studies since its inception,^4−6^ very little is known about precisely how these signals are transmitted long distances through a protein molecule. This is in large part due to the lack of biophysical approaches to measure these signals at high resolution in both time and space.

Recently, important advances have been made toward this goal in single-molecule Förster resonance energy transfer (FRET),^7−10^ nuclear magnetic resonance,^11^ time-resolved electron cryomicroscopy (cryo-EM),^12^ time-resolved crystallography,^13^ molecular dynamics simulations,^14,15^ and double electron–electron resonance.^16,17^ Perhaps the most direct evidence for an intramolecular mechanism comes from infrared laser absorbance and emission experiments of conjugated peptides, which strongly argues that the vibrational energy transfer (VET) is mediated by hydrogen bonds.^18^ Collectively, these studies begin to reveal nonequilibrium protein structural dynamics at high structural and temporal resolution, as first described by Monod and colleagues in 1965.^19^ Though impressive, these methods still require considerable adaptation per sample and are not yet broadly applicable to cases of ligand-induced allostery in proteins.

Glycogen phosphorylase (GlyP) is the archetypal allosteric enzyme whose regulation is tightly coupled to solid tumor metastasis,^20^ type II diabetes,^21^ and adaptive immunity (early memory CD8+ T-cell recall response).^22^ GlyP catalyzes the first executed energetic step of carbohydrate metabolism from glycogen stores and, as such, is one of the most highly regulated enzymes known, with at least six allosteric sites for natural ligands and drugs. GlyP has been intensively studied since the 1930s,^23^ thus there is abundance of structural and kinetic data on this protein system,^24−33^ with detailed studies on the activation mechanism^34^ and stabilization of R/T-states (active/inactive states) by allosteric ligands^35^ and phosphorylation.^36^ GlyP is, therefore, an ideal system to challenge our ability to discern dynamic structural mechanisms of allosteric regulation as it is alternately activated by adenosine monophosphate (AMP) at the “nucleotide site” and inhibited by caffeine (CFF) at the “inhibitor site”,^37,38^ while also being constitutively active by Ser 14 phosphorylation.^19^ Binding of AMP at the allosteric effector site brings similar changes to phosphorylation but is believed to be through somewhat different mechanisms.^39^ How a single protein can support allosteric activation and inhibition by multiple intersecting structural pathways has not yet been elucidated.

Hydrogen/deuterium-exchange mass spectrometry (HDX-MS) is a sensitive technique for quantifying backbone amide hydrogen exchange when exposed to solvent, either at a molecular surface or due to local unfolding events. The amide HDX directly relates to local structural dynamics and now can be routinely determined in large protein systems,^40,41^ with millisecond time resolution^42−44^ and at or near single amino acid structural resolution.^45−48^

Here, we developed a millisecond nonequilibrium hydrogen/deuterium-exchange mass spectrometry (neHDX-MS) approach to identify specific dynamic changes in a large enzyme during allosteric regulation. The difference in deuterium labeling before, during, and after ligand binding provides a structurally resolved measurement of dynamic reconfiguration. Equilibrium experiments reflect local minima of R/T-states, while nonequilibrium data reveal transition state ensembles between local minima on the energy landscape.^49^ We clearly identified amino acids in a coherent motif that participate in transient conformational dynamics, and we propose a structural pathway for those changes upon allosteric ligand binding. This mass spectrometry approach stands to uniquely discover local structural dynamics and can be used broadly to identify such features in many different proteins.

## Results

### Nonequilibrium Allosteric Activation/Inhibition of GlyP

**Figure 1:**
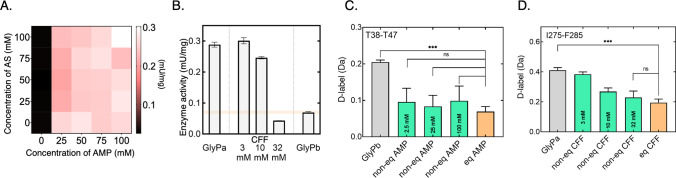
Allosteric activation of glycogen phosphorylase b and inhibition of glycogen phosphorylase a. (A) Heat map of measured activity for combinations of AMP/AS concentrations in range 0–100 mM. Inactive GlyPb (black); highest activity achieved (white). Activity was calculated by a standard curve for *n* = 2. (B) Catalytic activity of GlyPa supplemented with caffeine. Shaded region—GlyPb, 95% confidence intervals. (C) AMP binding saturation during the dead time of the HDX labeling (50 ms). Nonequilibrium addition of 25 mM AMP/25 mM AS is optimal to fully saturate the nucleotide site. (D) Caffeine binding saturation during the dead time of the HDX labeling (50 ms). Nonequilibrium addition of 32 mM caffeine is optimal to fully saturate the inhibitor site.

To observe the kinetically populated transition state of GlyP activation and inhibition, we first established the chemical conditions that fully activate or inhibit the enzyme. The catalytic activity for the fully active form phosphorylated at Ser14 (GlyPa) was used as a positive control reference compared with unphosphorylated inactive enzyme (GlyPb) in a glycogen hydrolysis colorimetric assay (Figures 1A and S2A,D,E). The maximal GlyPb allosteric activation by AMP was then established.^25,39^ This was achieved with AMP ≥ 25 mM, the lowest concentration of AMP tested (range 25–100 mM), given no significant difference between the activity of GlyPb + AMP and that of GlyPa (Figure S2D). We also sought to verify the requirement of ammonium sulfate (AS) for full activation, known to mimic the presence of phosphate by binding GlyP at the AMP effector site, catalytic site, and Ser14 phosphorylation site.^19^ There are conflicting reports on the requirement of AS for full activation of GlyPb cooperatively with AMP. While GlyPb affinity for AMP is reported to increase 50-fold and activity to increase by 50% with sulfate,^24,50−52^ here we found activation by sulfate alone was not achieved (15% of maximum), nor was a cooperative effect observed on activity (Figures 1A and S2). However, HDX-MS identified cooperativity in GlyPb structural activation by AMP/AS that is not readily observed in the activity assay (Figure S2D,C).^24,39,53^

To understand these findings, further equilibrium HDX-MS experiments were performed with various concentrations of AMP/AS. These experiments showed cooperativity between AMP and AS, specifically at the nucleotide site, 380 loop, and tower helix (Figure S2C).

One-way ANOVA tests proved that higher concentrations of AMP or AS were not significant for either catalytic activity or structural perturbation. Therefore, the lowest concentrations tested of 25 mM AMP and 25 mM AS were considered to fully activate GlyPb catalytically and structurally, and these conditions were used for the experiments to study nonequilibrium activation of GlyPb. We also sought to determine the minimum caffeine concentration required to fully inhibit GlyPa. This was achieved with 32 mM caffeine, as the specific activity was equal to inactive GlyPb given by the ANOVA test. These conditions were then used for the experiments to study nonequilibrium inhibition of GlyPa (Figures 1B and S2B,E).

In order to observe any kinetically populated transient structural ensembles, it is critical for the nonequilibrium experiments that the protein/ligand complex is fully saturated within 50 ms (dead time of the nonequilibrium experiment), governed by the second-order rate constant of association. In separate experiments, a range of AMP concentrations (2.5, 25, and 100 mM) and a range of caffeine concentrations (3, 10, and 32 mM) were assessed by rapid mixing with GlyPb or GlyPa, respectively, under nonequilibrium conditions (Figure 1C,D). The AMP and CFF binding sites were monitored by neHDX-MS at peptides T38–T47 and I275–F285, respectively. To confirm that a 1:1 complex with ligand had been reached, the degree of protection was compared with the end-state of the respective process: activated GlyPb fully equilibrated with 25 mM AMP or inhibited GlyPa fully equilibrated with caffeine. We concluded that within the neHDX-MS experiment dead time the ligand binding sites are saturated by AMP and CFF using the lowest concentrations shown to fully activate or inhibit (25 and 32 mM, respectively). Therefore, we proceeded to evaluate the transient structural kinetics that occur upon allosteric regulation across the whole enzyme.

### Transient Structural Dynamics during GlyPb Allosteric Activation by AMP

To identify specific dynamic changes associated with long-range allosteric communication induced by ligand binding, we developed a millisecond neHDX-MS approach that reveals the local structural perturbations that result from the conditions established above (Figure S1).

As the D-labeling of polypeptide backbone amide groups is a sensitive, but convoluted measure of H-bonding and solvent accessibility, first we sought to categorize the local differences in the protein ensemble during the nonequilibrium phase following allosteric activation by AMP/AS. Subtle changes in dynamics and structural changes can be derived from HDX-MS data, reflected in the observed kinetics of deuterium uptake plots at specific positions within the protein, given by short peptide segments in the “bottom-up” experiments used here.^49^ The data comprised measurements at 31 D-labeling time points over 4 orders of magnitude from 50 ms to 300 s for 219 peptide segments that cover 90.5% of the GlyP amino acid sequence (Figure S4A). This was done for three conditions: (i) inactive GlyPb (apo), (ii) fully activated GlyPb (termed GlyPb*) equilibrated for 1 h with 25 mM AMP, 25 mM AS, and (iii) GlyPb activated at nonequilibrium by rapid mixing with 25 mM AMP, 25 mM AS (termed GlyPb^). While many time points were collected, it is not necessary to observe the transient structural kinetics features that we describe here.

If the HDX labeling during the allosteric transition simply represented a changing population of R- and T-state protein, then the nonequilibrium trace would fall in between the other two lines and simply interpolate between them. Indeed, we observed this in some regions. However, for a variety of peptides throughout the protein the nonequilibrium trace is qualitatively different from both the apo and the activated states (Figures S5, S6, and S16). There are peptides for which the D-labeling values of the nonequilibrium state fall outside of the range measured for either the initial (apo) or equilibrated active (GlyPb*) forms, which indicates a degree of nonlinearity in the structural interpolation (Figures 3, S16, and S17). However, the submolecular location, magnitude, and nature of relative (de)protection of these peptides show a variety of differences.

Therefore, we next sought to classify these structural dynamic changes occurring during allosteric activation to better identify clear behaviors in each part of the enzyme.

**Figure 2:**
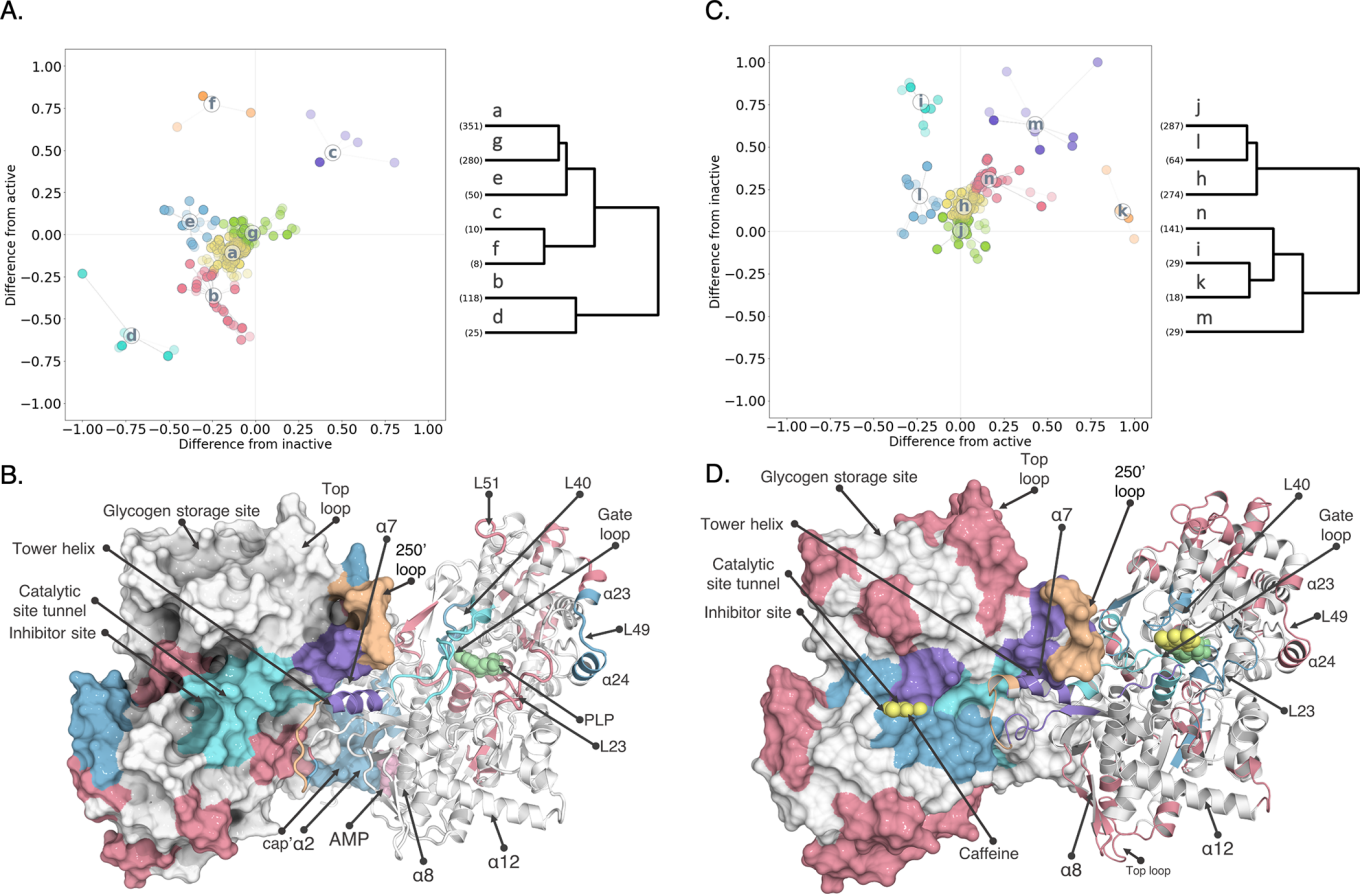
Clusters of structural kinetic behavior in GlyP. (A) Sum difference of the D uptake per amino acid between nonequilibrium and GlyPb/active state plotted against *X*/*Y-*axes, respectively. Amino acids were grouped into seven clusters (**a–g**) resulting from a *k*-means hierarchical analysis. The centroid of each cluster is denoted by a letter connected to each member by an edge. Raw D-labeling uptake data were centroided, corrected for maximum exchangeable amide hydrogens, normalized, and the different states were pairwise subtracted. Dendrogram of clustering in A is shown on the right. *Y*-axis is the Euclidean distance between the clusters (arbitrary units). The number of amino acid members in each cluster is indicated at the bottom of each cluster (truncated for clarity; Figure S15A). (B) Clusters (excluding **g** and **a**) represented the GlyPb active state structure (PDB:3E3N). Notable features are indicated on the left subunit molecular surface; notable secondary structural elements indicated. (C) Sum difference of the D uptake per amino acid between nonequilibrium and GlyPa/inactive state plotted against *X*/*Y*-axes, respectively. Amino acids were grouped into seven clusters (**h–n**), resulting from a *k*-means hierarchical analysis. The centroid of each cluster is denoted by the letter connected to each member by an edge. Raw D-labeling uptake data were centroided, corrected for maximum exchangeable amide hydrogens, normalized and the different states were pairwise subtracted. Dendrogram of clustering in C is shown on the right. *Y*-axis is Euclidean distance between the clusters (arbitrary units). The number of amino acid members in each cluster is indicated at the bottom of each cluster (truncated for clarity; Figure S15B). (D) Clusters (excluding **h** and **j**) are represented on the GlyPa inactive state structure (PDB:1GFZ). Notable features are indicated on the left subunit molecular surface; notable secondary structural elements indicated.

To categorize the local structural changes that occur during nonequilibrium activation/inhibition into correlated transient dynamics, we developed a simple, but robust quantitative analysis of the HDX-MS data. This served to represent the neHDX-MS kinetics for the pool of peptide segments and to act as input to a clustering algorithm, explained in detail in the SI Methods.^54,55^ After averaging peptide level neHDX-MS data to the amino acid level, each amino acid of the protein could be represented on a 2D plot. This created a spatial representation that reflects the relationship of the nonequilibrium state to the starting and ending states of the protein (Figure 2). We identified the nine archetypes of nonequilibrium structural kinetics that can be defined in this plot (Figure S9). In order to categorize the continuum of diverse D-labeling kinetic differences that we observed, we performed a *k*-means clustering analysis to identify amino acids with correlated HDX kinetics.

**Figure 3:**
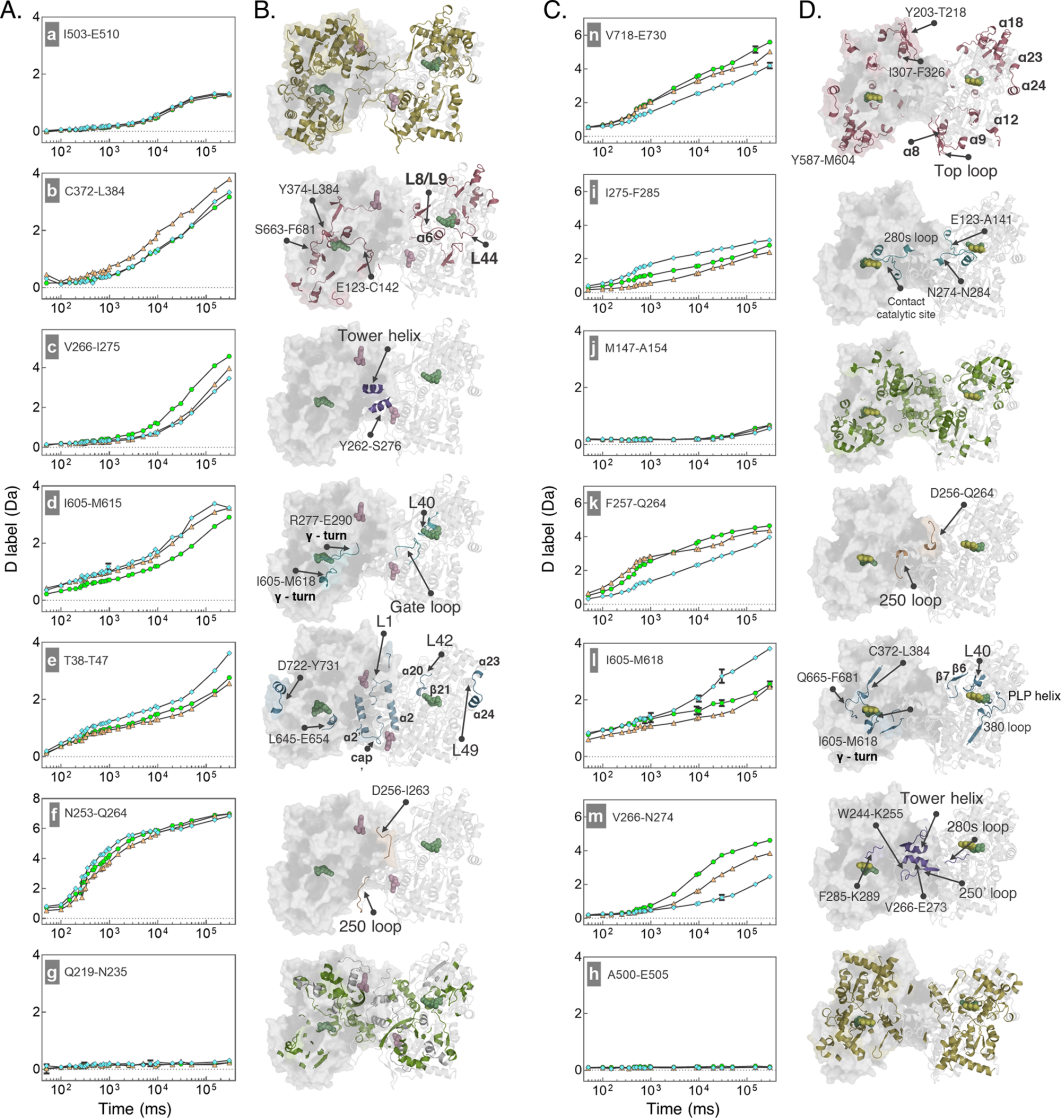
Local structural kinetics upon allosteric activation and inhibition revealed by nonequilibrium D-labeling. (A) Hydrogen-exchange kinetics of representative peptide segments for each cluster during activation of GlyPb. Mean of *n* = 3; error bars mostly contained within data points. GlyPb (blue); fully equilibrated activated with 25 mM AMP and 25 mM AS (orange); nonequilibrium activated with 25 mM AMP and 25 mM AS (green). (B) Clusters mapped on the crystal structure of inactive GlyPb (3E3N.pdb). (C) Hydrogen-exchange kinetics of representative peptide segments for each cluster during the inhibition of GlyPa. Mean of *n* = 3; error bars mostly contained within data points. GlyPa (blue); fully equilibrated inactivated with 32 mM caffeine (orange); nonequilibrium inactivated with 32 mM caffeine (green). (D) Clusters mapped on the crystal structure of inactive GlyPa (1GFZ.pdb).

Seven clusters of amino acids were identified within GlyP that comprise a common structural response to activation by AMP/AS (Figures 2 and S10). Figure 3A shows the D-labeling uptake plot for representative peptide segments from each cluster alongside the structural location of the cluster. The hierarchical relationship between the seven clusters was determined based on the observed transient structural changes (Figure 2A).^56^ This reveals that there are two closely related clusters (a and g) that show little or no observable transient structural change during allosteric activation (i.e., close to the origin in Figures 2A and S15). We calculated the local hydrogen-exchange rate (*k*_obs_) by fitting HDX-MS data to a stretched exponential model.^57^ This works well for many peptides, however, where multiple separate kinetic phases are observed in the same peptide the model fits less well.^58^ For most peptides, *k*_obs_ was low (*k*_obs_ < 1 × 10^–5^ s^–1^ for 130 of 219 peptides measured) in all three conditions (Tables S2 and S3), as GlyP is highly structured with many amino acids buried in the core of the enzyme with low solvent accessible surface area (SASA) and stable H-bond networks, consistent with previous studies.^19,29,57,59−62^ This validates that much of the enzyme is structurally identical before, during, and after allosteric activation, Figures S5, S6, and S16.

A third related cluster (**e**), representing the nucleotide binding site for AMP, shows no deviation from the active state and only transient relative protection compared to the inactive state that is on-pathway from the apo- to AMP-bound state (i.e., on *y* = 0 in Figure 2A). This group includes residues from α2 (residues 47–78), the cap’ region (residues 38–47) and residues from the nucleotide-binding domain (Figures 3A and S16). The helix α8 is also essential for AMP binding, but unfortunately, our coverage was lacking peptides in that region. There are two closely related clusters, which represent the 250′ loop (**f**) and the tower helix (**c**) that show transiently increased D-labeling compared to one or both equilibrium states. There are also two related clusters, representing the pyridoxal phosphate cofactor binding residues (**b**) and the 606 loop, that are distinct from all others, which show protection against D-labeling compared to both the inactive and the active state. The amino acids surrounding the pyridoxal phosphate (PLP) cofactor retain apo GlyPb HDX kinetics, indicating either that this region has a structural transition with a low probability or that the induced protection against HDX occurs too slowly to prevent the observed labeling.

A feature of the neHDX-MS data during AMP-activation is that there are transient structural changes that are distinct from both the inactive/active states (i.e., amino acids that do not lie on either *x* = 0 or *y* = 0 in Figure 2A) and therefore are not represented by high-resolution structural models derived from the equilibrium states. Activation of GlyPb by binding of AMP to the nucleotide site induces the well-established structural changes in the 250′ and 280s loops, together with the rotation of the tower helix (α7) (Figure S12). The neHDX-MS reveals previously unobserved transient changes that facilitate this. The 250′ loop (A248-G260) exhibits unique behavior during allosteric activation (cluster **f** in Figure 3A). The D-labeling kinetics interpolate smoothly between the GlyPb state (at *t* = 50 ms) and the active GlyPb* state (at *t* = 10 s). This indicates a likely two-state process for helical lengthening and that a gain of structure has fully equilibrated in the molecular population by 10 s of D-labeling, consistent with the tower helix in the active state lengthening at the N-terminal end by one turn in the GlyPb* state (3E3N.pdb^29^). Clusters **c** (the tower helix) and **d** (the 606 loop) show opposite structural behaviors: the 606 loop shows transient protection (increased H-bonding; reduced solvent accessibility), whereas the tower helix shows transient deprotection (i.e., local unfolding). The tower helix (G261-S276) is a contiguous alpha-helical structure in all known experimental models of apo, active, and inactive forms of GlyP. However, unexpectedly here we observe that it transiently loses protection against hydrogen/exchange, with increased observed D-labeling during allosteric activation following AMP binding at the nucleotide site (Figures 3A and S12).

**Figure 4:**
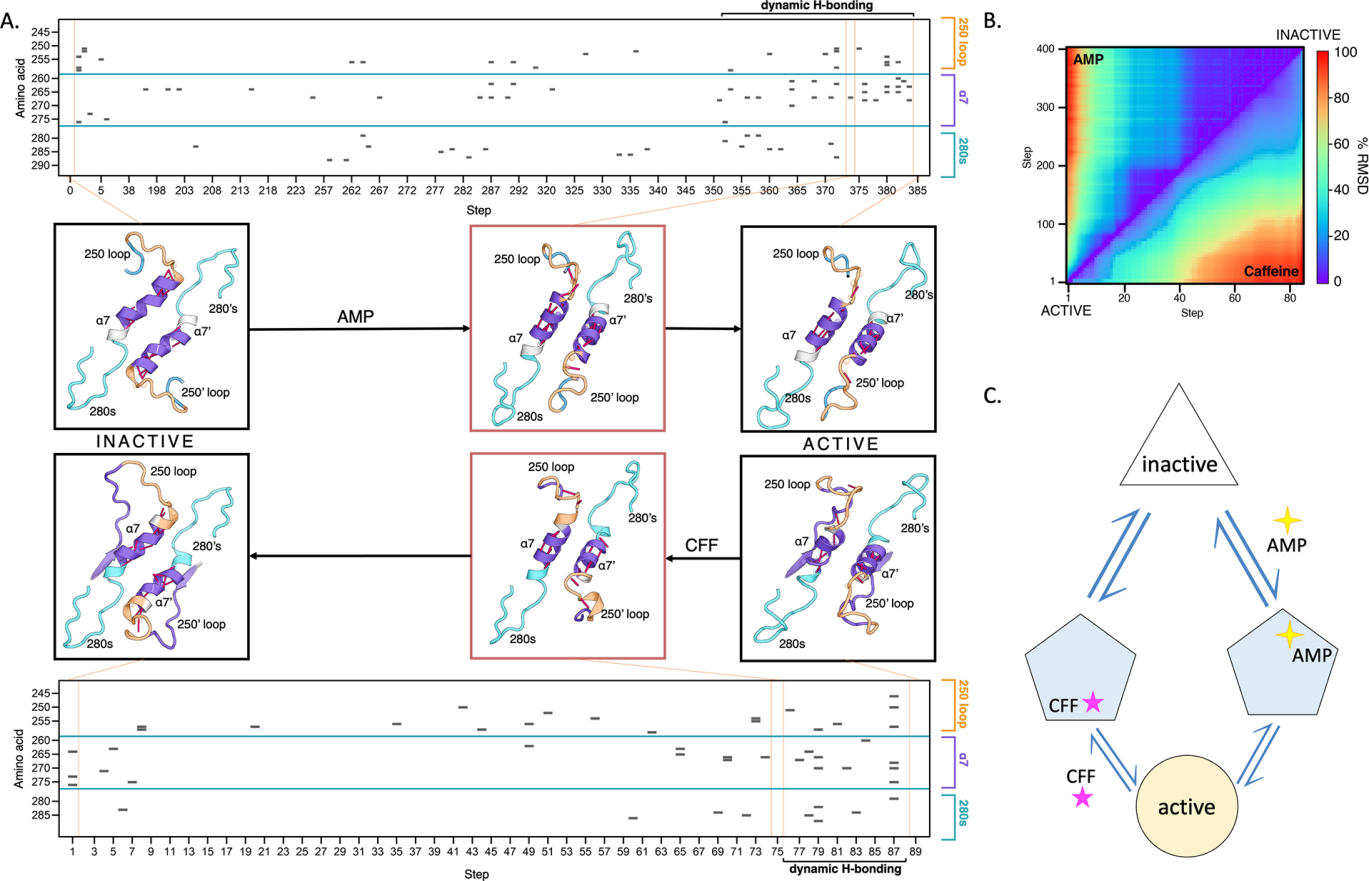
Structural interpolation between (in)active states of GlyP. (A) Tower helix and adjacent loops in inactive (left panel), transitional (middle), and active (right) conformations. Transitional structures with multiple broken hydrogen bonds (pink lines) in helix α7 are observed upon interpolation between apo GlyPb–GlyPb-AMP (activation) and between apo GlyPa and GlyPa-caffeine (inhibition). Representative structure shown; full trajectories provided as supplementary files. Heat maps show the breaking of H-bonds during steps along the trajectory of activation (top) and inhibition (bottom), gray: H-bond breaking event. Step refers to each pdb file output along the climber trajectory. (B) Interpolation trajectory is represented as RMSD between each structure. Calculated only for the tower helix region (amino acids 256:288). Normalized to maximum values from the AMP-activation (lower triangle) and caffeine inhibition (upper triangle), respectively. (C) Proposed model of the allosteric modification of GlyP. Both modifications, activation (AMP) and inhibition (CFF) of GlyPb and GlyPa, respectively, undergo a similar transition mechanism, with common structural features proximal to the active state.

We sought an atomistic rationale for this transient unfolding, so an all-atom interpolation method was used to generate structures on a nonlinear, energy minimized trajectory between apo GlyPb and GlyPb*. The Climber method has been shown to sample intermediate structures effectively, though has been difficult to validate until now, owing to the experimental challenge of determining transient local structural changes.^63^ Here a trajectory was created that interpolates between two crystal structures that have intact tower helices with predicted hydrogen bonds in conventional geometries, yet several of these hydrogen bonds are shown to undergo dynamic remodeling as the helices refold and rotate by ∼40° relative to each other to adopt the established active R-state, GlyPb* (Figures 4A and S12; Supplementary movies).^64^ The interpolation is consistent with the neHDX-MS data in which there is relative deprotection compared to both GlyPb and GlyPb* precisely at I263-D268, resolved by multiple overlapping peptide segments in the data, supporting an interpretation that there is transient unfolding in the tower helix during allosteric activation.

### Transient Structural Dynamics during GlyPa Allosteric Inhibition by Caffeine

We next considered whether this transient unfolding of the tower helix seen during activation is a feature common to the GlyP inhibition pathway. GlyPa phosphorylated at serine14 is basally active but is itself allosterically inhibited by the binding of caffeine, which stabilizes a T-state that is similar to the GlyPb apo state. The caffeine binding site is formed between the 280s and 606 loops, 3.5 nm from the nucleotide site, where AMP binding stabilizes the reverse transition to the R-state. Therefore, we next sought to identify the transient structural features on the inhibition pathway by performing neHDX-MS of GlyPa in the presence of caffeine. As for the AMP-activation experiments, this comprised measurements at 20 D-labeling time points 50 ms to 300 s resulting in 171 peptide segments that cover 91.4% of the GlyP amino acid sequence (Figure S4B). This was done for three conditions: (i) active GlyPa (apo), (ii) fully inhibited GlyPa (termed GlyPa′) equilibrated for 1 h with 32 mM caffeine, and (iii) GlyPa inhibited at nonequilibrium by rapid mixing with 32 mM caffeine (termed GlyPa^).

From a comparison of the observed HDX kinetics between GlyPa^ and each of the two equilibrated states, it is clear that the inhibition pathway involves a number of amino acids that show a transient structure distinct from both the start and end states (Figures 2C,D and S7, S8). Based on this, a clustering analysis identified amino acids with correlated HDX kinetics. As for AMP-activation, caffeine inhibition resulted in seven clusters (Figures S11 and S15) of amino acids within GlyP with a common structural response to ligand (Figures 2C and 3B). Again, much of the protein is relatively unchanged before, during, and after allosteric inhibition (clusters **h** and **j**, Figure 2C), which is consistent with the available crystal structures. The 280s and 606 loops form the binding site for caffeine, which intercalates between them in the GlyPa T-state crystal model. These amino acids are colocated in cluster **l**, which shows HDX equivalent to the GlyPa′ and protected compared to the GlyPa states, consistent with direct binding at this site without any observed transitional behavior (cluster **l,**Figures 2C,D and 3B). During caffeine inhibition, these three clusters (**h/j/l**) have minimal transient structural features and are found to be hierarchically unrelated to all other amino acids in the other four clusters (Figure 2C). Furthermore, the 250′ loop is highly deprotected against hydrogen exchange and labels more than the initial GlyPa state but similarly to the inhibited GlyPa′ state (cluster **k**). This is consistent with an interpretation that it is a two-state process, where the caffeine-inhibited T-state is observed immediately upon binding, in which one turn of the tower helix unwinds to extend the 250’ loop, as seen in the crystal models. The tower helix itself (cluster **m**) is labeled more with deuterium under nonequilibrium conditions (GlyPa^) than in either GlyPa or GlyPa’ states. As this is a highly solvent-exposed α helix in the initial R-state, it is most consistent with an interpretation of transient unfolding (Figure S13), again resolved by overlapping peptide segments to be most pronounced at amino acids L254–L271. The 280s loop shows diverged behavior, with the more C-terminal part forming part of the direct ligand binding site (cluster **l**) and with the N-terminal stretch showing transiently reduced hydrogen exchange compared to the initial apo GlyPa state but increased when compared to GlyPa′. This is consistent with the initial binding of caffeine to F285 and a subsequent propagation of the induced helical structure further upstream in the 280s loop as the tower helix lengthens by one turn at the C-terminal end.

It is apparent that a number of highly resolved transient structurally dynamic features are observed by neHDX-MS upon allosteric inhibition of GlyP by caffeine.

### Transient Unfolding of the Tower Helix is Common to Allosteric Activation and Inhibition Pathways

We next sought to compare the transient structural features observed upon AMP-activation and caffeine inhibition to determine whether these different ligands (and different allosteric sites) appear to act via different pathways or the same one. The 250′ and 280s loops show opposite neHDX-MS behavior in response to AMP and caffeine, which is logical given that these motifs are structurally different in the R/T-states and necessarily interpolate between them.

However, there is an unexpected common feature during both allosteric activation and inhibition, with transiently increased HDX observed in the tower helix that sits in between these two loops. Nonlinear interpolation between the atomistic models for GlyPa R-state (apo) and caffeine-bound (T-state) also predicts breakage, then reforming, of hydrogen bonds in the tower helix, as it does for AMP-activation (Figure 4A). It also predicts that the two structural pathways are closely related with low RMSD in the 250′ loop, tower helix, and 280s loop between structures close to the T-state and much larger differences in RMSD close to the R-state (Figure 4B). As the tower helix is highly solvent-exposed, the transiently lower protection factor would likely be the result of H-bond breakage, which is known to be a major determinant of the hydrogen-exchange rate. This is suggested by structural interpolation, which predicts these hydrogen bonds in the tower helix to be volatile in both pathways (Figure 4A).

These are the major transient structural features observed by neHDX-MS and appear common to both pathways, with a consistent atomistic rationalization given by the Climber structural interpolations.

## Discussion

### Transient Features of Allosteric Pathways Revealed

Here, we show that allosteric regulation of the central metabolic enzyme, GlyP, involves a number of dynamic changes in the local structure that are not observed in static structural models of the R- and T-states. We established a new protocol exploiting millisecond hydrogen/deuterium exchange at nonequilibrium to characterize transient changes in protection in response to ligand, resolved at near amino acid level throughout a 194 kDa enzyme. This revealed that AMP-activation induces a transient increase in the level of HDX in the tower helix. We rationalized this by interpolating the known structures for this allosteric transition using an atomistic method based on the Energy Calculation and Dynamics (ENCAD) molecular-mechanics force-field, Climber.^63^ This predicts the well-established lengthening and 40° relative rotation of the tower helices by first shortening, then bending it, wholly consistent with the increased hydrogen exchange observed during the process. Unexpectedly, caffeine inhibition—binding to a different allosteric site 3.5 nm away from AMP—also resulted in a transient increase in HDX in the tower helix. The structural interpolation indicates that this correlates with dynamic H-bonding, with considerable breaking of hydrogen bonds in the tower helix not seen elsewhere in the activation or inhibition trajectories (Figure 4A). This rationalizes the previous result that the tower helix and 280s loop are a highly conserved network between species.^65^ Therefore, both experiments are consistent with the interpretation suggested by the simulation that breaking of H-bonds in the tower helix is a common requirement of both AMP-activation and caffeine-inhibition pathways (Figure 4B,C.

### Slow (Millisecond) Labeling Can Observe Transient Structural Dynamics of the Molecular Population

It is perhaps unexpected that differences in neHDX-MS kinetics were observed across a range of time domains, from milliseconds to seconds. Structural kinetics were observed even at the fastest labeling time of 50 ms (Figures S5 and S7), in several significant regions, including 250 γ turn loop, β3 and α6, 280s loop and another γ turn with the central residue Tyr 613 said to be forming a hydrophobic sandwich of the nucleotide inhibitor site.^66^ At slower deuterium labeling times, significant changes in protection between the three states were observed throughout the α7 tower helix (residues 261–274), the β11b (residues 276–279), α23 (residues 714–725), and α24 (residues 728–735) (Figures S6 and S8). We interpret this range of observed nonequilibrium structural kinetics, milliseconds after ligand mixing, to indicate that the probability of the local structural changes is very low (i.e., bounded by a high-energy transition state), resulting in observation of the populated transient species (presumably transition states).

### Hydrogen/Deuterium-Exchange May Directly Observe VET Upon Ligand Binding

The HDX-MS intrinsic rate constants indicate a half-time of 100 s of milliseconds for which a protein structural state would need to exist in order for it to be appreciably detectable by this technique.^67^ However, our neHDX-MS data appear to observe transient species whose lifetimes would normally be considerably shorter than this. One explanation for this is that the VET, derived from the enthalpy of ligand binding, is anisotropically focused into a “hotspot,” which can undergo the HDX reaction faster than would be predicted because of an elevated local temperature. This would agree with our simulated trajectories where hydrogen-bond stability is locally reduced in the “hotspot” motif and with recent experimental evidence that VET is mediated by hydrogen-bond networks^18^ and with coarse-grained models of specific energy dissipation pathways in proteins.^68^ This may rationalize transient deprotection (i.e., observed increases in HDX labeling), but not transient protection, as seen in the 606 loop during AMP-induced activation. This could instead be explained by the nonequilibrium transition driving the molecular population through a narrow transition state ensemble. This would result in enhanced sampling of a high-energy conformation, which can involve local regions of higher or lower hydrogen exchange. Further work will be necessary to rationalize both the transient deprotection and the protection observed in the neHDX-MS data.

## Conclusions

We believe that this approach, while critically important to establish rapid saturation of the ligand binding site, provides a straightforward way to directly observe allosteric structural dynamics at nonequilibrium, which can be applied broadly to proteins irrespective of size or stability and with, in principle, any chemical or protein–ligand stimulus. In this manner, we expect it to be important in the future to understand not only what has changed in the protein structure but also how the changes originate and propagate through the molecule.

## Data Availability

The authors declare that the data supporting the findings of this study are available in this paper and its Supporting Information files. All additional data is available upon reasonable request. Source data are provided with this paper.

## Supporting Information Available

Tower helix during caffeine inhibition (MP4)
GlyPa inhibition by caffeine (MP4)
GlyPb activation by AMP (MP4)
Tower helix during AMP activation (MP4)
Experimental and computational methods, sample preparation, screening, HDX, data and statistical analysis, nonlinear structural interpolation, and analysis of protein trajectories (PDF)
Screening conditions, local HDX rates of GlyPb peptides, and local HDX rates of GlyPa peptides (XLSX)

## Notes

This study uses in-house developed scripts in Python. Available upon reasonable request.
