# Supplementary material for: Transient Structural Dynamics of Glycogen Phosphorylase from Nonequilibrium Hydrogen/Deuterium-Exchange Mass Spectrometry
Source: J Am Chem Soc. 2023 Dec 29;146(1):298–307. doi: 10.1021/jacs.3c08934 (PMC10786028; doi:10.1021/jacs.3c08934)
Supplement: Supplementary file 5 — ja3c08934_si_005.pdf [file ja3c08934_si_005.pdf]

Supplemental information

Transient structural dynamics of glycogen phosphorylase from non-equilibrium hydrogen/deuterium-exchange mass spectrometry

Monika Kish[1], Dylan P. Ivory[1], Jonathan J. Phillips[1,2]
[1]Living Systems Institute, Department of Biosciences, University of Exeter, Stocker Road, Exeter, EX4 4QD, UK
[2]Alan Turing Institute, British Library, London, NW1 2DB, UK

Table of figures:

Figure S1. Scheme for the continuous non-equilibrium HDX-MS approach. The protein sample is labelled at 20°C at an appropriate pH via three conditions, including apo, apo/ligand at equilibrium and apo/ligand at non-equilibrium. The difference from conventional HDX experiments is the non-equilibrium continuous labelling, achieved by placing the ligand in the labelling buffer, and only allowing them to interact for the preferred time. Upon quenching, digestion, separation and detection, the non-equilibrium data is analysed by clustering the peptide segments by their deuterium uptake difference from active/inactive state. Each cluster is then correlated to specific behaviour upon allosteric activation/inhibition. 5

Figure S2. (A) Glycogen hydrolysis kinetics for GlyPa (black), GlyPb (blue) and GlyPb* (orange; GlyPb equilibrated with 25 mM AMP and 25 mM AS). Absorbance (A.U.) at 30 ˚C for +60 min at 450 nm. Mean and SD from n=2 (B) Glycogen hydrolysis kinetics for GlyPa (black), GlyPb (blue) and GlyPa* (orange; GlyPa equilibrated with 32 mM caffeine. Absorbance (A.U.) at 30 ˚C for +60 min at 450 nm. Mean and SD from n=3. (C) Heat maps for structural perturbations in the presence of AMP/AS, detected by equilibrium HDX-MS for the nucleotide site (i), tower helix (ii) and 380s loop (iii). Deuterium uptake data from the HDX screen was used to produce these heat maps, n=3. The relative deuteration level for each sample is color coded shown on the scale right; note different normalization. (D) Catalytic activity of GlyPb supplemented with AS and AMP. Shaded region – GlyPa 95% confidence intervals. (E) Ammonium sulfate influence on the tower helix and nucleotide site. HDX labelling data was fitted as previously described and plotted versus the state (Figure S3). States annotated include: GlyPb - apo, * - equilibrium activated with 25 mM AMP and 25 mM AS, *AS - equilibrium activated with 25 mM AS, ^ eq AS – equilibrated with 25 mM AS and then non- equilibrium activated with AMP, ^ - non-equilibrium activated with 25 mM AMP and 25 mM AS, ^AMP – non-equilibrium activated with 25 mM AMP, ^AS – non- equilibrium activated with 25 mM AS. 6

Figure S3. Ammonium sulfate influence on the binding site residues 38-47. A. Raw data fits of each state, including GlyPb, GlyPb* - activated with 25/25 mM AMP/AS at equilibrium, GlyPb^ - activated with 25/25 mM AMP/AS at non-equilibrium, GlyPb^ 25 mM AMP - activated with 25 mM AMP at non-equilibrium, GlyPb^ 25 mM AS - activated with 25 mM AS at non-equilibrium, GlyPb^ preactivated 25 mM AS - activated with 25 mM AMP at non-equilibrium, but preactivated with 25 mM AS at equilibrium, GlyPb* 25 mM AS - activated with 25 mM AS at equilibrium. The fitted k_{obs} was used in further analysis (n=3). B. Raw uptake data of each state, per replicate, at 11 time points (50-300000 ms). Ammonium sulfate influence on the tower helix residues 266-275. C. Raw data fits of each state, including GlyPb, GlyPb* - activated with 25/25 mM AMP/AS at equilibrium, GlyPb^ - activated with 25/25 mM AMP/AS at non-equilibrium, GlyPb^ 25 mM AMP - activated with 25 mM AMP at non-equilibrium, GlyPb^ 25 mM AS - activated with 25 mM AS at non-equilibrium, GlyPb^ preactivated 25 mM AS - activated with 25 mM AMP at non-equilibrium, but preactivated with 25 mM AS at equilibrium, GlyPb* 25 mM AS - activated with 25 mM AS at equilibrium. The fitted k_{obs} was used in further analysis (n=3). D. Raw uptake data of each state, per replicate, at 11 time points (50-300000 ms). 7

Figure S4. Sequence coverage of glycogen phosphorylase from rabbit muscle. The peptides obtained via peptic digestion and LC-IMS/MS analysis are shown as black bars along the sequence numbers. Each bar under the sequence number annotation indicates an identified peptic peptide by eight MSE fragmentation experiments with different collision energy ramps, that was monitored during all HDX-MS experiments. A. These 219 peptides of glycogen phosphorylase b cover up to 800 amino acids of the total amino acid residues in the proteins, yielding a linear sequence coverage of up to 90.5%, with 3.24 redundancy. B. Similarly, 171 peptides of glycogen phosphorylase a, covering 91.4% of the sequence with 2.42% redundancy were identified and monitored during the HDX-MS experiments. 7

Figure S5. State D uptake difference maps for fast mixing times (50, 100, 150, 300 and 500 ms). Difference of the sum of observed deuterium uptake at 50, 100, 150, 300 and 500 ms between three states including GlyPb, GlyPb activated at equilibrium with 25/25 mM AMP/AS, and GlyPb activated at non-equilibrium with 25/25 mM AMP/AS. The deuterium uptake data from the state denoted on the bottom of each plot was subtracted from the data of the state denoted on the top in order to create the deuterium uptake difference plot. Relative protection leads to a more negative value (bar on bottom side); deprotection (e.g., from an exposed domain interface) results in a more positive value (bar on upper side). Each vertical bar represents a single peptide. Horizontal scale is peptides residues, from start residue (left) to end (right). The pink horizontal lines denote the significant difference between the states derived from the global significance threshold, explained elsewhere.[1, 2] Pink asterisks denote the significant difference in the D-label per peptide and time point derived from a T-test. 8

Figure S6. State D uptake difference maps for slow mixing times (1, 3, 10, 30 and 300 s). Difference of the sum of observed deuterium uptake at 1,3,10, 30 and 300 s between three states including GlyPb, GlyPb activated at equilibrium with 25/25 mM AMP/AS, and GlyPb activated at non-equilibrium with 25/25 mM AMP/AS. The deuterium uptake data from the state denoted on the bottom of each plot was subtracted from the data of the state denoted on the top in order to create the deuterium uptake difference plot. Relative protection leads to a more negative value (bar on bottom side); deprotection (e.g., from an exposed domain interface) results in a more positive value (bar on upper side). Each vertical bar represents a single peptide. Horizontal scale is peptides residues, from start residue (left) to end (right). The pink horizontal lines denote the significant difference between the states derived from the global significance threshold, explained elsewhere.[1, 2] Pink asterisks denote the significant difference in the D-label per peptide and time point derived from a T-test. 9
Figure S7. State D uptake difference maps for fast mixing times (50, 100, 150, 300 and 500 ms). Difference of the sum of observed deuterium uptake at 50, 100, 150, 300 and 500 ms between three states including GlyPa, GlyPb inhibitd at equilibrium with 32 mM caffeine, and GlyPb inhibitd at non-equilibrium with 32 mM caffeine. The deuterium uptake data from the state denoted on the bottom of each plot was subtracted from the data of the state denoted on the top in order to create the deuterium uptake difference plot. Relative protection leads to a more negative value (bar on bottom side); deprotection (e.g., from an exposed domain interface) results in a more positive value (bar on upper side). Each vertical bar represents a single peptide. Horizontal scale is peptides residues, from start residue (left) to end (right). The pink horizontal lines denote the significant difference between the states derived from the global significance threshold, explained elsewhere.[1, 2] Pink asterisks denote the significant difference in the D-label per peptide and time point derived from a T-test. 10
Figure S8. State D uptake difference maps for fast mixing times (1, 3, 10, 30 and 300 s). Difference of the sum of observed deuterium uptake at 1, 3, 10, 30 and 300 s between three states including GlyPa, GlyPb inhibitd at equilibrium with 32 mM caffeine, and GlyPb inhibitd at non-equilibrium with 32 mM caffeine. The deuterium uptake data from the state denoted on the bottom of each plot was subtracted from the data of the state denoted on the top in order to create the deuterium uptake difference plot. Relative protection leads to a more negative value (bar on bottom side); deprotection (e.g., from an exposed domain interface) results in a more positive value (bar on upper side). Each vertical bar represents a single peptide. Horizontal scale is peptides residues, from start residue (left) to end (right). The pink horizontal lines denote the significant difference between the states derived from the global significance threshold, explained elsewhere.[1, 2] Pink asterisks denote the significant difference in the D-label per peptide and time point derived from a T-test. 11
Figure S9. Theoretical behaviors of peptide segments upon non-equilibrium activation. Clustering by k means per peptide of the sum difference of the uptake data for the specific states after normalization involving non-equilibrium minus apo (x-axis), and non-equilibrium minus equilibrium (y-axis). Approximately, 9 clusters are anticipated, each cluster representing the location of the non-equilibrium kinetic uptake plot in respect to the apo and equilibrium uptake plots. From top to bottom the clusters include: c1 (x=-1, y=1), c2 (x=0, y=1), c3 (x=1, y=1), c4 (x=-1, y=0), c5 (x=0, y=0), c6 (x=1, y=0), c7 (x=-1, y=-1), c8 (x=0, y=-1), c9 (x=1, y=-1). Uptake kinetic plots are also represented for each cluster, where blue denotes apo state, orange ligand-bound at equilibrium and green the non-equilibrium state. 12
Figure S10. Assessing the quality of k-means clusters fit on the data of GlyPb. A. Elbow method for optimal value of clusters. B. Silhouette score analysis for clustering quality. The Silhouette score was calculated for the whole data set for 2-7 clusters. C. Silhouette analysis for 2-12 clusters. 13
Figure S11. Assessing the quality of k-means clusters fit on the data of GlyPa. A. Elbow method for optimal value of clusters. B. Silhouette score analysis for clustering quality. The Silhouette score was calculated for the whole data set for 2-7 clusters. C. Silhouette analysis for 2-12 clusters. 14
Figure S12. Rotation and transition of the tower helix upon AMP activation of GlyPb. A. Representation of the tower helix dynamics (residues 259-277), from T-state (blue), climber transition (green) and R-state (orange), with calculated angles between the α7 and α7' helices. Ramachandran plots are also included, calculated from the corresponding pdb files, with specific helix outliers highlighted. B. PDBs from the climber transition of GlyPb activation with AMP, including start pdb 000, 200, 450, 550, 600, 650, 688 and end state. The tower helices are colored cyan with the connecting 250 and 280s loops from both dimers. C. Difference plot representing the difference of the sum of uptake between the non-equilibrium GlyPb + AMP/AS data and the equilibrium GlyPb + AMP/AS data (pink), and the non-equilibrium GlyPb + AMP/AS data and GlyPb (cyan). D. The RMSD difference between the start (blue) and end (red) state with the 200, 450, 550, 600, 650, 688 PDBs. 15
Figure S13. Rotation and transition of the tower helix upon caffeine inhibition of GlyPa. A. Representation of the tower helix dynamics (residues 259-277), from T-state (blue), climber transition (green) and R-state (orange), with calculated angles between the α7 and α7' helices. Ramachandran plots are also included, calculated from the corresponding pdb files, with specific helix outliers highlighted. B. PDBs from the climber transition of GlyPa inhibition with caffeine, including start pdb 000, 020, 030, 040, 050, 060, 070, and end state. The tower helices are colored cyan with the connecting 250 and 280s loops from both dimers. C. Difference plot representing the difference of the sum of uptake between the non-equilibrium GlyPa + caffeine data and the equilibrium GlyPa + caffeine data (pink), and the non-equilibrium GlyPa + caffeine data and GlyPa (cyan). D. The RMSD difference between the start (blue) and end (red) state with the 020, 030, 040, 050, 060, 070 PDBs. 16
Figure S14. Representation of the full transition upon activation/inhibition. Each backbone nitrogen is represented as a sphere. Colors correspond to specific sections including the tower helix (yellow), 250 loop (pink) and 280 loop (blue). A. The movement of the backbone nitrogens upon AMP/AS activation. B. The movement of the backbone nitrogens upon caffeine inhibition. 17
Figure S15. Full dendrogram of hierarchical clustering. The dendrogram was generated with ScyPy for all identified peptides monitored by non-equilibrium HDX, where above p=7 in truncate mode, 7 clusters of behaviors were observed. The dendrogram illustrates how each cluster is composed by drawing a U-shaped link between a non-singleton cluster

and its children. The top of the U-link indicates a cluster merge. The two legs of the U-link indicate which clusters were merged. The length of the two legs of the U-link represents the distance between the child clusters. It is also the cophenetic distance between original observations in the two children clusters. 18
Figure S16. HDX uptake curves of GlyPb per peptide. Absolute data for three protein states shown (blue – apo GlyPb; orange – AMP-AS bound activated GlyPb; green – AMP-AS bound non-equilibrium activated GlyPb). Peptide sequence and amino acid number given above the plot. Y-axis shows absolute % deuteration at labeling time (ms) on X-axis. 19
Figure S17. HDX uptake curves of GlyPa per peptide. Absolute data for three protein states shown (blue – apo GlyPa; orange – caffeine bound activated GlyPa; green – caffeine bound non-equilibrium activated GlyPa). Peptide sequence and amino acid number given above the plot. Y-axis shows absolute % deuteration at labeling time (ms) on X-axis. 34

List of tables:

Table S1. Screening varying activating concentrations of AMP and Ammonium Sulfate. In total 25 samples were analyzed plus GlyPa, as an activated control. Both an enzyme activity kit and HDX were employed for the screen. 5
Table S2. Local hydrogen exchange rate (k_{obs}) of GlyPb peptides. Exchange rates were calculated by fitting a stretched exponential model to HDX-MS data with 31 time points for 219 peptide segments, for three conditions: (i) inactive GlyPb (apo), (ii) fully activated GlyPb (denoted eq) equilibrated for one hour with 25 mM AMP, 25 mM AS and (iii) GlyPb activated at non-equilibrium with 25 mM AMP, 25 mM AS (denoted by non-eq). 46
Table S3. Local hydrogen exchange rate (k_{obs}) of GlyPa peptides. Exchange rates were calculated by fitting a stretched exponential model to HDX-MS data with 20 time points for 171 peptide segments, for three conditions: (i) inactive GlyPa (apo), (ii) fully activated GlyPa (denoted eq) equilibrated for one hour with 32 mM caffeine,(iii) GlyPa activated at non-equilibrium with 32 mM caffeine (denoted by non-eq). 50

SUPPORTING METHODS

Sample preparation

Glycogen phosphorylase b and glycogen phosphorylase a were dissolved in phosphate buffered saline (PBS), pH 7.4 to 10.3 pmol/µL. For the equilibrium and non-equilibrium activated samples, the required amount of AMP/AS and caffeine were added, in the stock solution or the labelling buffer, respectively. During the screening experiments (HDX screen and enzyme activity assay) each sample was prepared simultaneously, with 10.3 pmol/µL GlyPb/GlyPa and with varying concentrations of AMP, AS or caffeine, Table S1.

Screening of conditions to activate GlyP by enzyme activity assay

The enzyme activity of GlyPb and GlyPa was screened for AMP/AS and caffeine dependence (Table S1). A colorimetric assay kit was used (ab273271), with only one adjustment of the protocol for GlyP sample preparation. A suitable protein concentration was determined by pre-testing a range of concentrations and plate reader settings, then all 25 samples were prepared in parallel at 1 mg/mL GlyP. Three relevant concentrations of caffeine were screened (3, 10 and 32 mM) to determine the extent of inhibition. Reaction kinetic curves and specific activity (mU/mG) were plotted and calculated according to the suggested protocol.

Screening of conditions to activate GlyP by HDX-MS

The observable perturbations in HDX were screened for AMP/AS dependence. A CTC PAL sample handling robot (LEAP Technologies, USA) was used to mix samples (Table S1) with deuterated PBS buffer in 1:20 ratio, then quench the labeling reaction by 1:1 mixing with 100 mM potassium phosphate to a final pH of 2.55 at 0 °C. Samples were immediately digested online with an Enzymate immobilized pepsin column (Waters) at 12 °C, the derived peptides trapped on a VanGuard 2.1 x 5 mm ACQUITY BEH C18 column (Waters) for 3 minutes at 125 µL/min at 0.5 °C and separated on a 1 × 100 mm ACQUITY BEH 1.7 µm C18 column (Waters) with a 7 min linear gradient of acetonitrile (5-40%) supplemented with 0.1% formic acid. Peptides were eluted into a Synapt G2-Si mass spectrometer (Waters).

Ammonium sulfate influence and maximum velocity kinetics

The maximum velocity kinetics and the AS influence on the HDX were determined prior continuing with the non-equilibrium experiments. HDX labelling experiments were performed at 11 time points, ranging from 50 ms to 300 s. AMP activation was tested at 3 different concentrations, including 2.5, 25 and 100 mM under non-equilibrium conditions with 25 mM AS. The AS influence on the equilibrated sample and the pre-equilibrated AS sample, was also determined. Analyzed protein states include: GlyPb - apo, * - equilibrium activated with 25 mM AMP and 25 mM AS, *AS - equilibrium activated with 25 mM AS, ^ eq AS – equilibrated with 25 mM AS and then non- equilibrium activated with AMP, ^ - non-equilibrium activated with 25 mM AMP and 25 mM AS, ^AMP – non-equilibrium activated with 25 mM AMP, ^AS – non- equilibrium activated with 25 mM AS. The deuterium uptake at labeling time *t*, was fitted to a stretched exponential[2,3] where nexp is a user-defined number of exponential phases, *N* is the maximum number of labile hydrogens, k_{obs} is the observed exchange rate constant, and β is a stretching factor:

$$\mathrm{D_t} = \mathrm{N} \sum_{i=1}^{\mathrm{nexp}} 1 - e^{\left[-(\mathrm{k_{obs,i}} \mathrm{t})^{\beta_i}\right]}$$

Hydrogen-deuterium exchange mass spectrometry

HDX was performed using a fully-automated, millisecond HDX labelling and online quench-flow instrument, ms2min (Applied Photophysics Ltd), connected to an HDX manager (Waters). During the labelling experiments, 14 µL of either apo or equilibrated GlyPb/GlyPa were delivered to the labelling mixer. A 20-fold dilution with labelling buffer at 20˚C commenced the HDX. Depending on the desired experiment, the labelling buffer differed significantly. For apo and equilibrium experiments, the labelling buffer was 1 x PBS, pH_{read} =7.00 at 20˚C. During non-equilibrium experiments and other assessments (see Error! Reference source not found.), the labelling buffer consisted of 1 x PBS, pH =7.00 at 20˚C combined with 25 mM AMP/25 mM AS or 32 mM caffeine. All GlyPb samples were labelled at 30 time points, ranging from 50 ms to 300 s. All GlyPa samples were labelled at 20 time points, ranging from 50 ms to 300 s. Immediately after, the HDX reaction was quenched by mixing with quench buffer (100 mM Potassium phosphate, pH =2.5 at 0˚C) at 1:1 ratio, and sample digested online with an Enzymate immobilized pepsin column (Waters), the derived peptides trapped on a VanGuard 2.1 x 5 mm ACQUITY BEH C18 column (Waters) for 3 minutes at 125 µL/min and separated on a 1 × 100mm ACQUITY BEH 1.7 µm C18 column (Waters) with a 7-minute linear gradient of acetonitrile (5-40%) supplemented with 0.1% formic acid. Peptides were eluted into a Synapt G2-Si mass spectrometer (Waters). Mass spectra were obtained using the Waters $HDMS^E$ mode within a mass range of 50 to 2000 m/z. Instrument parameters were configured as follows: a capillary voltage of 3.0 kV, a cone voltage of 50 V, a trap collision energy of 4 V, a traveling wave ion mobility separation at a velocity of 475 m/s, a wave amplitude of 36.5 V, and a nitrogen pressure of 2.75 mbar. For low-energy scans, a transfer collision energy of 4 V was applied, while high-energy scans utilized four separate collision energy ramps ranging from 15 to 55 V.

Data and statistical analysis

PLGS (ProteinLynx Global Server 2.5.1, Waters) was used for the analysis of MS^E reference data and identification of all discoverable peptic peptides. DynamX 3.0 (Waters, USA) was used for processing and assignments of isotopic distributions of all raw data files. All HDX-MS experiments were performed in triplicate (technical replicates). The means and standard deviations (SD) of these replicates were used to determine the significance of changes, global significance threshold, as previously described[1]. In-house developed scripts in Python 3.4.0 (Python Software Foundation) were used for all post-processing analyses, clustering analyses, and plotting in PyMOL (Schrödinger, Inc.). A clustering analysis was employed to investigate the behavior of the peptide during non-equilibrium activation/inhibition. The sum of deuterium uptake over all time points for each peptide was calculated for all three protein conditions (states) of GlyPa or GlyPb, in each case. The D-labeling data for each peptide segment was expressed in % to account for varying peptide length and presence of proline for which there is no signal. Similarly, the data was divided by the number of time points. The values for the non-eq data were then subtracted from both other states for every peptide segment in the data set, resulting in an [n x 2] vector of 2-d coordinates. The peptide level data were then flattened per amino acid, as previously described[2]. The resilience of this process to error depends strongly on the redundancy of the peptide map, as the averaging process will combine behavior of overlapping peptides in the amino acids shared between them, potentially skewing interpretation of any single amino acid. With the non-equilibrium measurements defined as 2-d coordinates relative to a start/end state, this constitutes a well-formed input for a k-means clustering analysis. The 2-d coordinates for HDX-MS differences were then *k-means* clustered with *k* clusters indicated to be optimal by both an elbow method and a Silhouette analysis.

Non-linear atomistic structural interpolation

The Climber program was utilized for stepwise and non-linear structural interpolation between the active and inactive conformations using default parameters. This involved dynamic evaluation of RMSD between backbone and side chain atom pairs, with continual updates. Atom pair sets were created for each generated conformation between the initial (A) and final (B) states, based on criteria of distance between alpha carbons (>10 Å) and side chain atom pairs (<10 Å). Harmonic restraints were applied to steer inter-residue distances towards the target conformation. The force constant for conformational transformation was adjusted based on RMSD progress. Morphed conformations underwent iterative minimization using the ENCAD potential energy function. Initial coordinates for the allosteric activation and inhibition were taken from crystallographic structures of glycogen phosphorylase b and glycogen phosphorylase a. PDB ID 1gpb and 3e3n were used for GlyPb activation, and PDB ID 1gpa and 1c8l for GlyP inhibition. Missing residues (251-259, 315-324 in 1c8l; 251-260, 281-287 in 3e3n) were built and optimized using MODELLER (version 10.4) [4,5]. For each loop region, a total of 100 conformations were generated, and the model exhibiting the lowest value for MODELLER's objective was chosen as the final protein structure.

Graph network analysis of protein trajectories

The n PDB files output by Climber were compared recursively with each other to calculate [n x n] pairwise rmsd values. For plotting, the desired amino acids (i.e. the 250' loop – tower helix – 280s loop) were extracted beforehand and the calculation was run only on those and the values normalized to the observed maximum. Plotting of the rmsd trajectory was done in Matplotlib. A graph network was constructed by selecting ten pdb files equidistant on the trajectory (with respect to number of steps taken to complete interpolation in Climber) and a Spring layout model was used to plot the 2-d projection with the network Python library.

Table S1. Screening varying activating concentrations of AMP and Ammonium Sulfate. In total 25 samples were analyzed plus GlyPa, as an activated control. Both an enzyme activity kit and HDX were employed for the screen.

$(NH_4)_2SO_4$ (mM)	100	✓	✓	✓	✓
	75	✓	✓	✓	✓
	50	✓	✓	✓	✓
	25	✓	✓	✓	✓
	0	25	50	75	100
AMP (mM)					

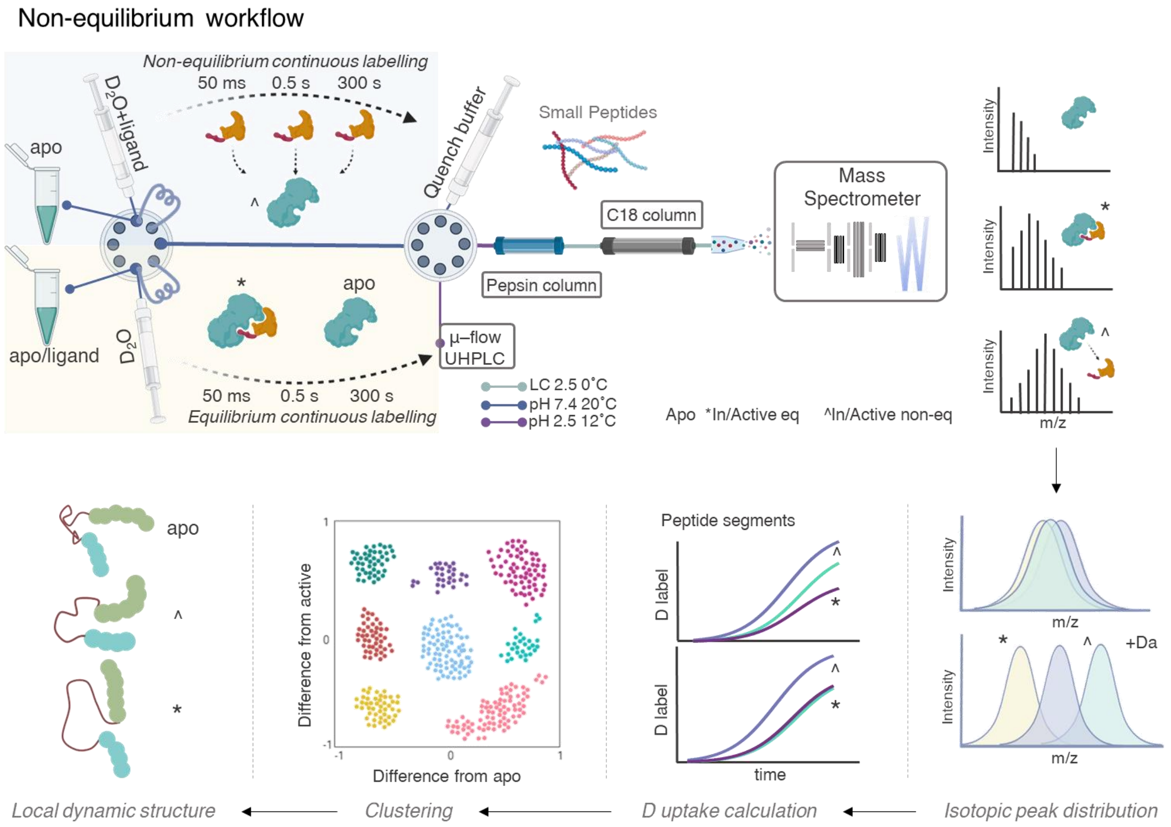

Figure S1. Scheme for the continuous non-equilibrium HDX-MS approach. The protein sample is labelled at 20°C at an appropriate pH via three conditions, including apo, apo/ligand at equilibrium and apo/ligand at non-equilibrium. The difference from conventional HDX experiments is the non-equilibrium continuous labelling, achieved by placing the ligand in the labelling buffer, and only allowing them to interact for the preferred time. Upon quenching, digestion, separation and detection, the non-equilibrium data is analyzed by clustering the peptide segments by their deuterium uptake difference from active/inactive state. Each cluster is then correlated to specific behaviour upon allosteric activation/inhibition.

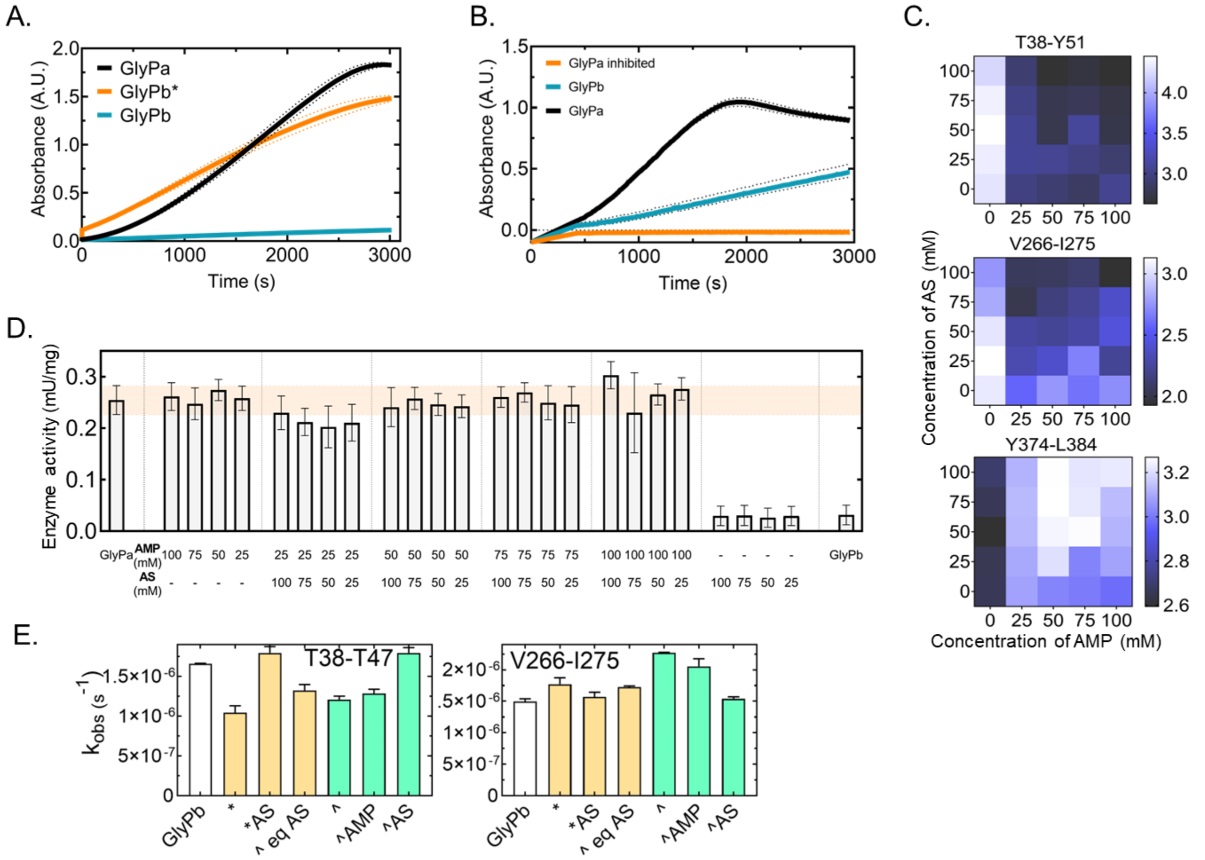

Figure S2. (**A**) Glycogen hydrolysis kinetics for GlyPa (black), GlyPb (blue) and GlyPb* (orange; GlyPb equilibrated with 25 mM AMP and 25 mM AS). Absorbance (A.U.) at 30 ˚C for +60 min at 450 nm. Mean and SD from n=2 (**B**) Glycogen hydrolysis kinetics for GlyPa (black), GlyPb (blue) and GlyPa* (orange; GlyPa equilibrated with 32 mM caffeine. Absorbance (A.U.) at 30 ˚C for +60 min at 450 nm. Mean and SD from n=3. (**C**) Heat maps for structural perturbations in the presence of AMP/AS, detected by equilibrium HDX-MS for the nucleotide site (top), tower helix (middle) and 380s loop (bottom). Deuterium uptake data from the HDX screen was used to produce these heat maps, n=3. The relative deuteration level for each sample is color coded shown on the scale right; note different normalization. (**D**) Catalytic activity of GlyPb supplemented with AS and AMP. Shaded region – GlyPa 95% confidence intervals. (**E**) Ammonium sulfate influence on the tower helix and nucleotide site. HDX labelling data was fitted as previously described and plotted versus the state (Figure S3). States annotated include: GlyPb - apo, * - equilibrium activated with 25 mM AMP and 25 mM AS, *AS - equilibrium activated with 25 mM AS, ^ eq AS – equilibrated with 25 mM AS and then non- equilibrium activated with AMP, ^ - non-equilibrium activated with 25 mM AMP and 25 mM AS, ^AMP – non-equilibrium activated with 25 mM AMP, ^AS – non- equilibrium activated with 25 mM AS.

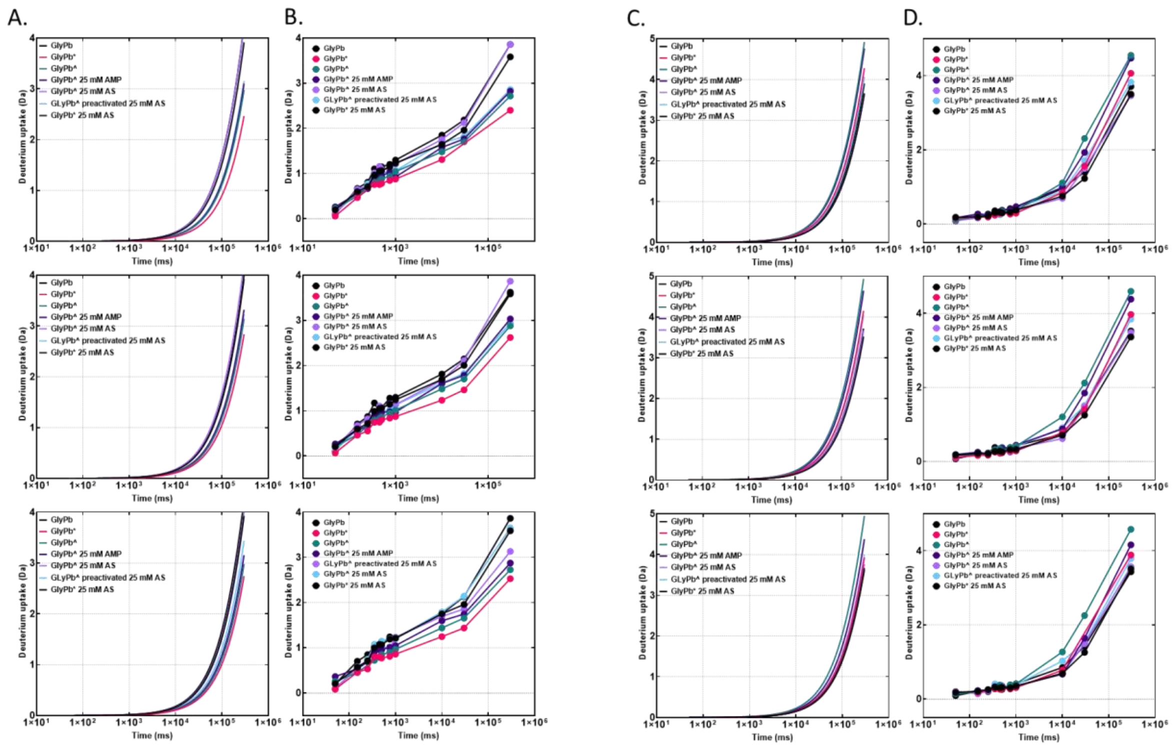

Figure S3. Ammonium sulfate influence on the binding site residues 38-47. A. Raw data fits of each state, including GlyPb, GlyPb* - activated with 25/25 mM AMP/AS at equilibrium, GlyPb^ - activated with 25/25 mM AMP/AS at non-equilibrium, GlyPb^ 25 mM AMP - activated with 25 mM AMP at non-equilibrium, GlyPb^ 25 mM AS - activated with 25 mM AS at non-equilibrium, GlyPb^ preactivated 25 mM AS - activated with 25 mM AMP at non-equilibrium, but preactivated with 25 mM AS at equilibrium, GlyPb* 25 mM AS - activated with 25 mM AS at equilibrium. The fitted k_{obs} was used in further analysis (n=3). B. Raw uptake data of each state, per replicate, at 11 time points (50-300000 ms). Ammonium sulfate influence on the tower helix residues 266-275. C. Raw data fits of each state, including GlyPb, GlyPb* - activated with 25/25 mM AMP/AS at equilibrium, GlyPb^ - activated with 25/25 mM AMP/AS at non-equilibrium, GlyPb^ 25 mM AMP - activated with 25 mM AMP at non-equilibrium, GlyPb^ 25 mM AS - activated with 25 mM AS at non-equilibrium, GlyPb^ preactivated 25 mM AS - activated with 25 mM AMP at non-equilibrium, but preactivated with 25 mM AS at equilibrium, GlyPb* 25 mM AS - activated with 25 mM AS at equilibrium. The fitted k_{obs} was used in further analysis (n=3). D. Raw uptake data of each state, per replicate, at 11 time points (50-300000 ms).

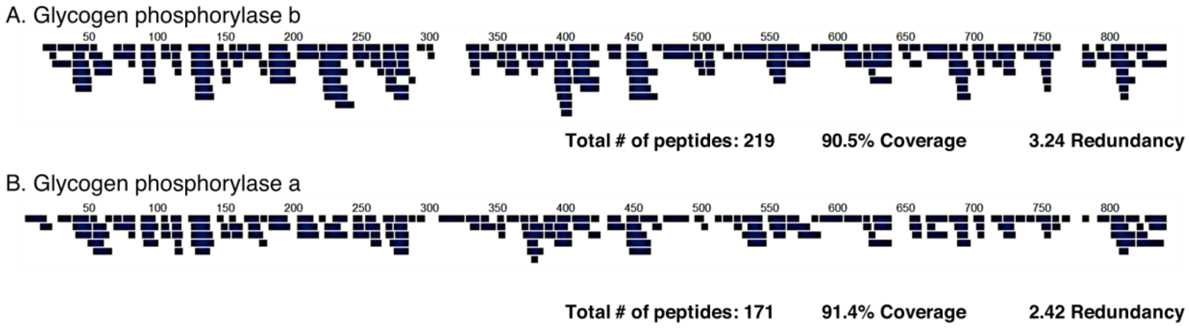

Figure S4. Sequence coverage of glycogen phosphorylase from rabbit muscle. The peptides obtained via peptic digestion and LC-IMS/MS analysis are shown as black bars along the sequence numbers. Each bar under the sequence number annotation indicates an identified peptic peptide by eight MSE fragmentation experiments with different collision energy ramps, that was monitored during all HDX-MS experiments. A. These 219 peptides of glycogen phosphorylase b cover up to 800 amino acids of the total amino acid residues in the proteins, yielding a linear sequence coverage of up to 90.5%, with 3.24 redundancy. B. Similarly, 171 peptides of glycogen phosphorylase a, covering 91.4% of the sequence with 2.42% redundancy were identified and monitored during the HDX-MS experiments.

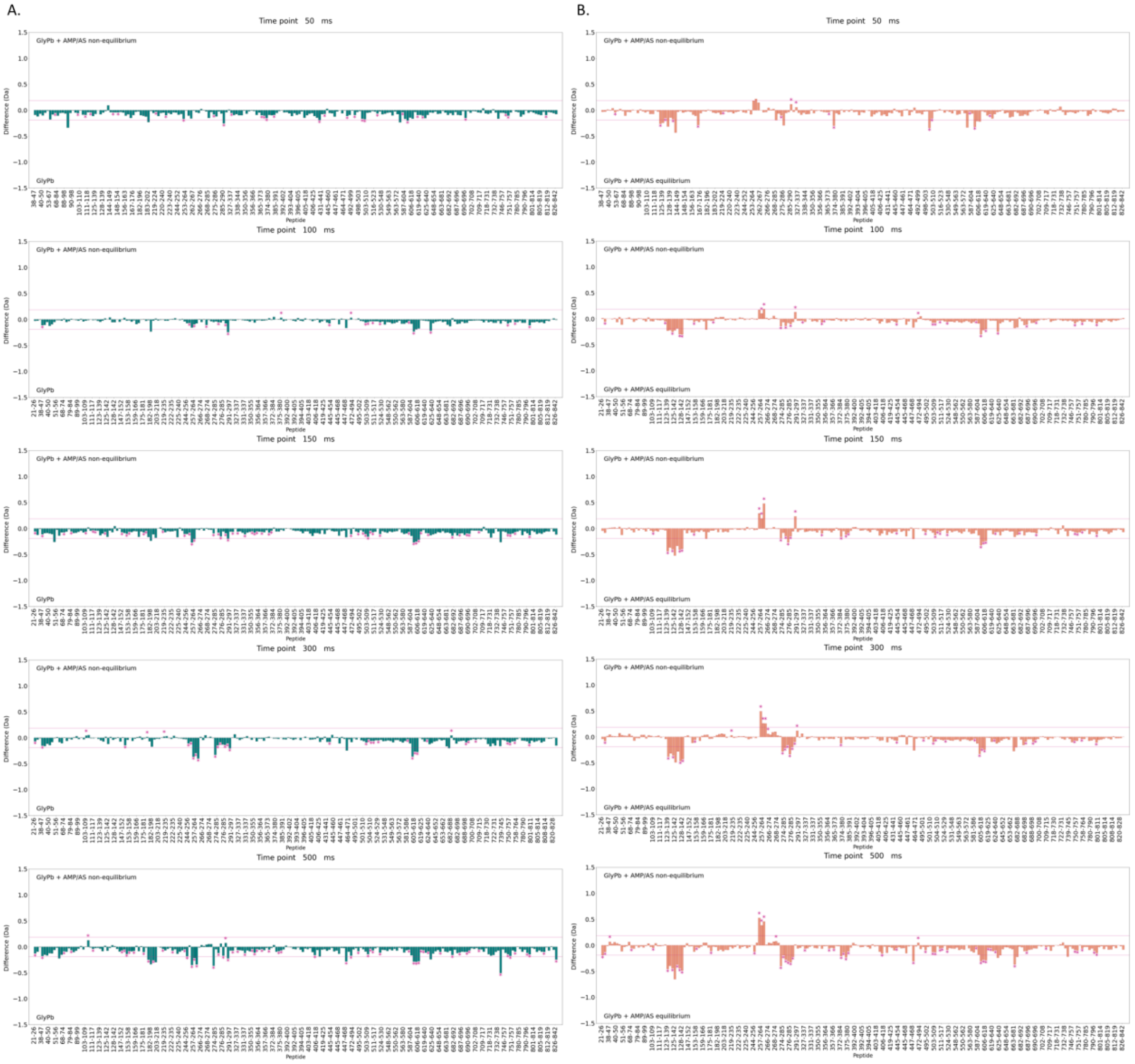

Figure S5. State D uptake difference maps for fast mixing times (50, 100, 150, 300 and 500 ms). Difference of the sum of observed deuterium uptake at 50, 100, 150, 300 and 500 ms between three states including GlyPb, GlyPb activated at equilibrium with 25/25 mM AMP/AS, and GlyPb activated at non-equilibrium with 25/25 mM AMP/AS. The deuterium uptake data from the state denoted on the bottom of each plot was subtracted from the data of the state denoted on the top in order to create the deuterium uptake difference plot. Relative protection leads to a more negative value (bar on bottom side); deprotection (e.g., from an exposed domain interface) results in a more positive value (bar on upper side). Each vertical bar represents a single peptide. Horizontal scale is peptides residues, from start residue (left) to end (right). The pink horizontal lines denote the significant difference between the states derived from the global significance threshold, explained elsewhere.[1, 2] Pink asterisks denote the significant difference in the D-label per peptide and time point derived from a T-test.

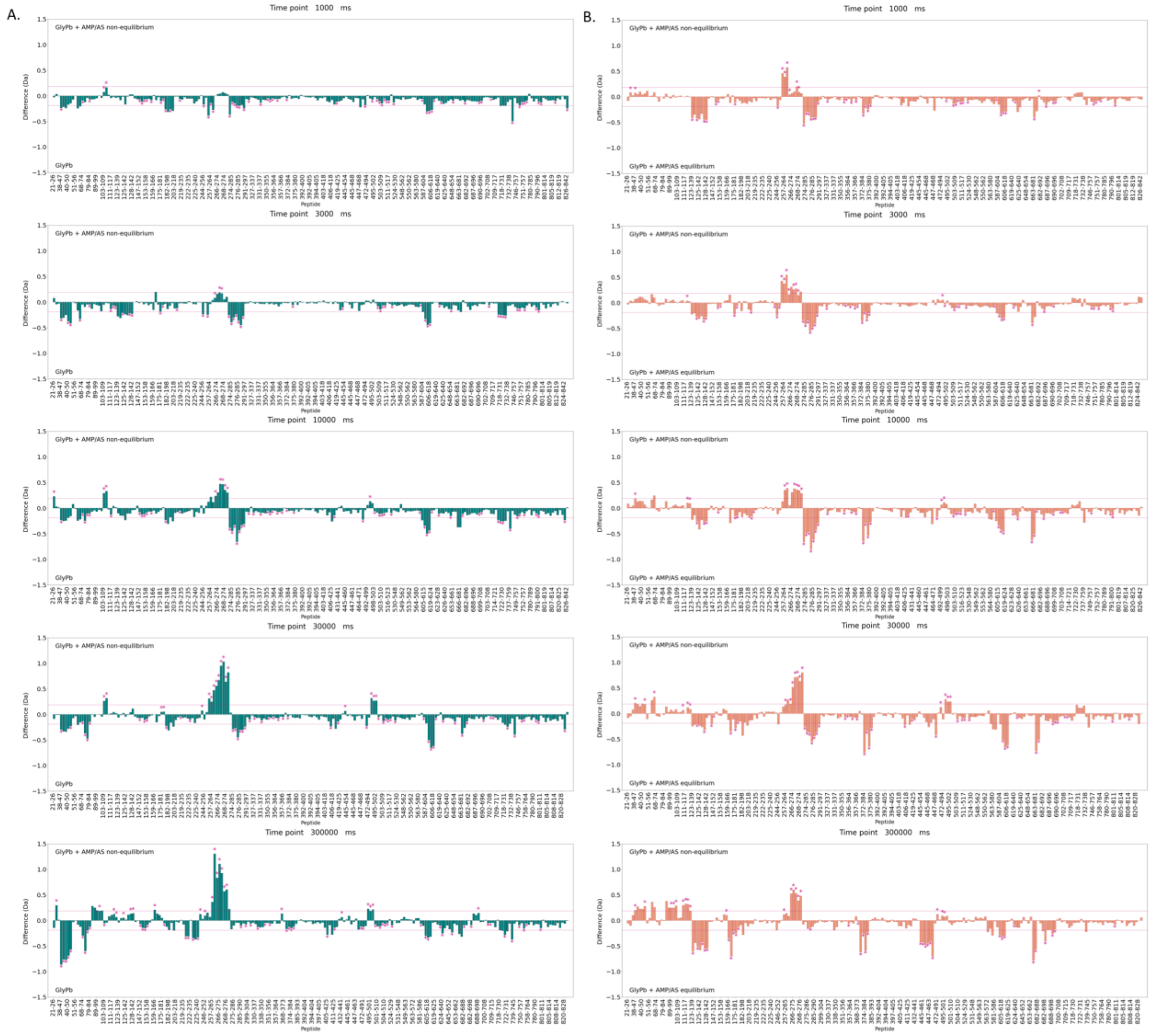

Figure S6. State D uptake difference maps for slow mixing times (1, 3, 10, 30 and 300 s). Difference of the sum of observed deuterium uptake at 1,3,10, 30 and 300 s between three states including GlyPb, GlyPb activated at equilibrium with 25/25 mM AMP/AS, and GlyPb activated at non-equilibrium with 25/25 mM AMP/AS. The deuterium uptake data from the state denoted on the bottom of each plot was subtracted from the data of the state denoted on the top in order to create the deuterium uptake difference plot. Relative protection leads to a more negative value (bar on bottom side); deprotection (e.g., from an exposed domain interface) results in a more positive value (bar on upper side). Each vertical bar represents a single peptide. Horizontal scale is peptides residues, from start residue (left) to end (right). The pink horizontal lines denote the significant difference between the states derived from the global significance threshold, explained elsewhere.[1, 2] Pink asterisks denote the significant difference in the D-label per peptide and time point derived from a T-test.

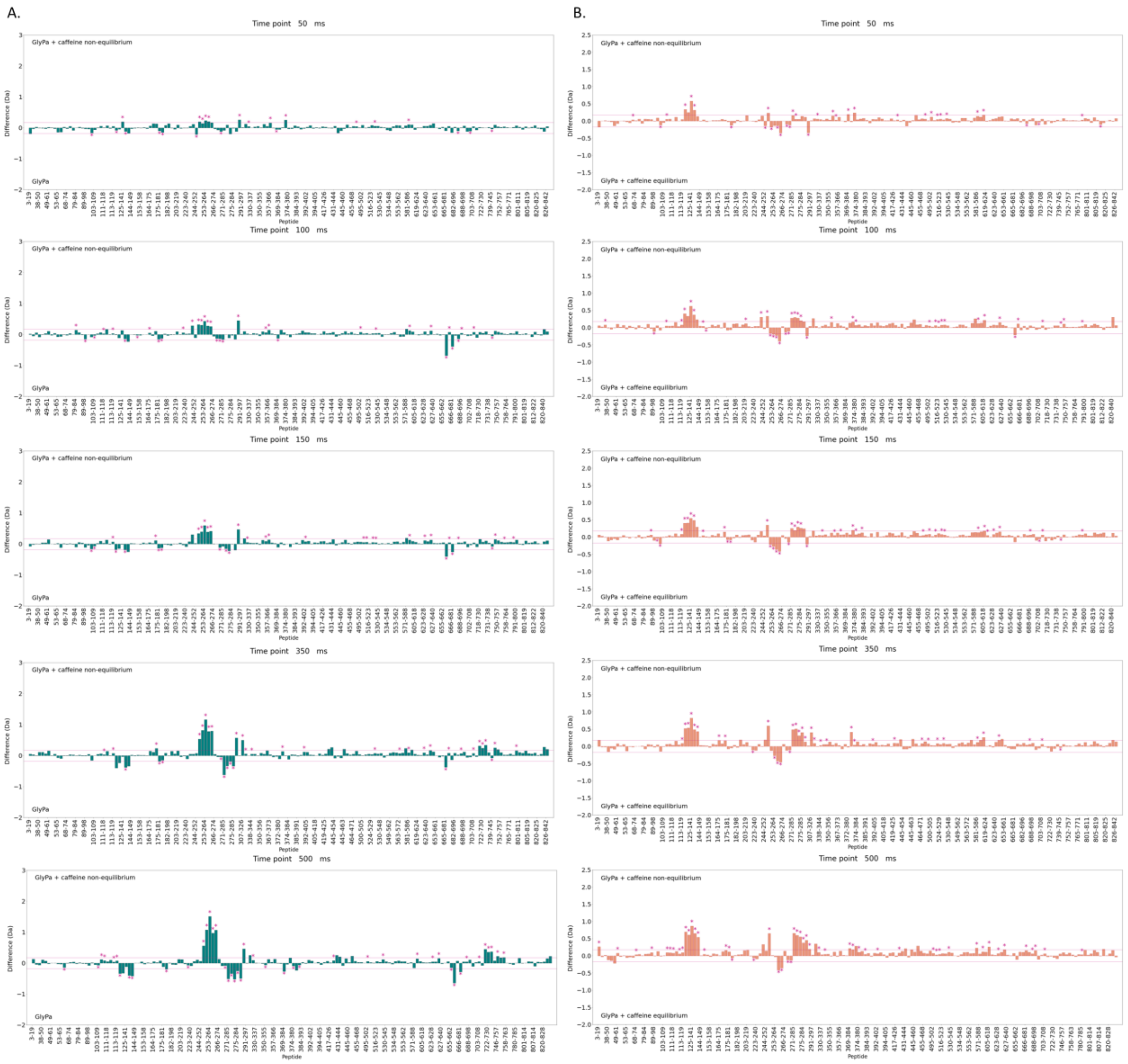

Figure S7. State D uptake difference maps for fast mixing times (50, 100, 150, 300 and 500 ms). Difference of the sum of observed deuterium uptake at 50, 100, 150, 300 and 500 ms between three states including GlyPa, GlyPb inhibited at equilibrium with 32 mM caffeine, and GlyPb inhibited at non-equilibrium with 32 mM caffeine. The deuterium uptake data from the state denoted on the bottom of each plot was subtracted from the data of the state denoted on the top in order to create the deuterium uptake difference plot. Relative protection leads to a more negative value (bar on bottom side); deprotection (e.g., from an exposed domain interface) results in a more positive value (bar on upper side). Each vertical bar represents a single peptide. Horizontal scale is peptides residues, from start residue (left) to end (right). The pink horizontal lines denote the significant difference between the states derived from the global significance threshold, explained elsewhere.[1,2] Pink asterisks denote the significant difference in the D-label per peptide and time point derived from a T-test.

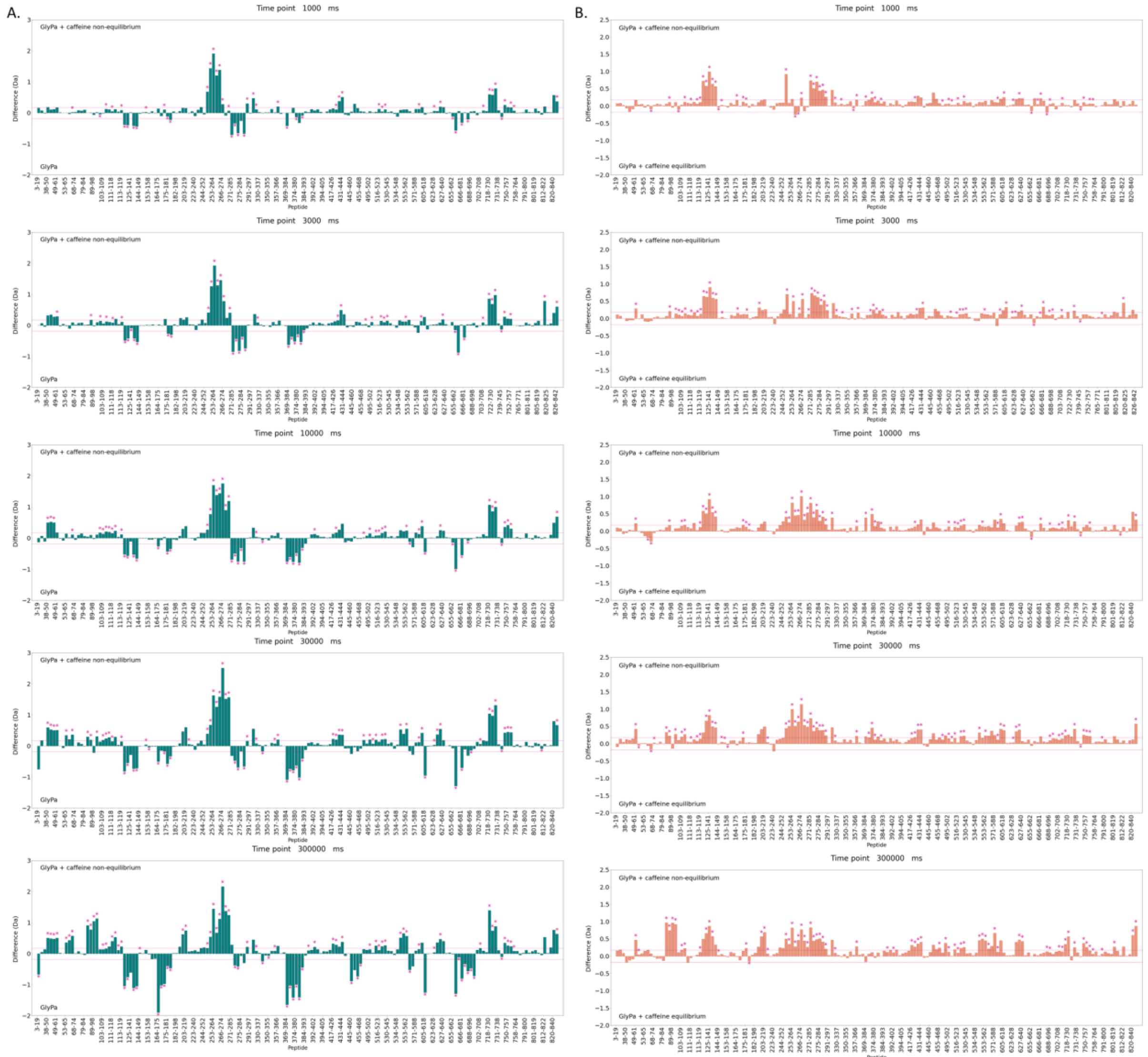

Figure S8. State D uptake difference maps for fast mixing times (1, 3, 10, 30 and 300 s). Difference of the sum of observed deuterium uptake at 1, 3, 10, 30 and 300 s between three states including GlyPa, GlyPb inhibited at equilibrium with 32 mM caffeine, and GlyPb inhibited at non-equilibrium with 32 mM caffeine. The deuterium uptake data from the state denoted on the bottom of each plot was subtracted from the data of the state denoted on the top in order to create the deuterium uptake difference plot. Relative protection leads to a more negative value (bar on bottom side); deprotection (e.g., from an exposed domain interface) results in a more positive value (bar on upper side). Each vertical bar represents a single peptide. Horizontal scale is peptides residues, from start residue (left) to end (right). The pink horizontal lines denote the significant difference between the states derived from the global significance threshold, explained elsewhere.[1,2] Pink asterisks denote the significant difference in the D-label per peptide and time point derived from a T-test.

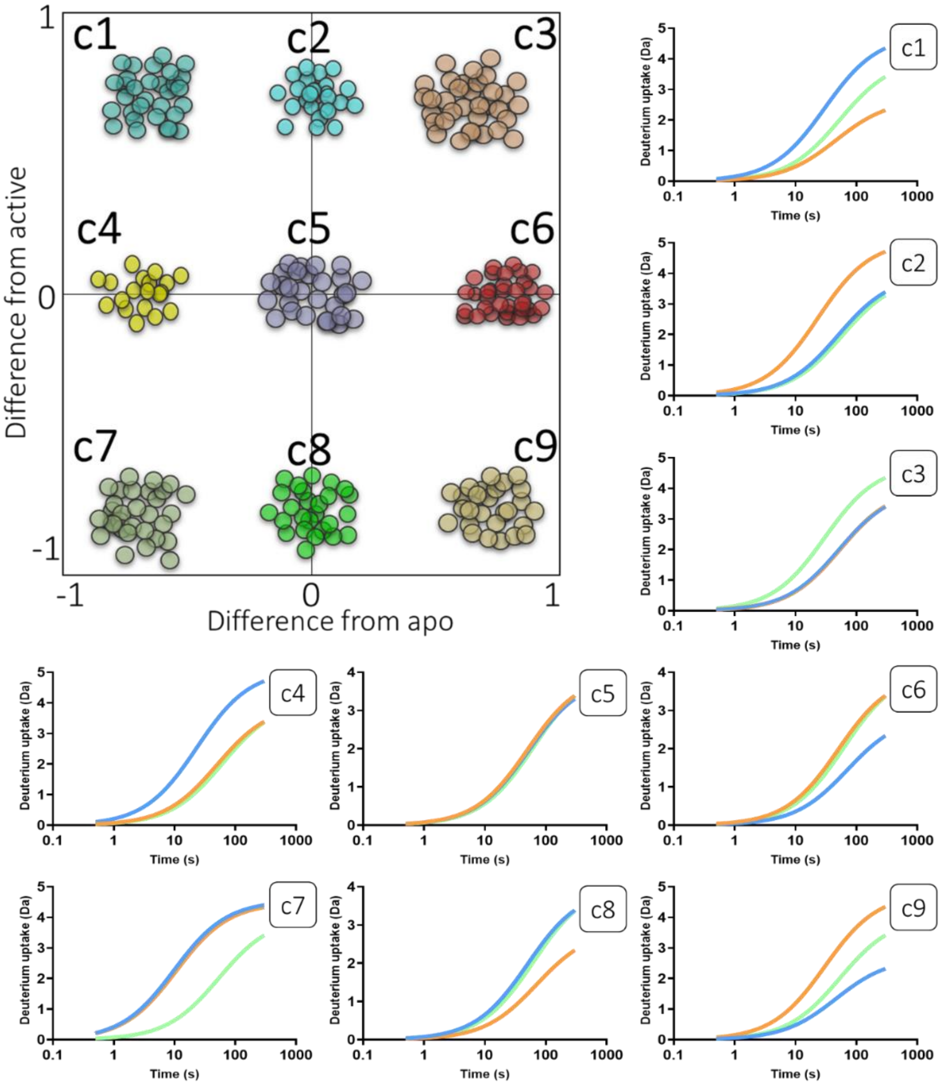

Figure S9. Theoretical behaviors of peptide segments upon non-equilibrium activation. Clustering by k means per peptide of the sum difference of the uptake data for the specific states after normalization involving non-equilibrium minus apo (x-axis), and non-equilibrium minus equilibrium (y-axis). Approximately, 9 clusters are anticipated, each cluster representing the location of the non-equilibrium kinetic uptake plot in respect to the apo and equilibrium uptake plots. From top to bottom the clusters include: c1 (x=-1, y=1), c2 (x=0, y=1), c3 (x=1, y=1), c4 (x=-1, y=0), c5 (x=0, y=0), c6 (x=1, y=0), c7 (x=-1, y=-1), c8 (x=0, y=-1), c9 (x=1, y=-1). Uptake kinetic plots are also represented for each cluster, where blue denotes apo state, orange ligand-bound at equilibrium and green the non-equilibrium state.

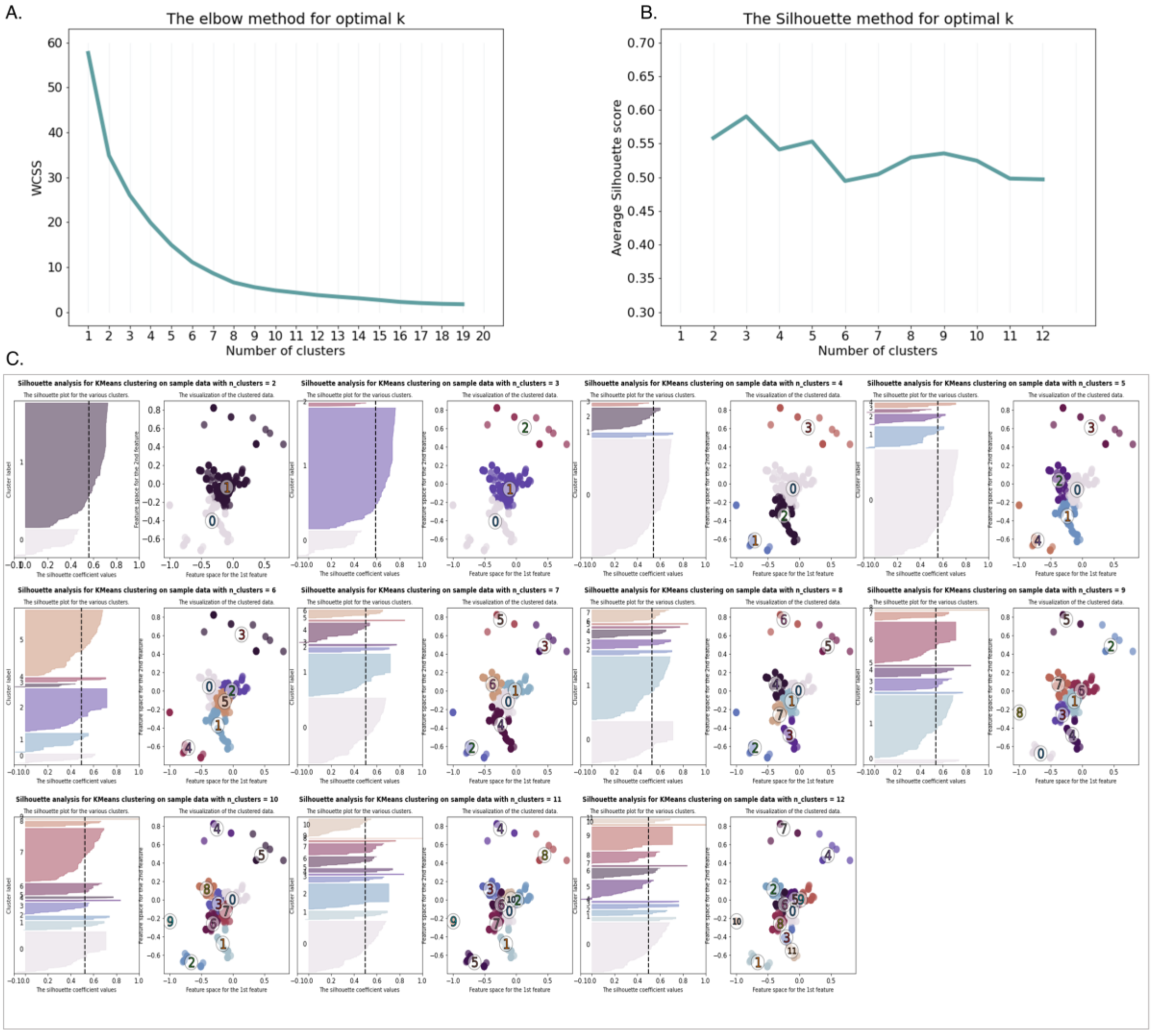

Figure S10. Assessing the quality of k-means clusters fit on the data of GlyPb. A. Elbow method for optimal value of clusters. B. Silhouette score analysis for clustering quality. The Silhouette score was calculated for the whole data set for 2-7 clusters. C. Silhouette analysis for 2-12 clusters.

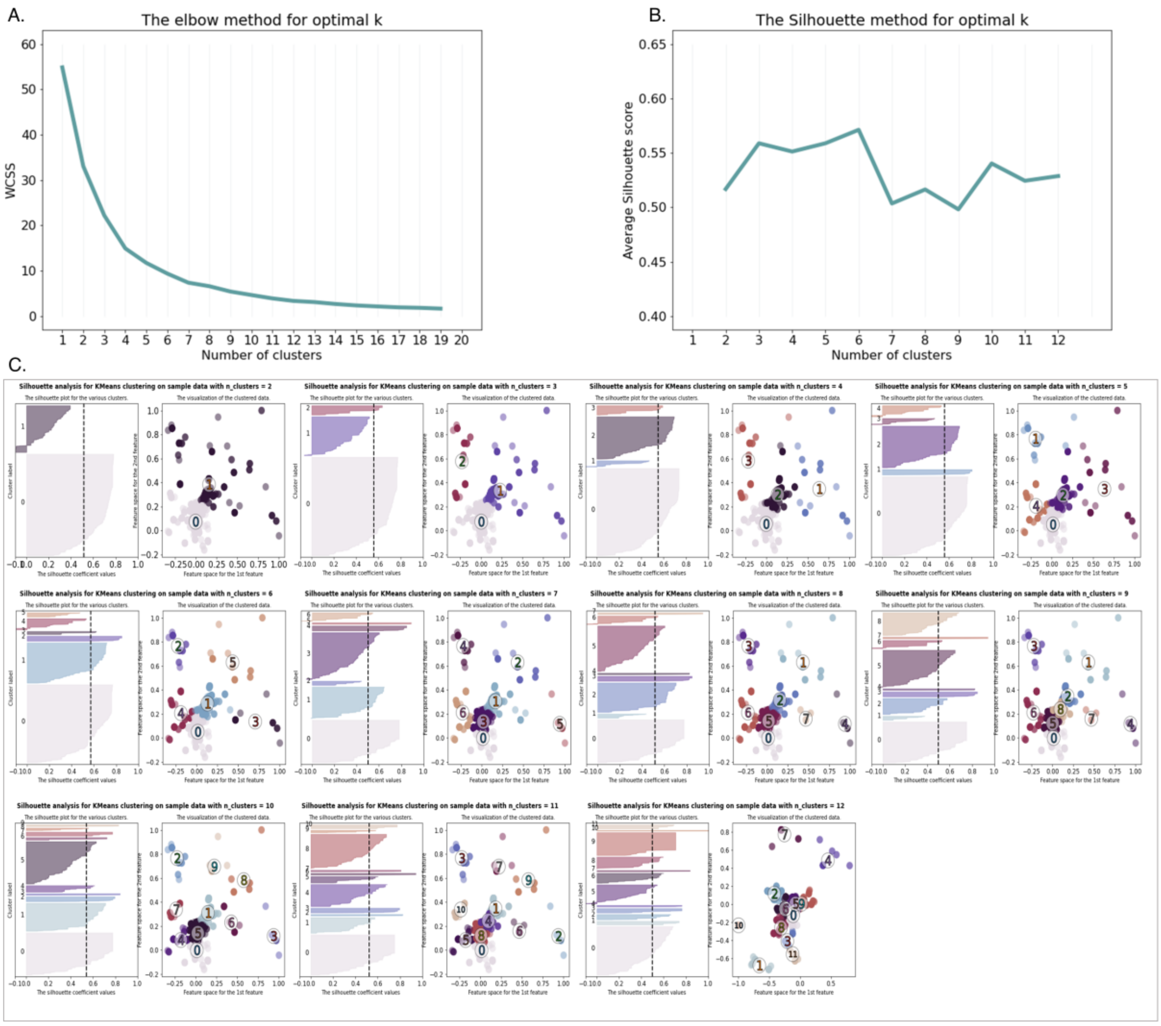

Figure S11. Assessing the quality of k-means clusters fit on the data of GlyPa. A. Elbow method for optimal value of clusters. B. Silhouette score analysis for clustering quality. The Silhouette score was calculated for the whole data set for 2-7 clusters. C. Silhouette analysis for 2-12 clusters.

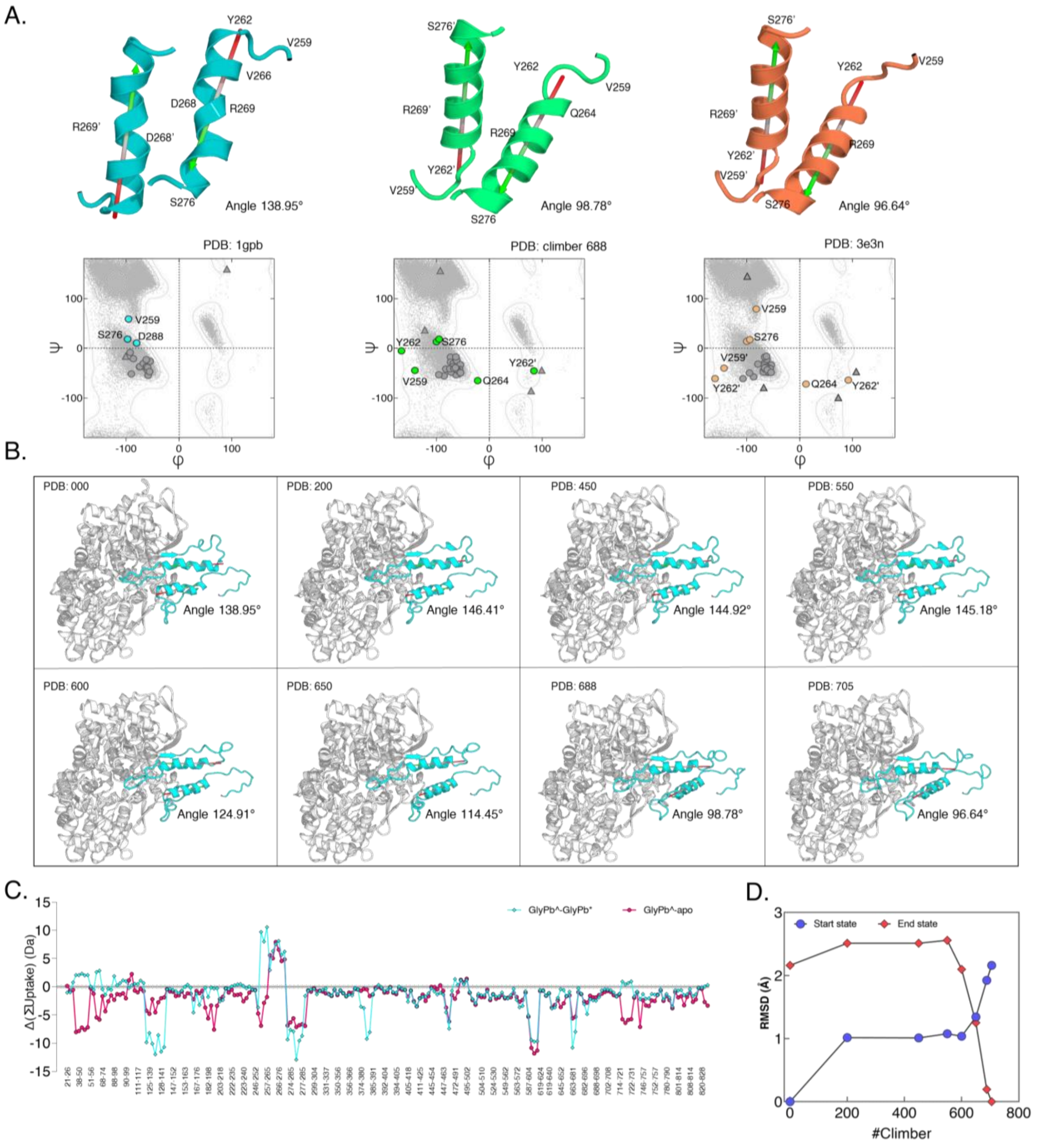

Figure S12. Rotation and transition of the tower helix upon AMP activation of GlyPb. A. Representation of the tower helix dynamics (residues 259-277), from T-state (blue), climber transition (green) and R-state (orange), with calculated angles between the α7 and α7' helices. Ramachandran plots are also included, calculated from the corresponding pdb files, with specific helix outliers highlighted. B. PDBs from the climber transition of GlyPb activation with AMP, including start pdb 000, 200, 450, 550, 600, 650, 688 and end state. The tower helices are colored cyan with the connecting 250 and 280s loops from both dimers. C. Difference plot representing the difference of the sum of uptake between the non-equilibrium GlyPb + AMP/AS data and the equilibrium GlyPb + AMP/AS data (pink), and the non-equilibrium GlyPb + AMP/AS data and GlyPb (cyan). D. The RMSD difference between the start (blue) and end (red) state with the 200, 450, 550, 600, 650, 688 PDBs.

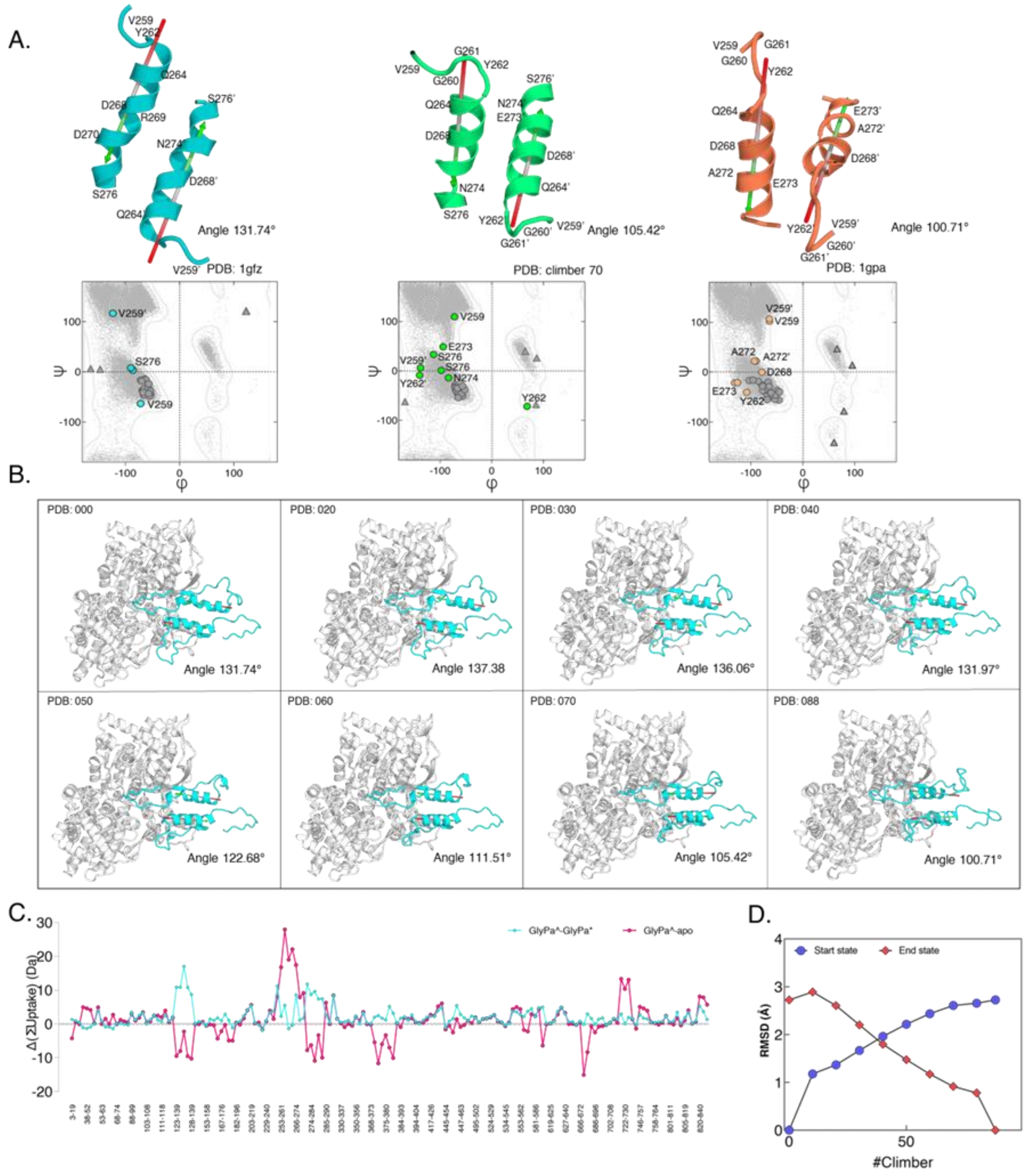

Figure S13. Rotation and transition of the tower helix upon caffeine inhibition of GlyPa. A. Representation of the tower helix dynamics (residues 259-277), from T-state (blue), climber transition (green) and R-state (orange), with calculated angles between the α7 and α7' helices. Ramachandran plots are also included, calculated from the corresponding pdb files, with specific helix outliers highlighted. B. PDBs from the climber transition of GlyPa inhibition with caffeine, including start pdb 000, 020, 030, 040, 050, 060, 070, and end state. The tower helices are colored cyan with the connecting 250 and 280s loops from both dimers. C. Difference plot representing the difference of the sum of uptake between the non-equilibrium GlyPa + caffeine data and the equilibrium GlyPa + caffeine data (pink), and the non-equilibrium GlyPa + caffeine data and GlyPa (cyan). D. The RMSD difference between the start (blue) and end (red) state with the 020, 030, 040, 050, 060, 070 PDBs.

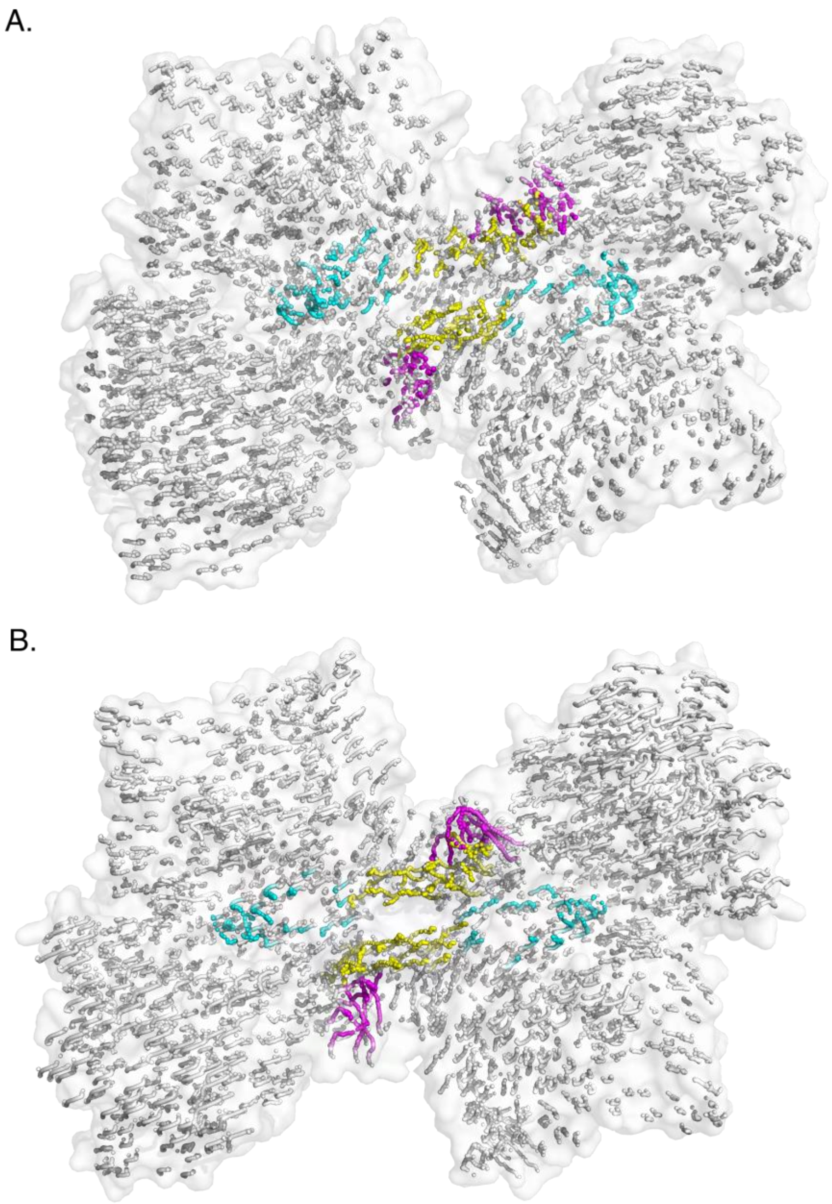

Figure S14. Representation of the full transition upon activation/inhibition. Each backbone nitrogen is represented as a sphere. Colors correspond to specific sections including the tower helix (yellow), 250 loop (pink) and 280 loop (blue). A. The movement of the backbone nitrogens upon AMP/AS activation. B. The movement of the backbone nitrogens upon caffeine inhibition.

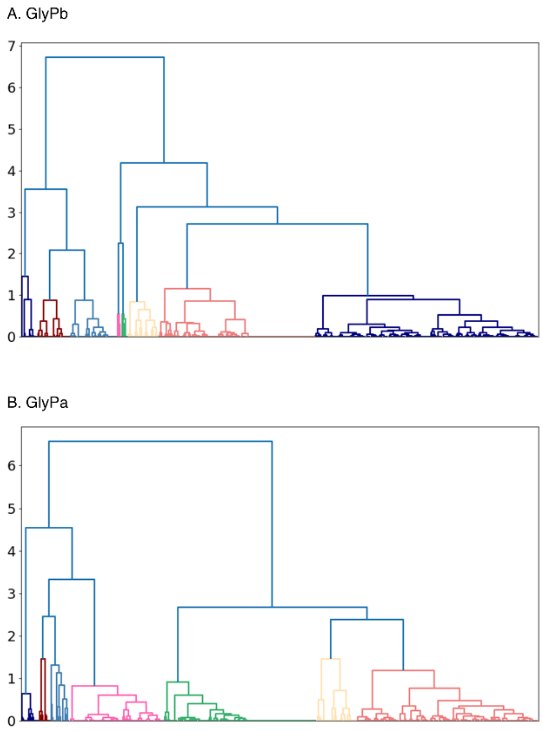

Figure S15. Full dendrogram of hierarchical clustering. The dendrogram was generated with SciPy for all identified peptides monitored by non-equilibrium HDX, where above p=7 in truncate mode, 7 clusters of behaviors were observed. The dendrogram illustrates how each cluster is composed by drawing a U-shaped link between a non-singleton cluster and its children. The top of the U-link indicates a cluster merge. The two legs of the U-link indicate which clusters were merged. The length of the two legs of the U-link represents the distance between the child clusters. It is also the cophenetic distance between original observations in the two children clusters.

Figure S16. HDX uptake curves of GlyPb per peptide. Absolute data for three protein states shown (blue – apo GlyPb; orange – AMP-AS bound activated GlyPb; green – AMP-AS bound non-equilibrium activated GlyPb). Peptide sequence and amino acid number given above the plot. Y-axis shows absolute % deuteration at labeling time (ms) on X-axis.

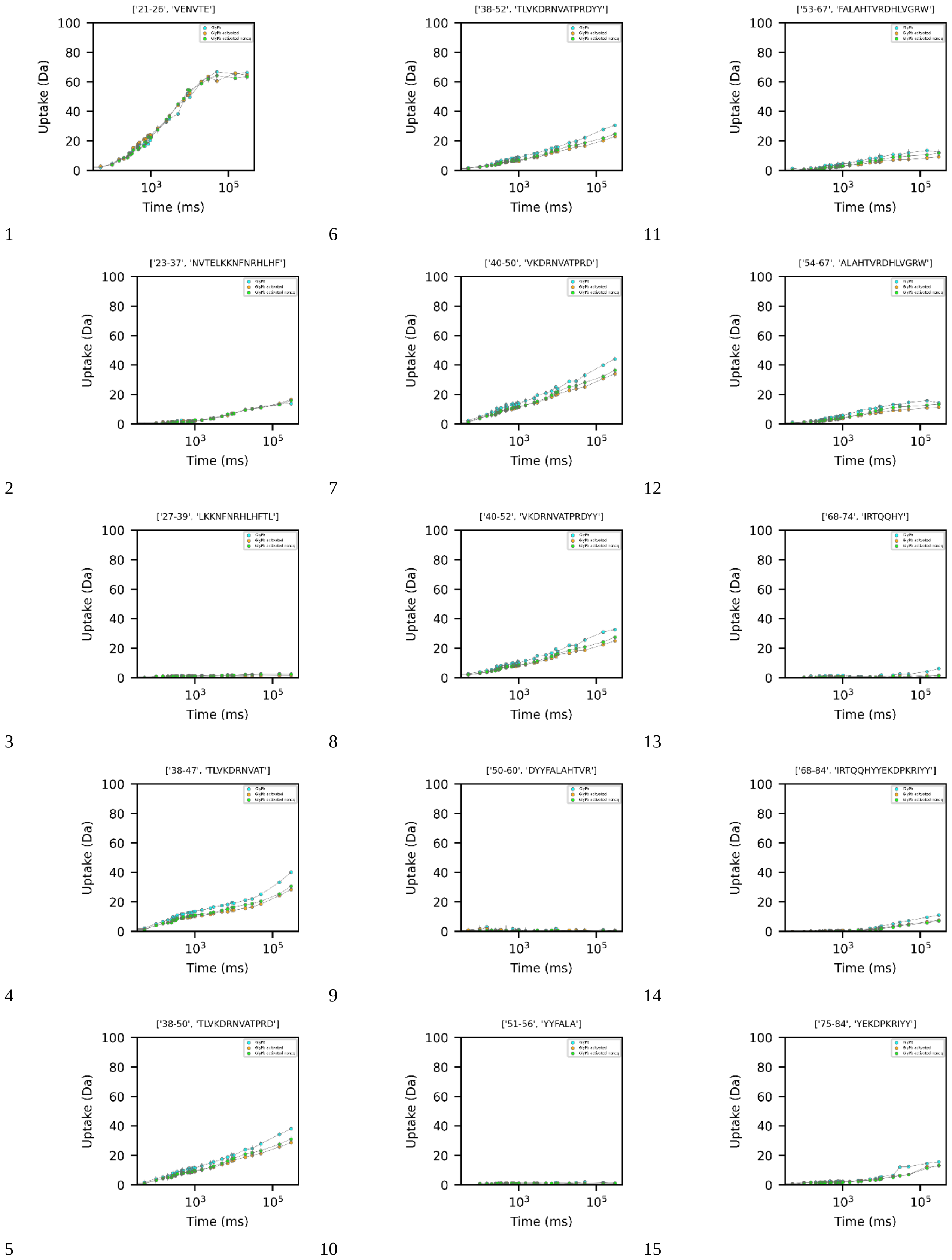

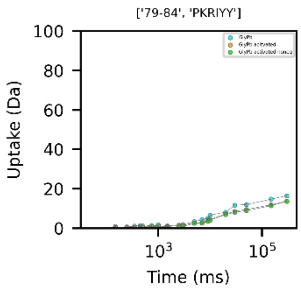

16

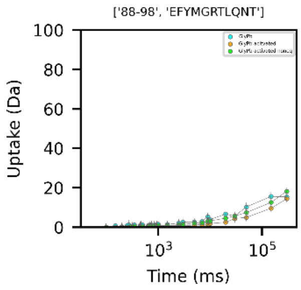

17

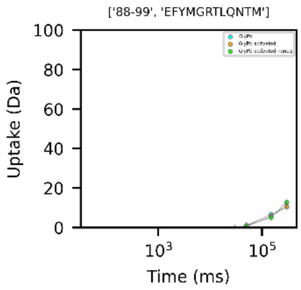

18

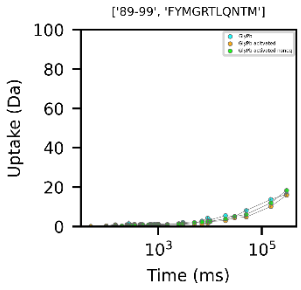

19

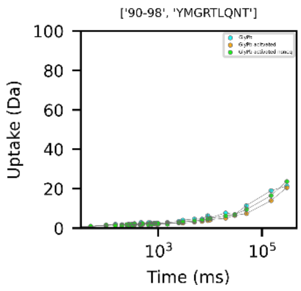

20

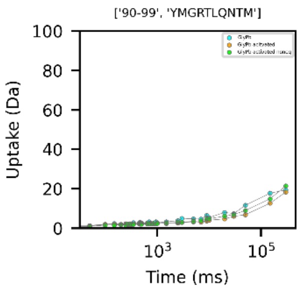

21

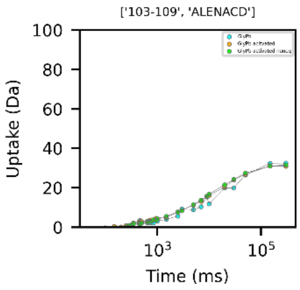

22

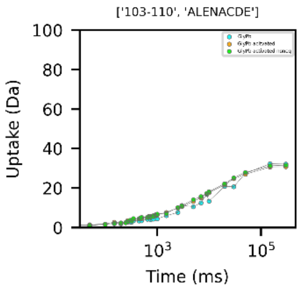

23

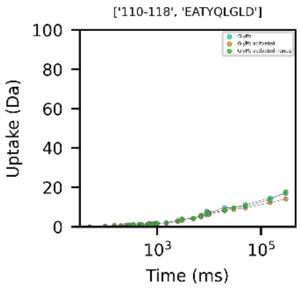

24

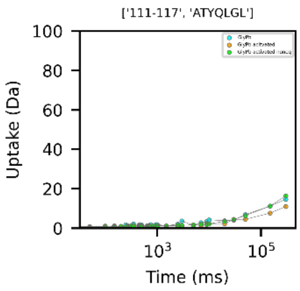

25

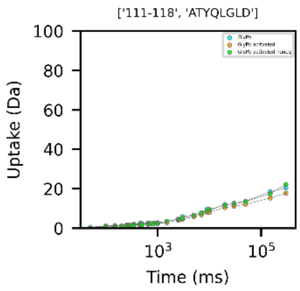

26

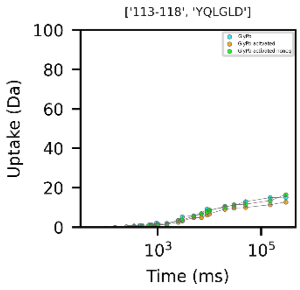

27

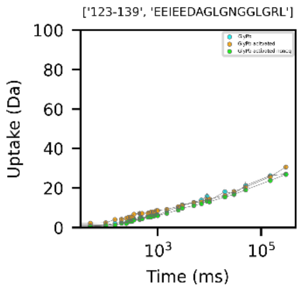

28

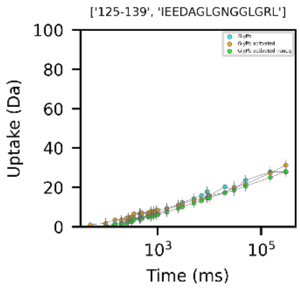

29

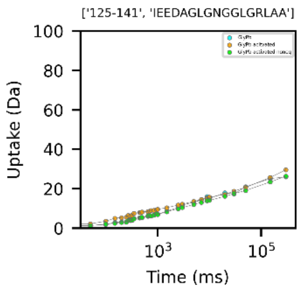

30

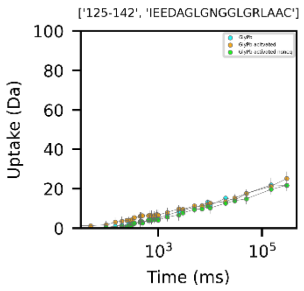

31

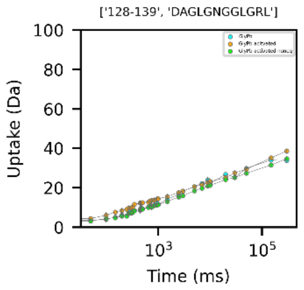

32

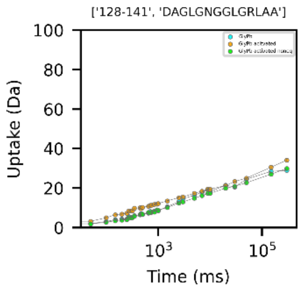

33

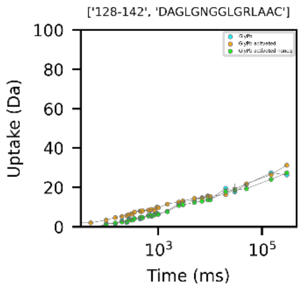

34

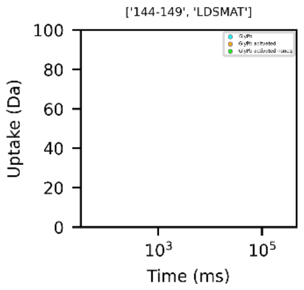

35

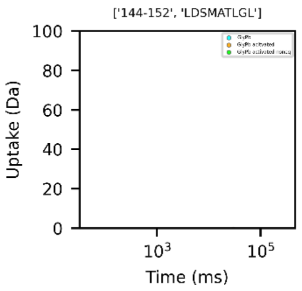

36

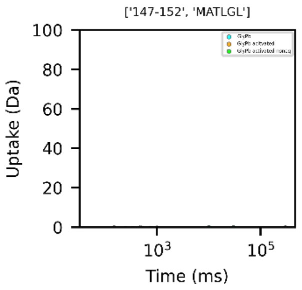

37

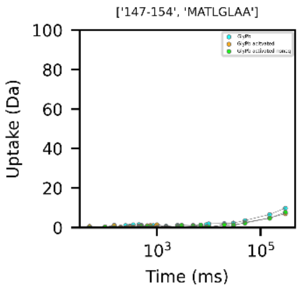

38

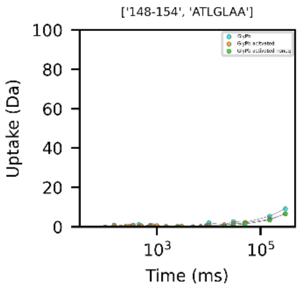

39

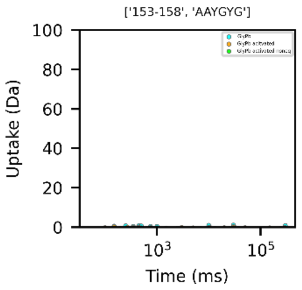

40

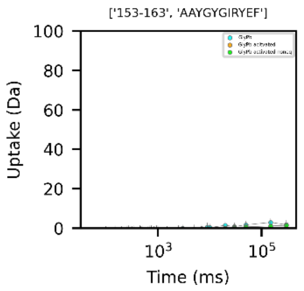

41

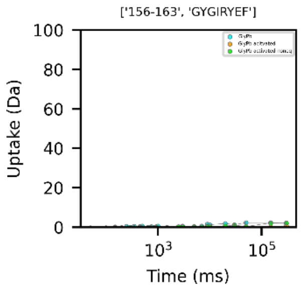

42

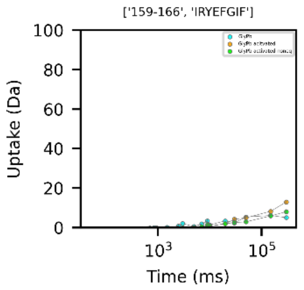

43

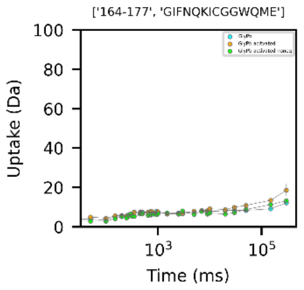

44

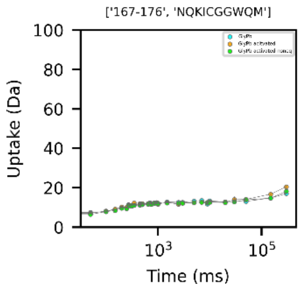

45

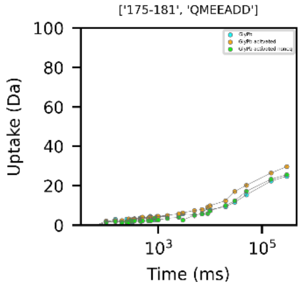

46

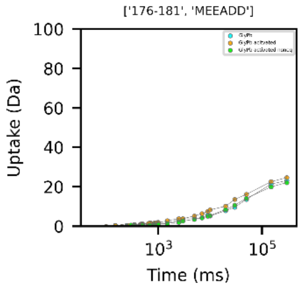

47

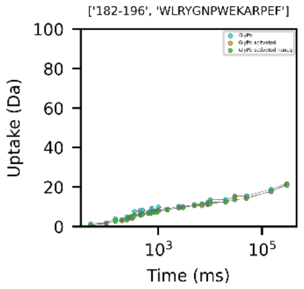

48

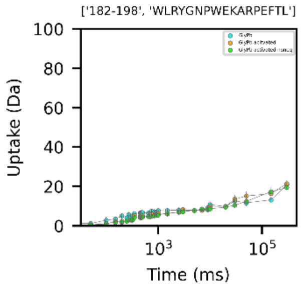

49

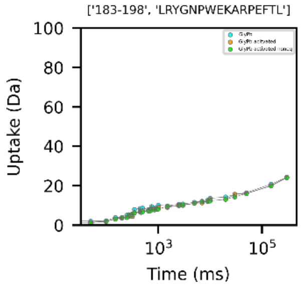

50

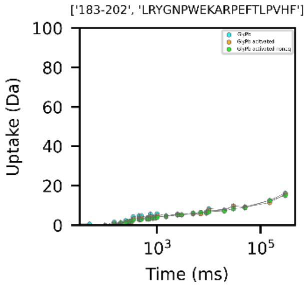

51

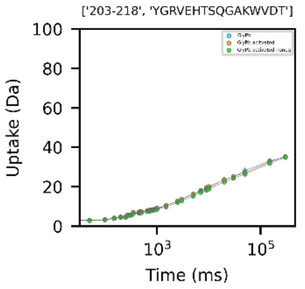

52

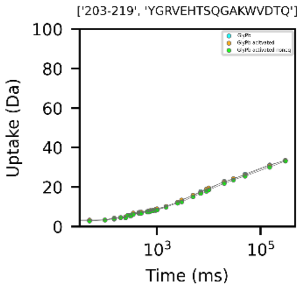

53

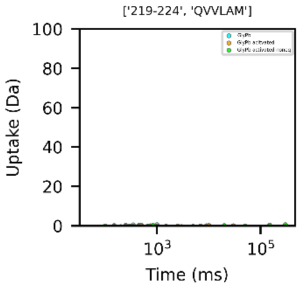

54

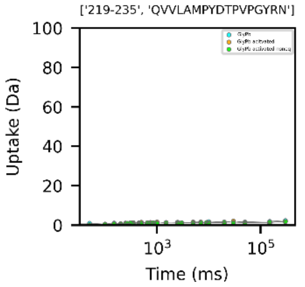

55

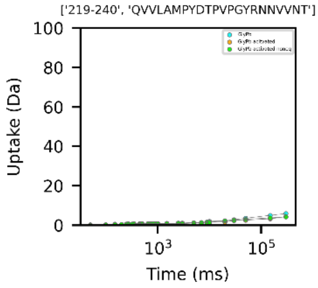

56

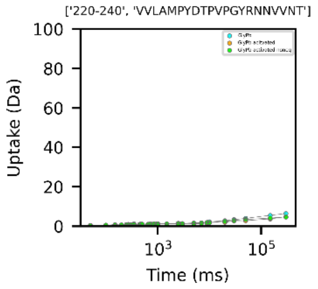

57

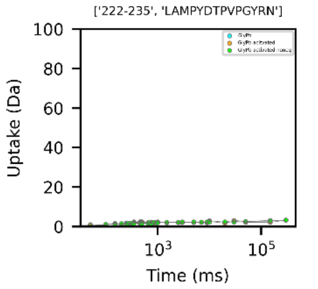

58

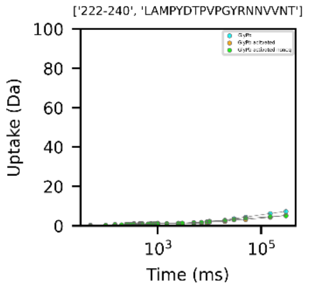

59

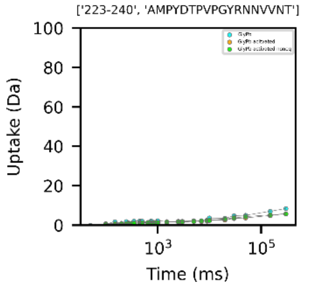

60

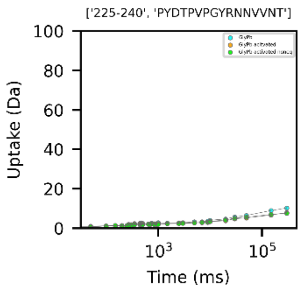

61

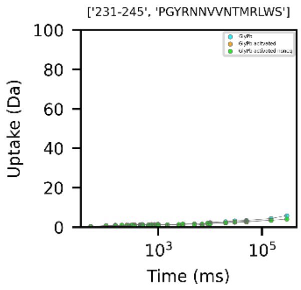

62

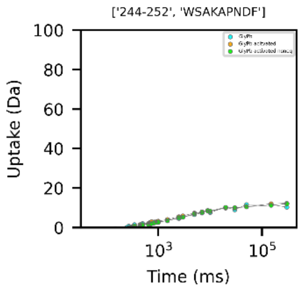

63

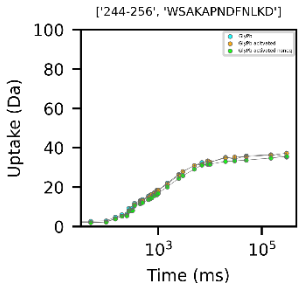

64

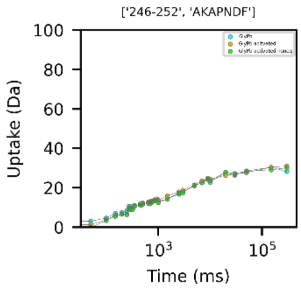

65

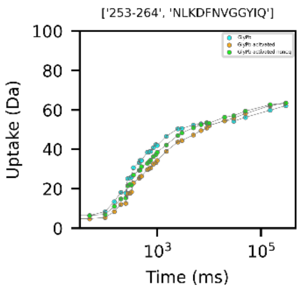

66

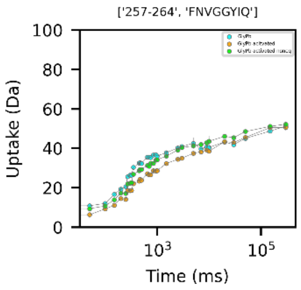

67

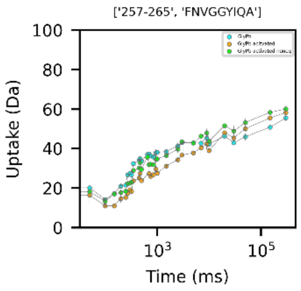

68

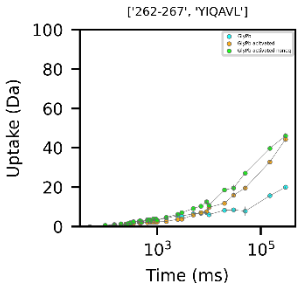

69

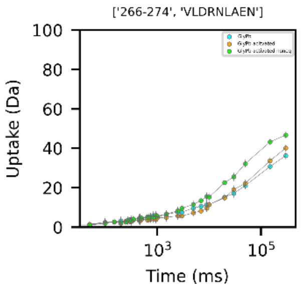

70

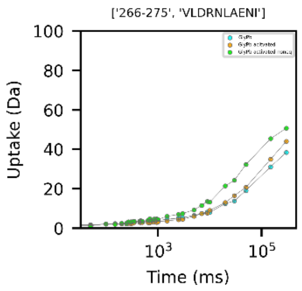

71

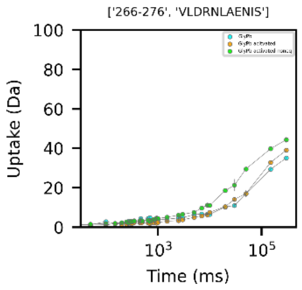

72

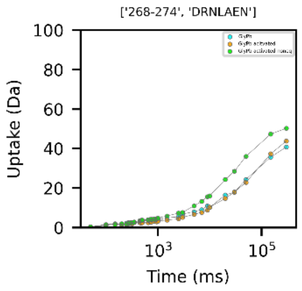

73

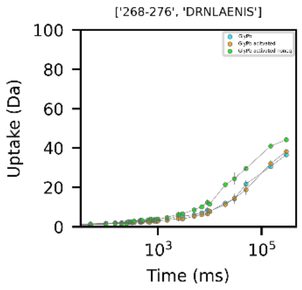

74

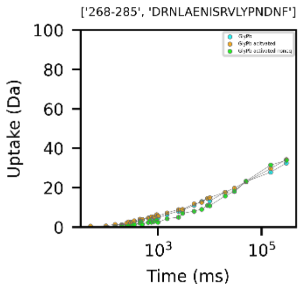

75

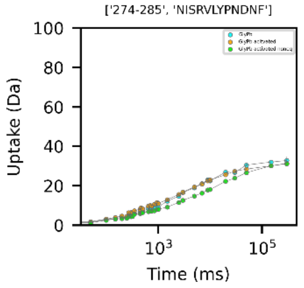

76

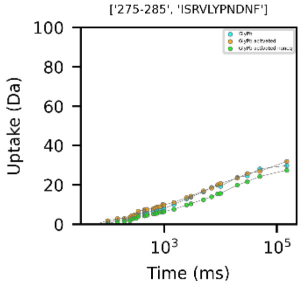

77

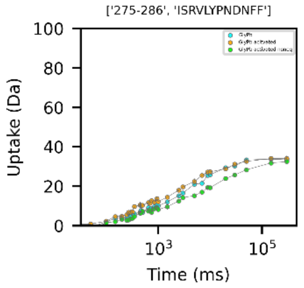

78

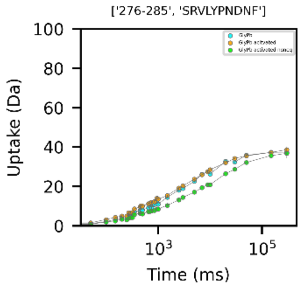

79

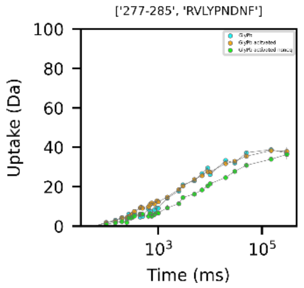

80

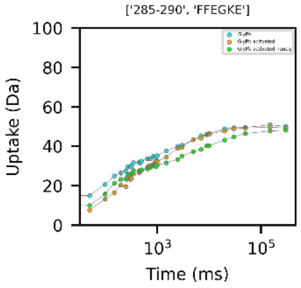

81

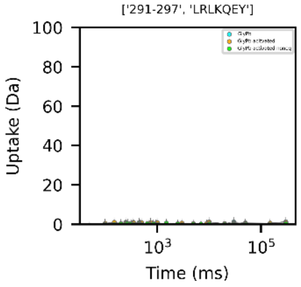

82

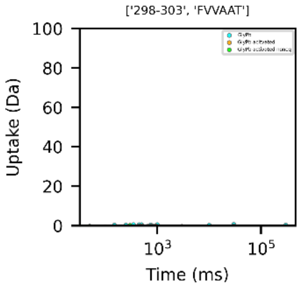

83

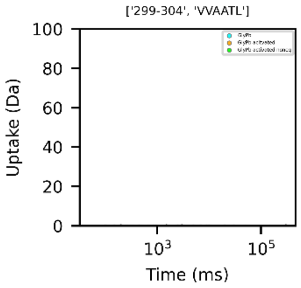

84

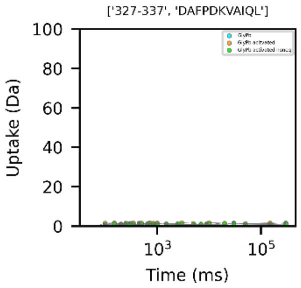

85

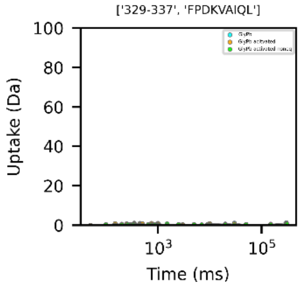

86

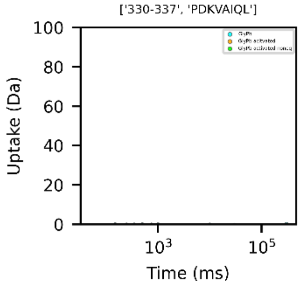

87

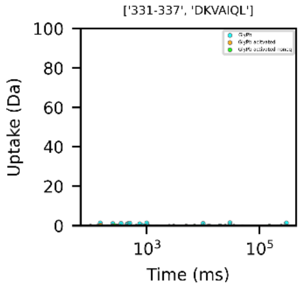

88

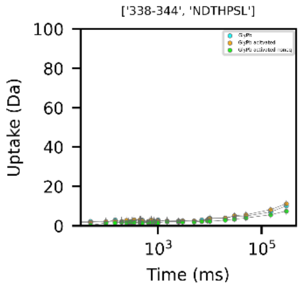

89

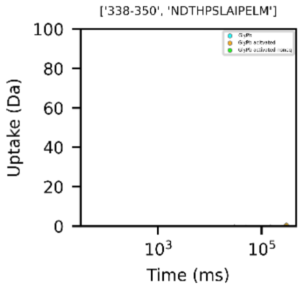

90

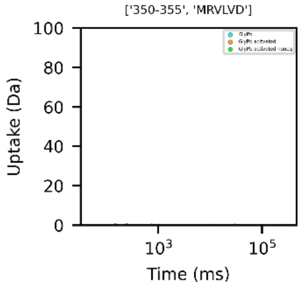

91

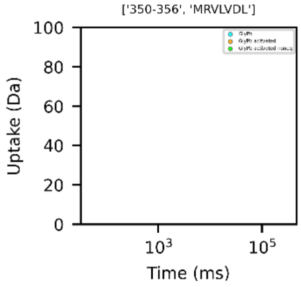

92

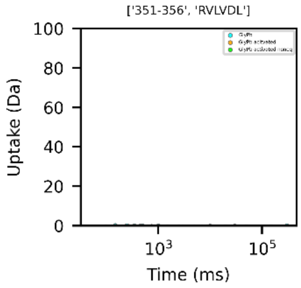

93

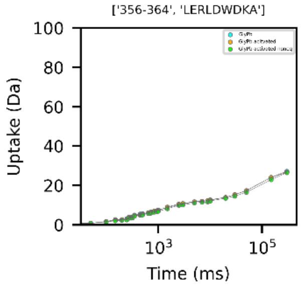

94

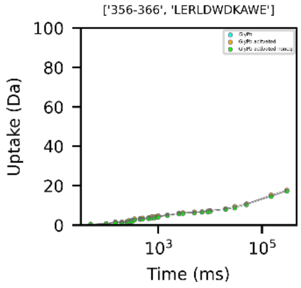

95

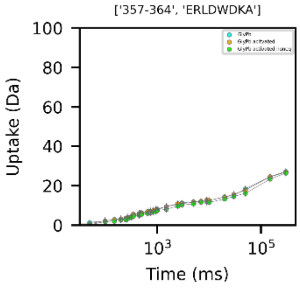

96

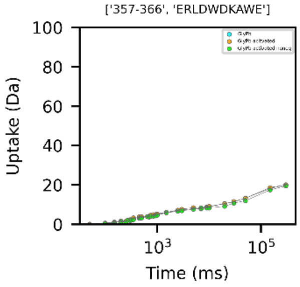

97

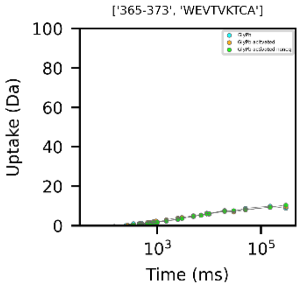

98

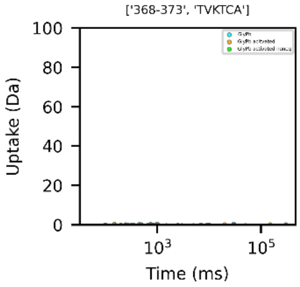

99

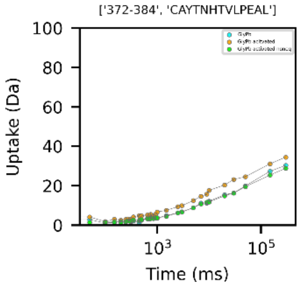

100

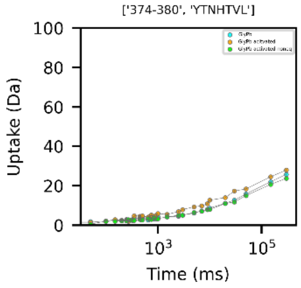

101

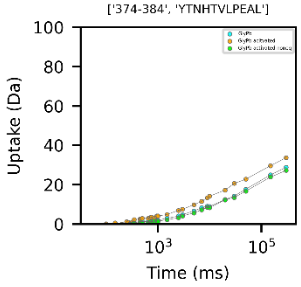

102

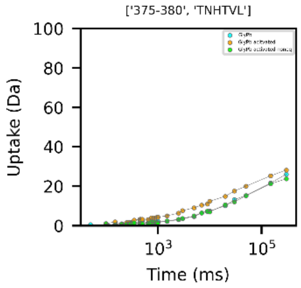

103

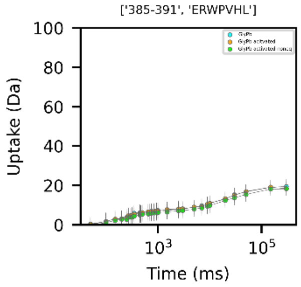

104

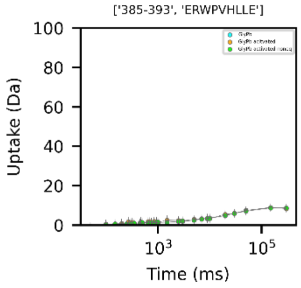

105

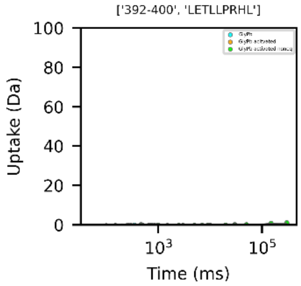

106

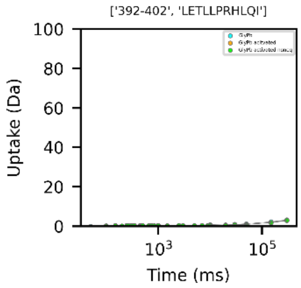

107

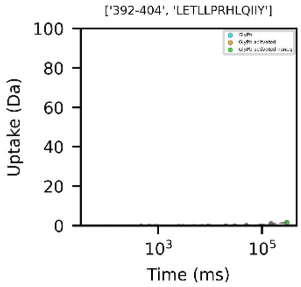

108

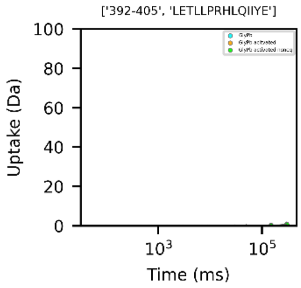

109

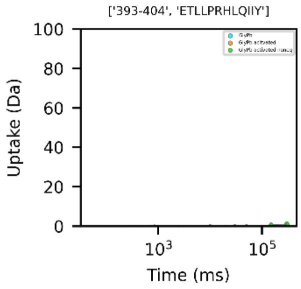

110

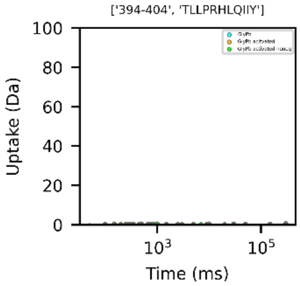

111

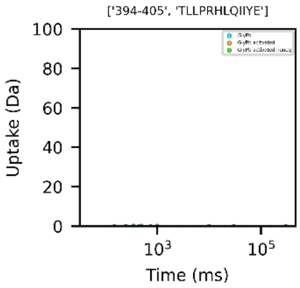

112

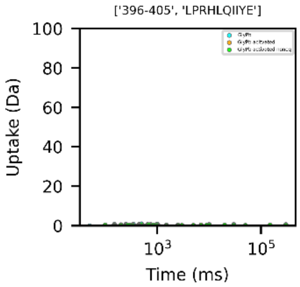

113

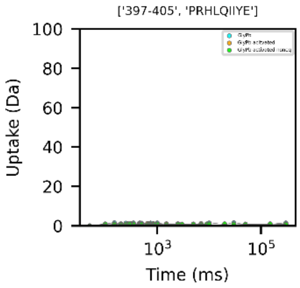

114

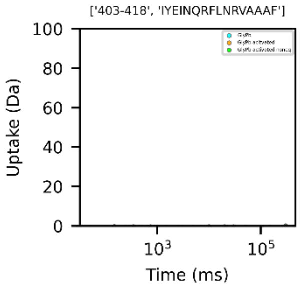

115

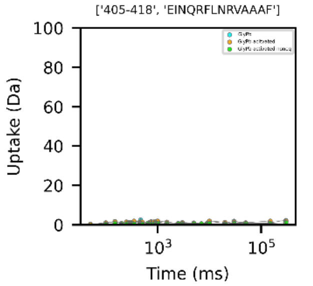

116

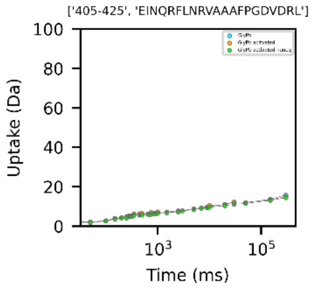

117

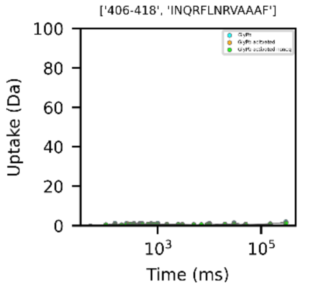

118

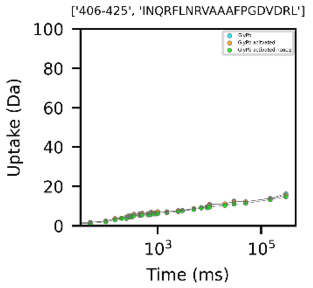

119

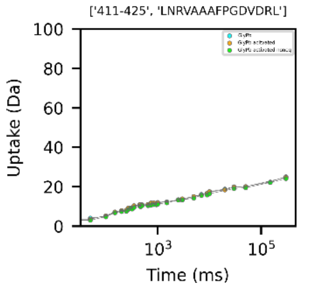

120

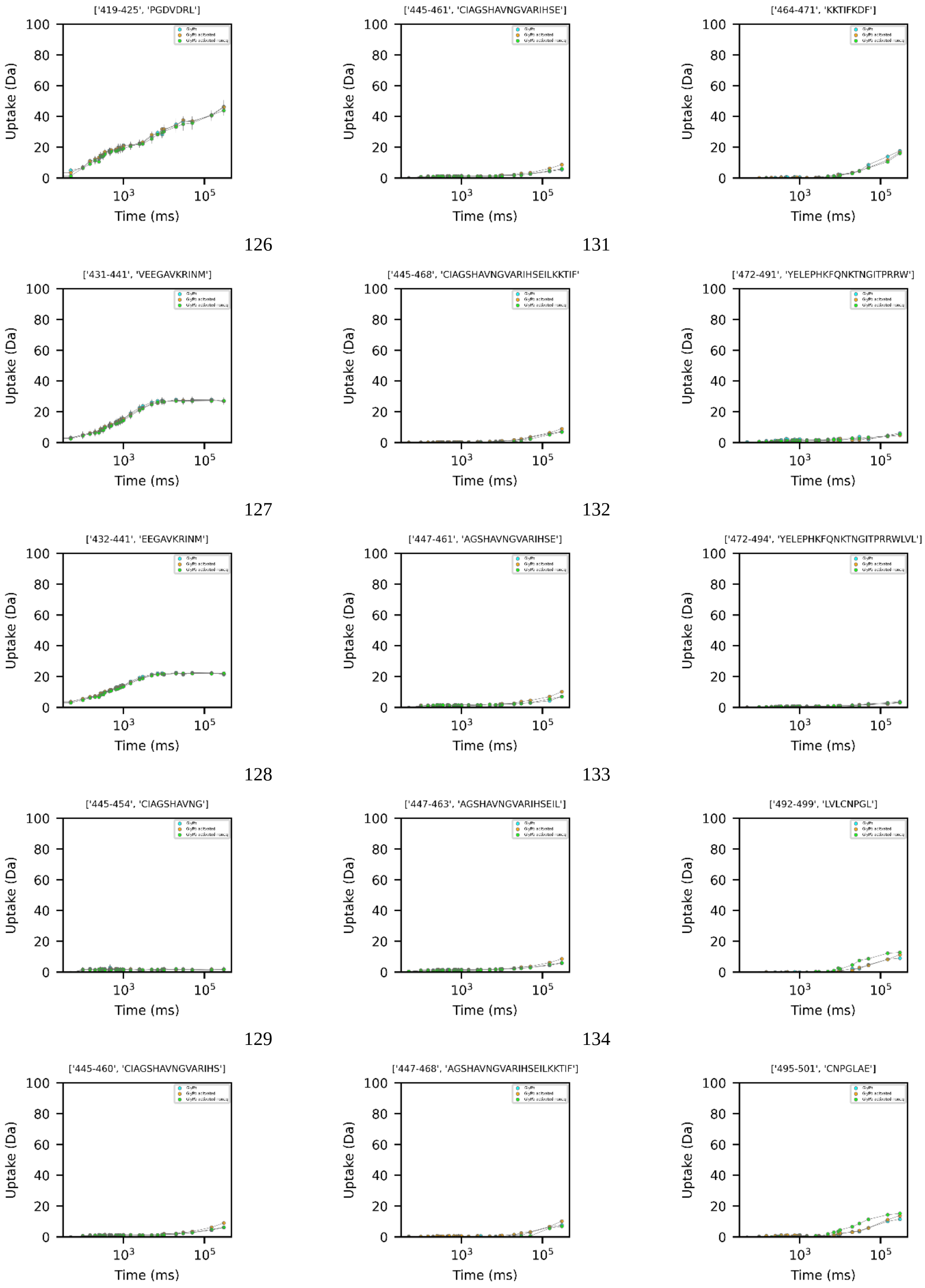

121
122
123
124
125
126
127
128
129
130
131
132
133
134
135

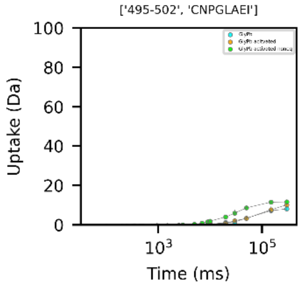

136

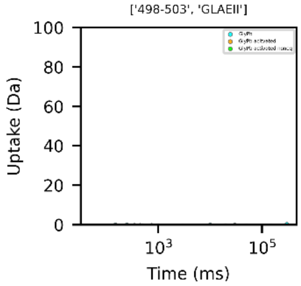

137

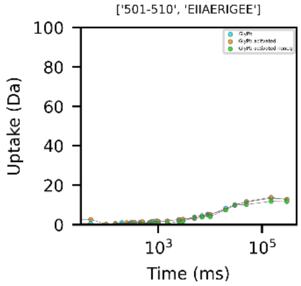

138

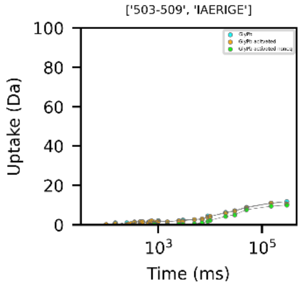

139

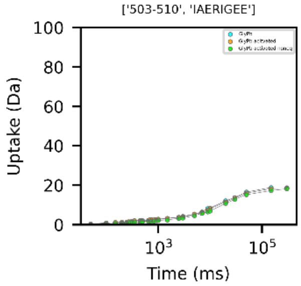

140

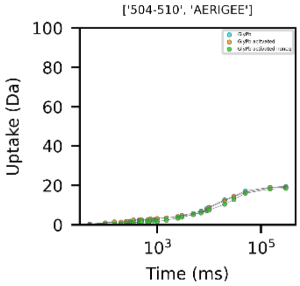

141

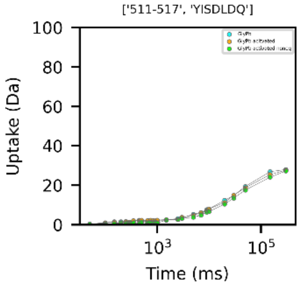

142

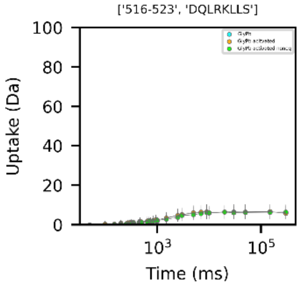

143

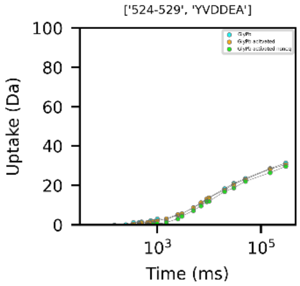

144

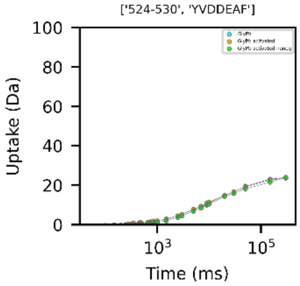

145

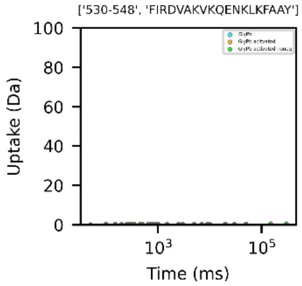

146

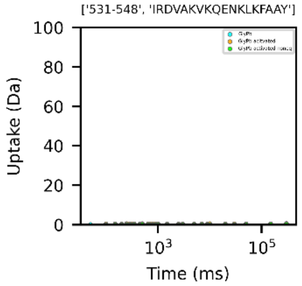

147

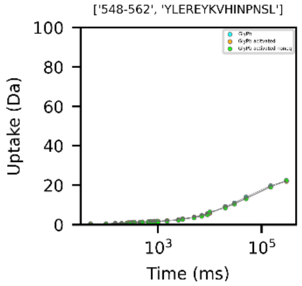

148

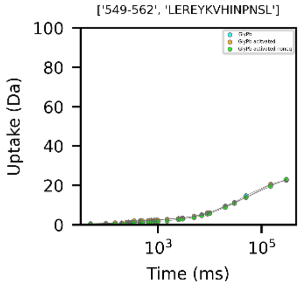

149

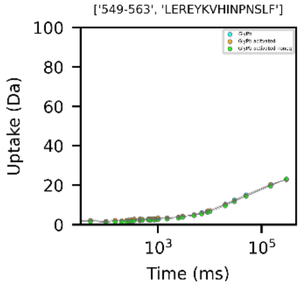

150

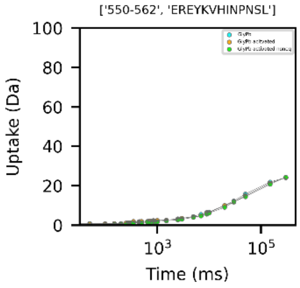

151

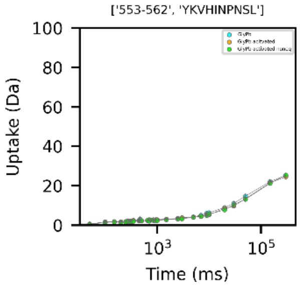

152

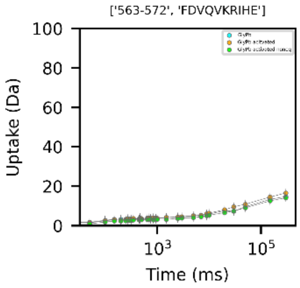

153

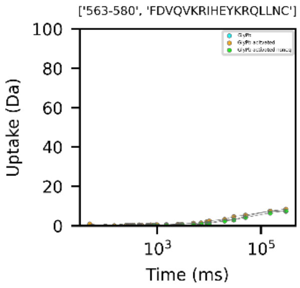

154

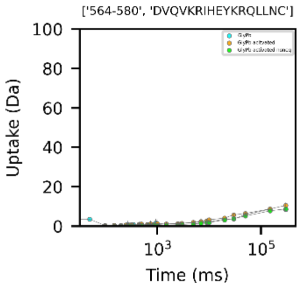

155

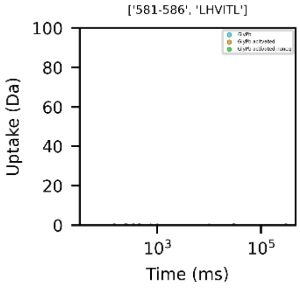

156

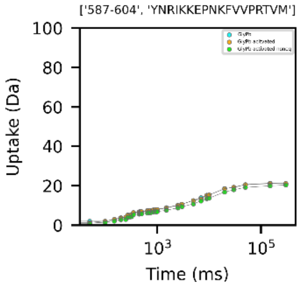

157

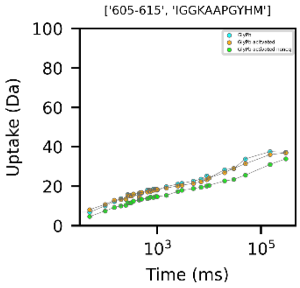

158

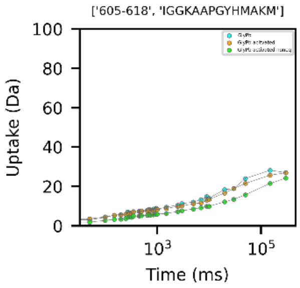

159

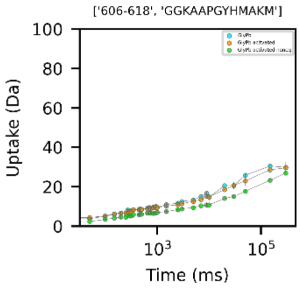

160

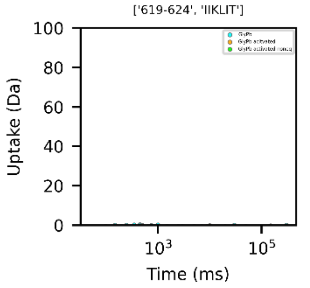

161

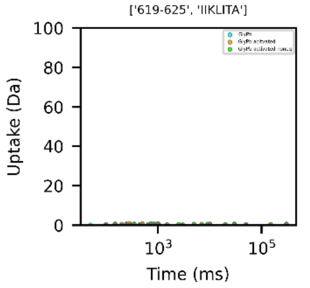

162

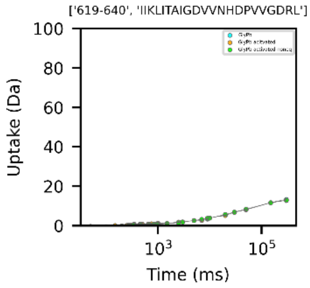

163

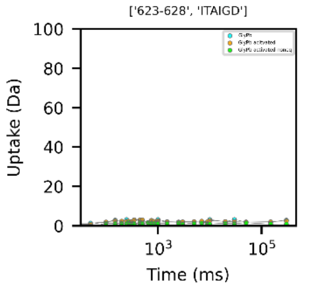

164

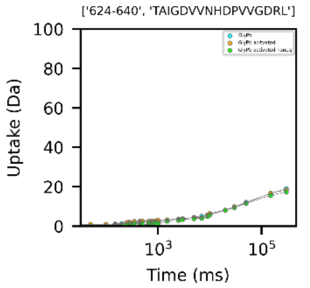

165

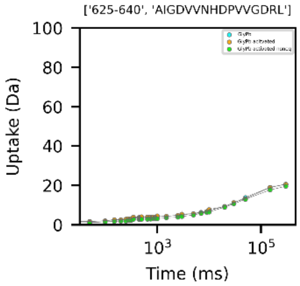

166

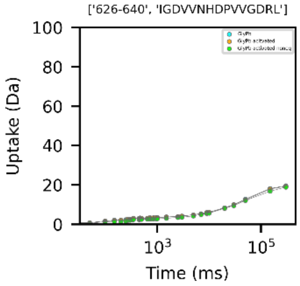

167

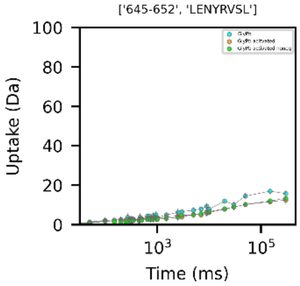

168

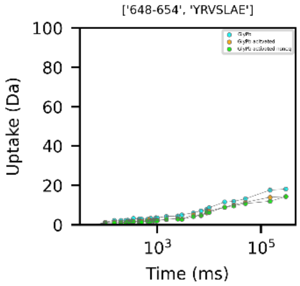

169

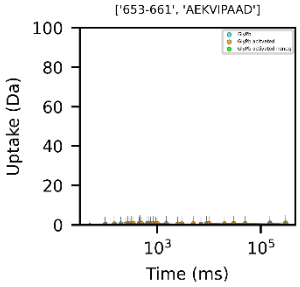

170

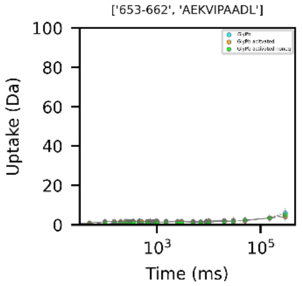

171

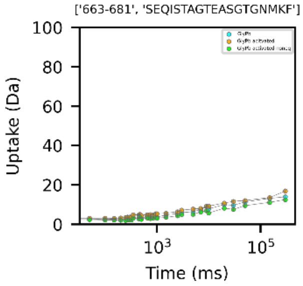

172

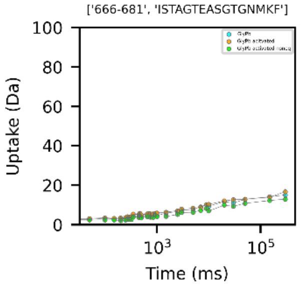

173

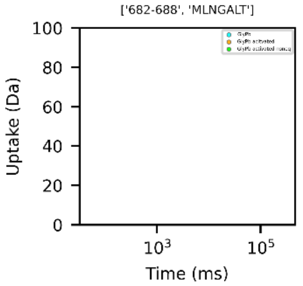

174

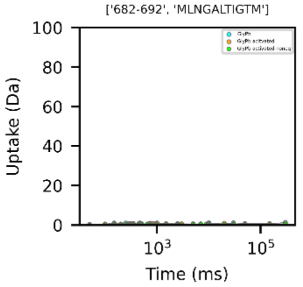

175

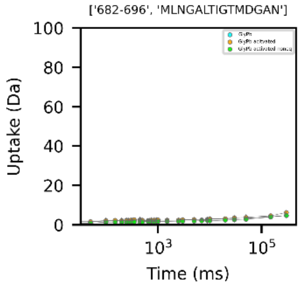

176

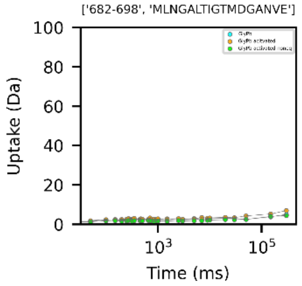

177

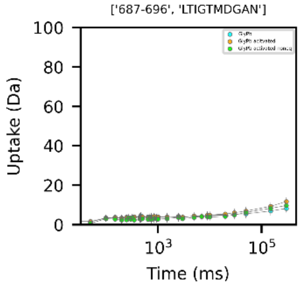

178

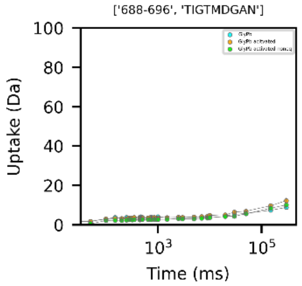

179

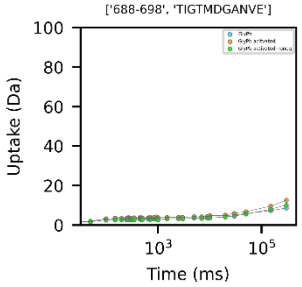

180

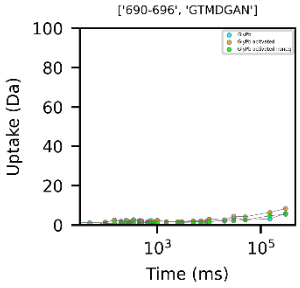

181

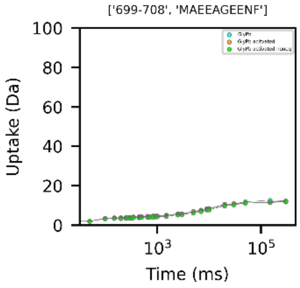

182

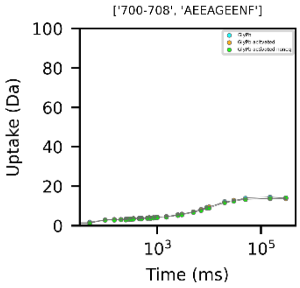

183

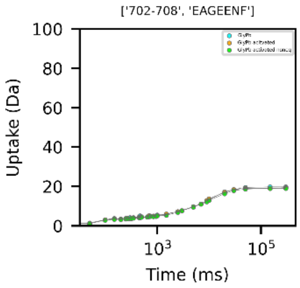

184

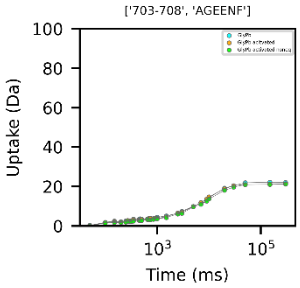

185

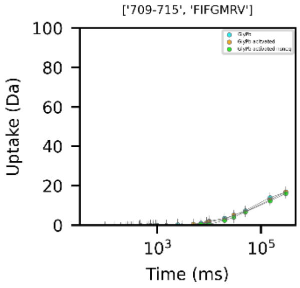

186

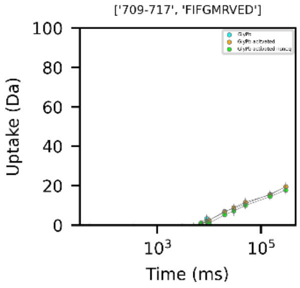

187

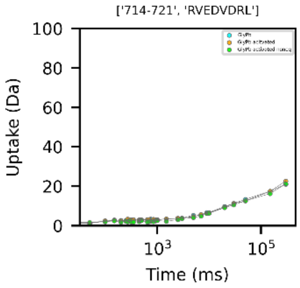

188

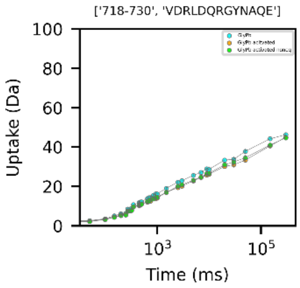

189

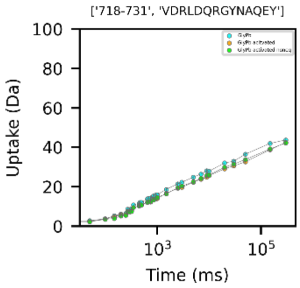

190

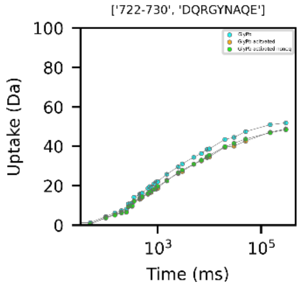

191

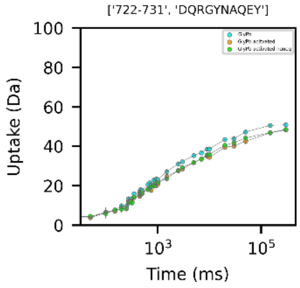

192

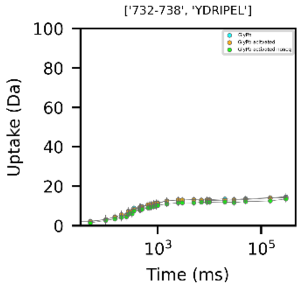

193

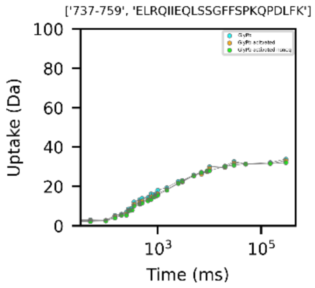

194

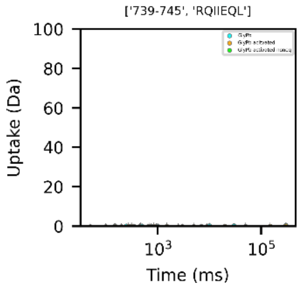

195

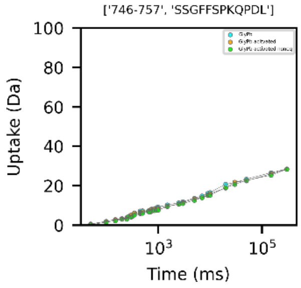

196

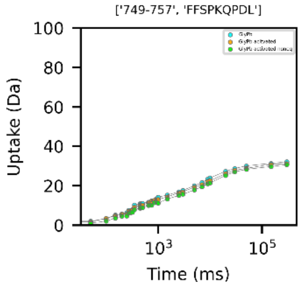

197

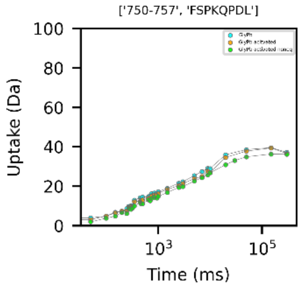

198

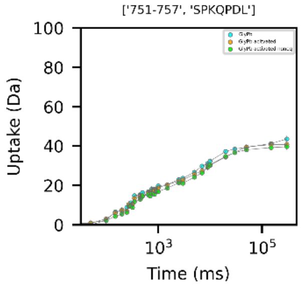

199

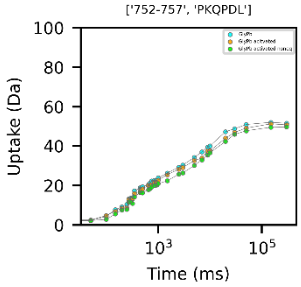

200

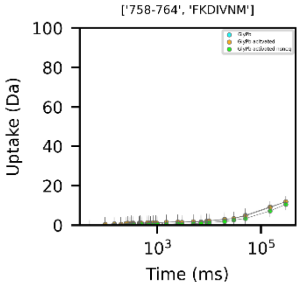

201

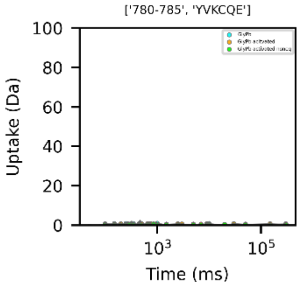

202

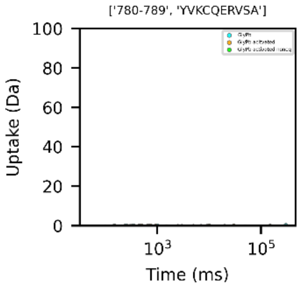

203

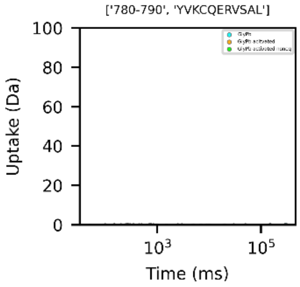

204

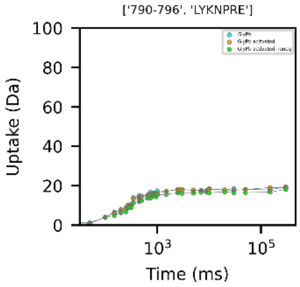

205

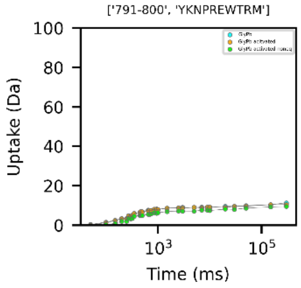

206

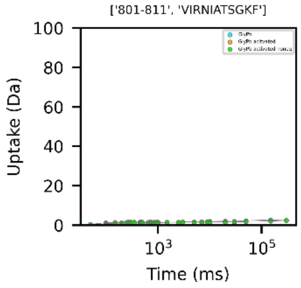

207

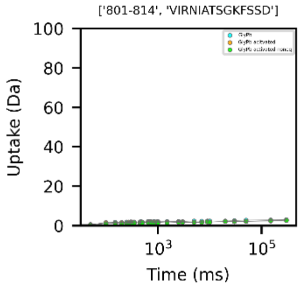

208

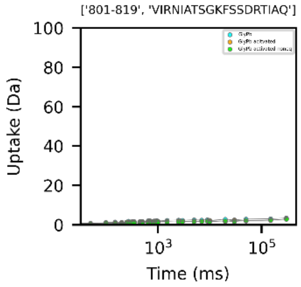

209

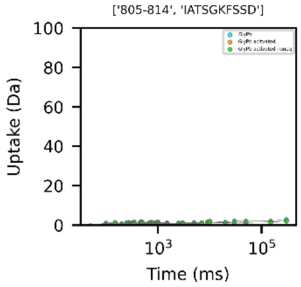

210

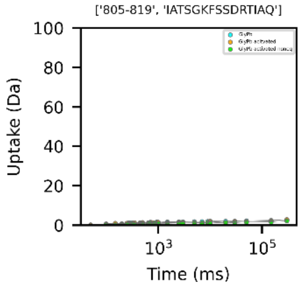
['805-819', 'IATSGKFSSDRTIAQ']
Uptake (Da)
Time (ms)

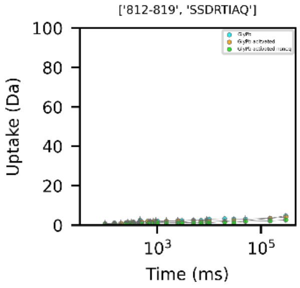
['812-819', 'SSDRTIAQ']
Uptake (Da)
Time (ms)

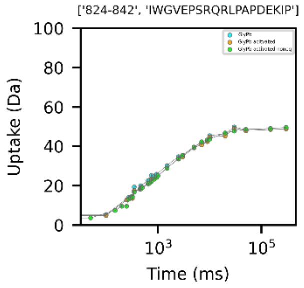
['824-842', 'IWGVEPSRQRLPAPDEKIP']
Uptake (Da)
Time (ms)

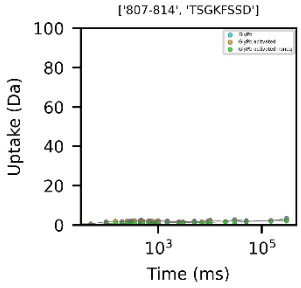
['807-814', 'TSGKFSSD']
Uptake (Da)
Time (ms)

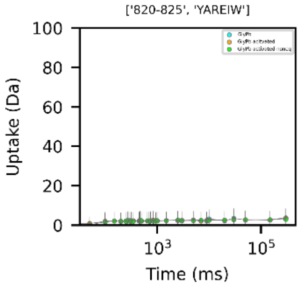
['820-825', 'YAREIW']
Uptake (Da)
Time (ms)

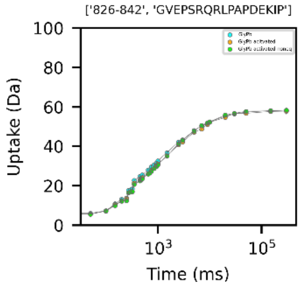
['826-842', 'GVEPSRQRLPAPDEKIP']
Uptake (Da)
Time (ms)

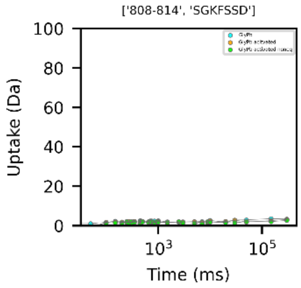
['808-814', 'SGKFSSD']
Uptake (Da)
Time (ms)

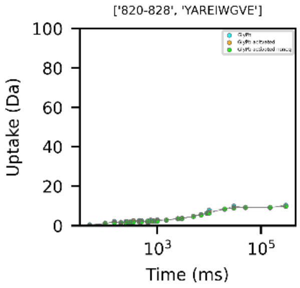
['820-828', 'YAREIWGVE']
Uptake (Da)
Time (ms)

Figure S17. HDX uptake curves of GlyPa per peptide. Absolute data for three protein states shown (blue – apo GlyPa; orange – caffeine bound activated GlyPa; green – caffeine bound non-equilibrium activated GlyPa). Peptide sequence and amino acid number given above the plot. Y-axis shows absolute % deuteration at labeling time (ms) on X-axis.

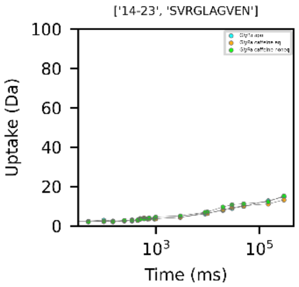

1

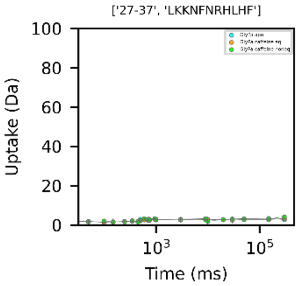

2

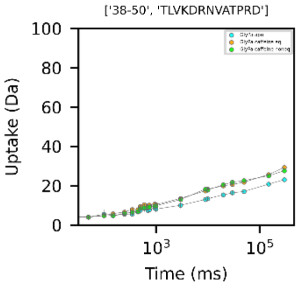

3

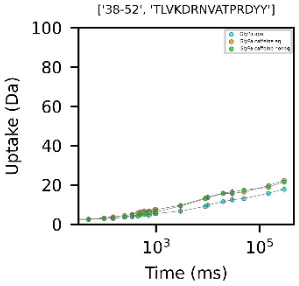

4

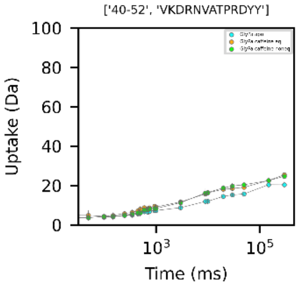

5

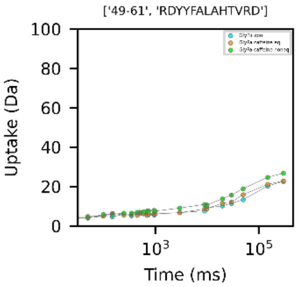

6

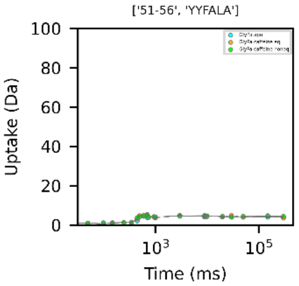

7

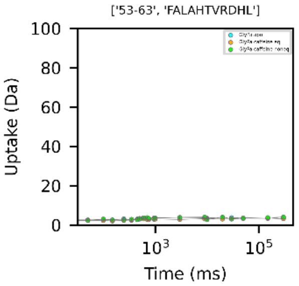

8

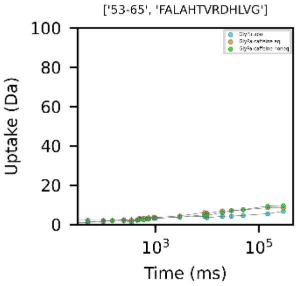

9

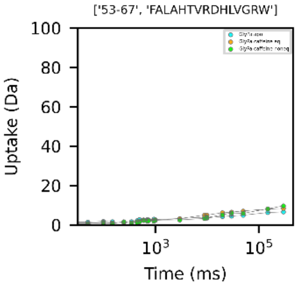

10

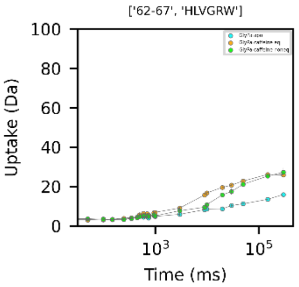

11

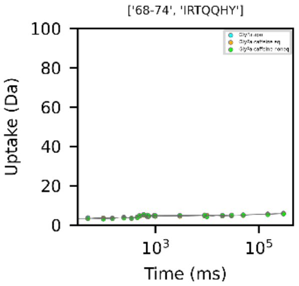

12

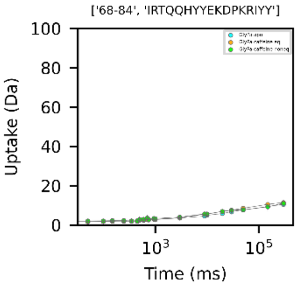

13

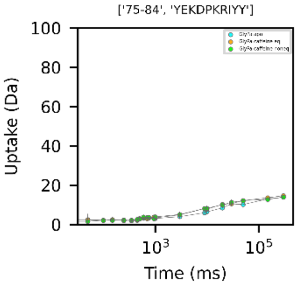

14

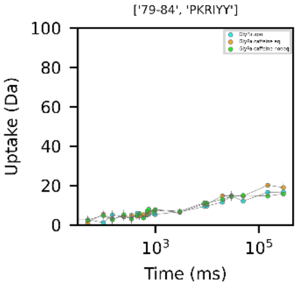

15

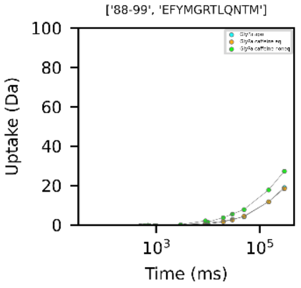

16

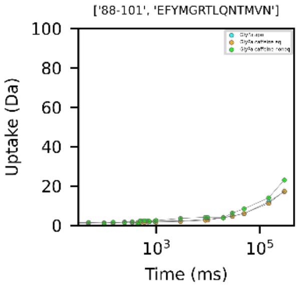

17

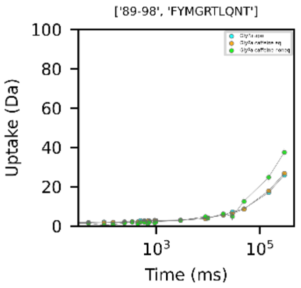

18

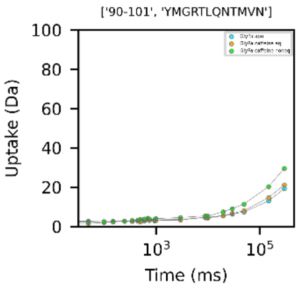

19

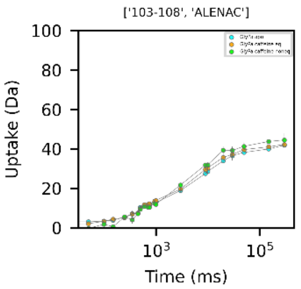

20

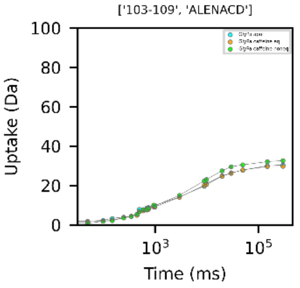

21

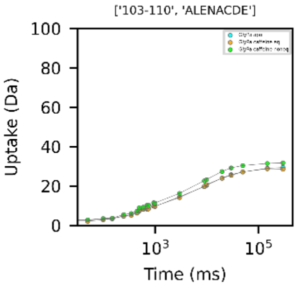

22

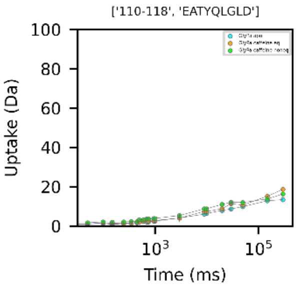

23

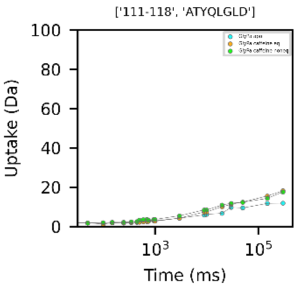

24

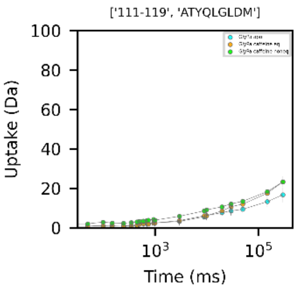

25

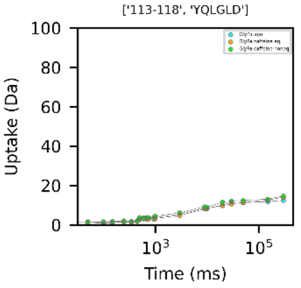

26

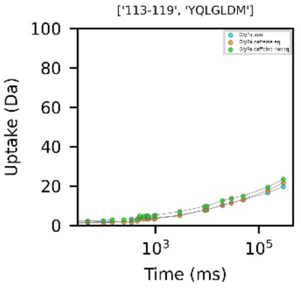

27

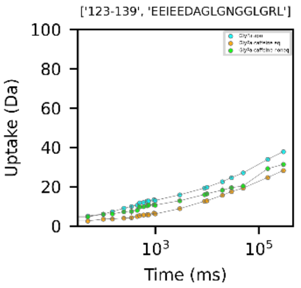

28

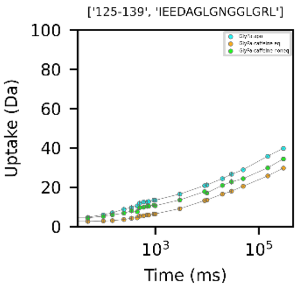

29

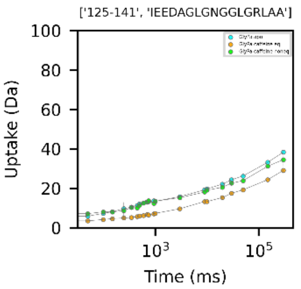

30

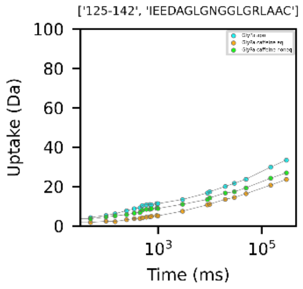

31

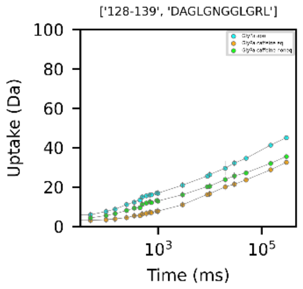

32

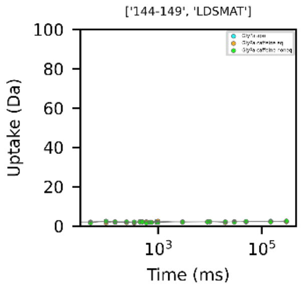

33

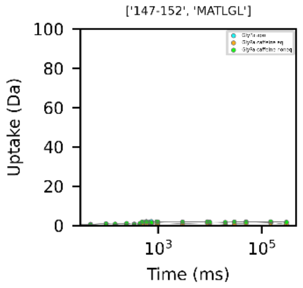

34

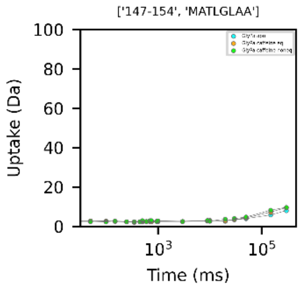

35

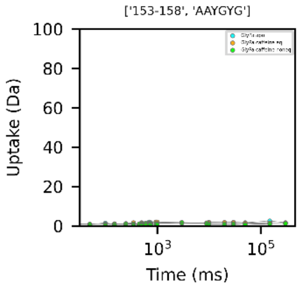

36

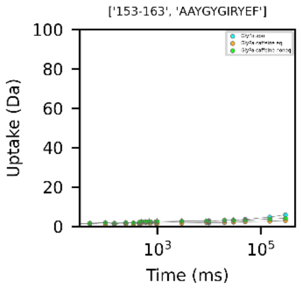

37

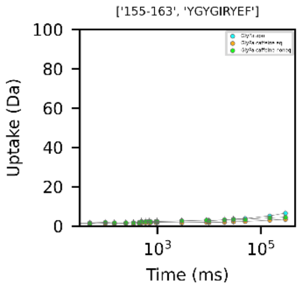

38

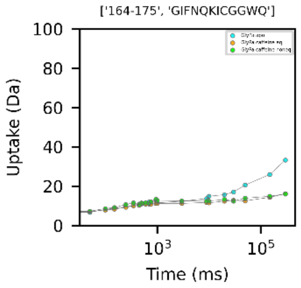

39

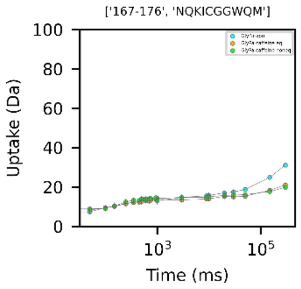

40

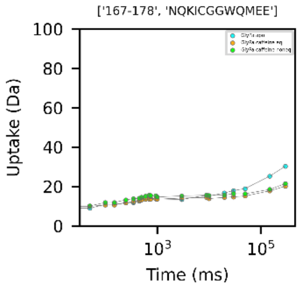

41

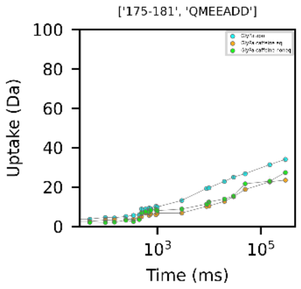

42

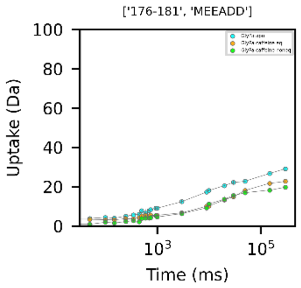

43

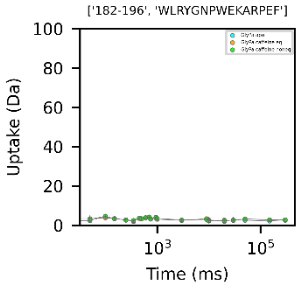

44

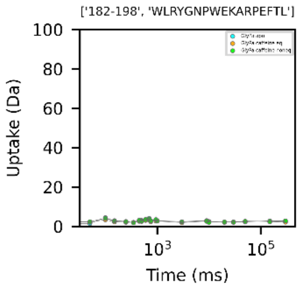

45

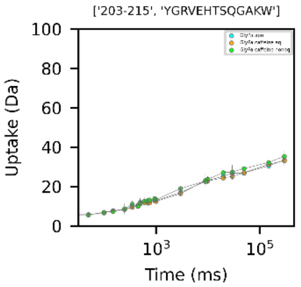

46

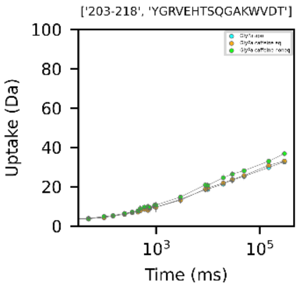

47

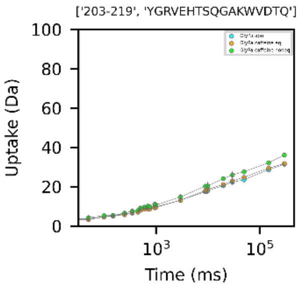

48

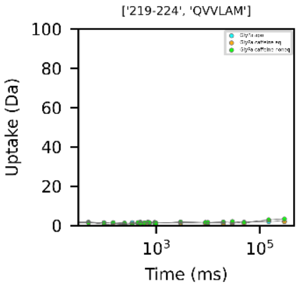

49

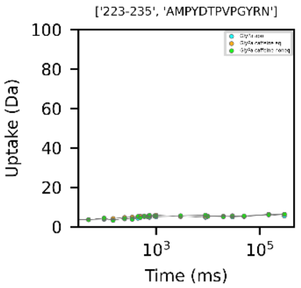

50

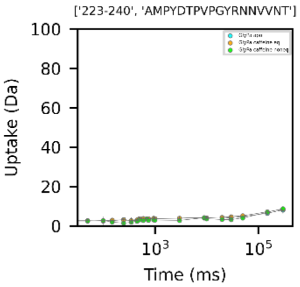

51

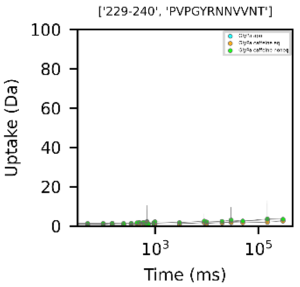

52

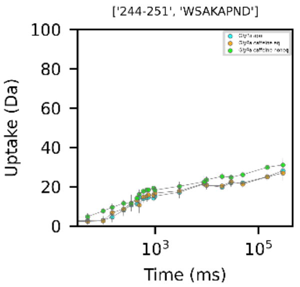

53

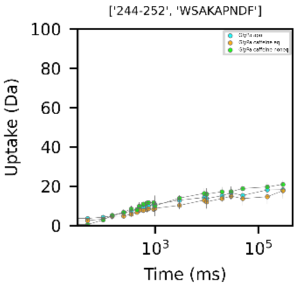

54

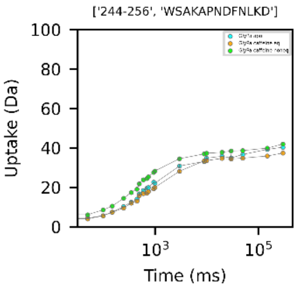

55

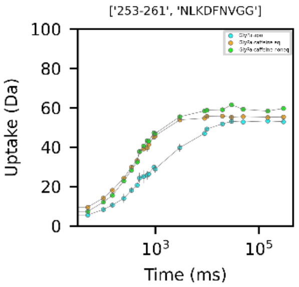

56

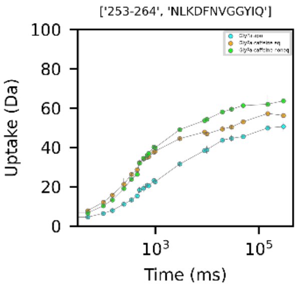

57

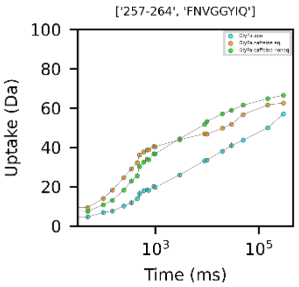

58

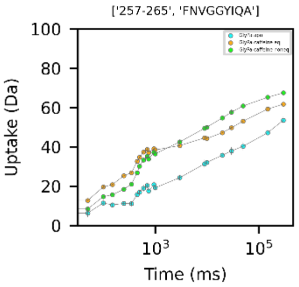

59

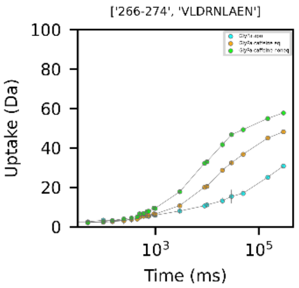

60

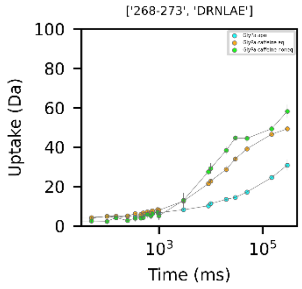

61

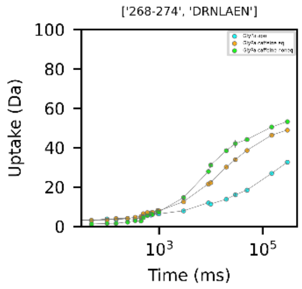

62

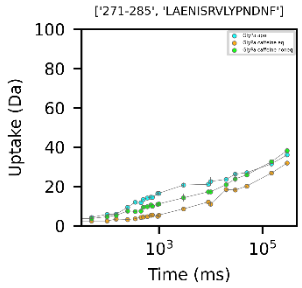

63

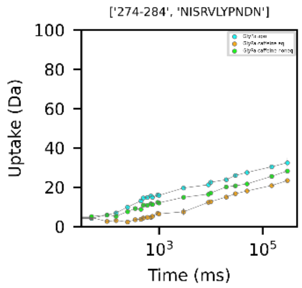

64

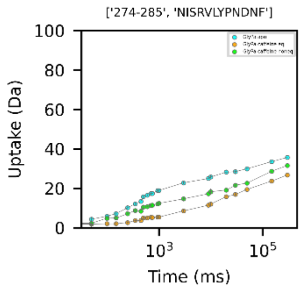

65

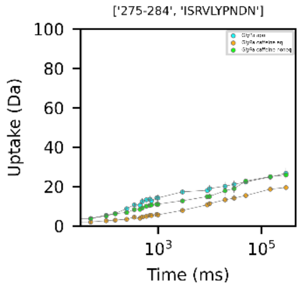

66

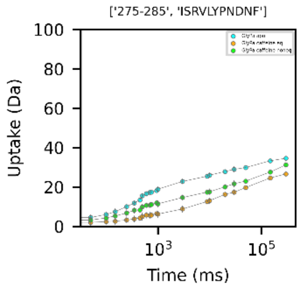

67

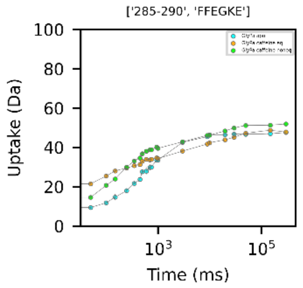

68

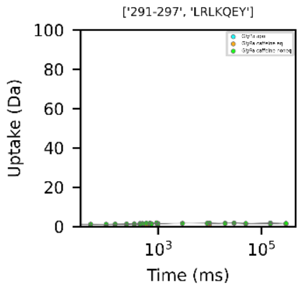

69

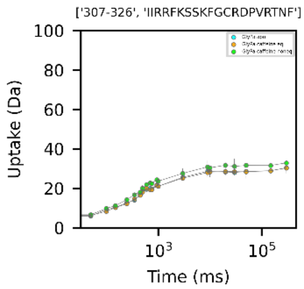

70

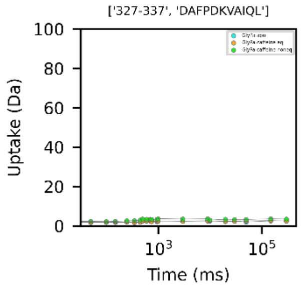

71

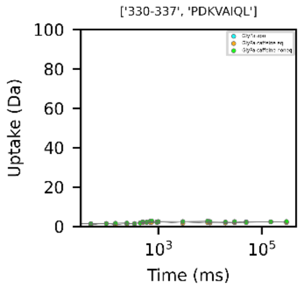

72

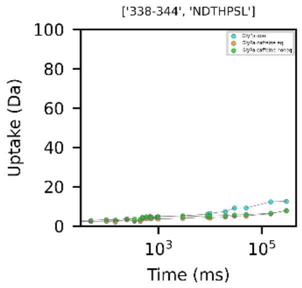

73

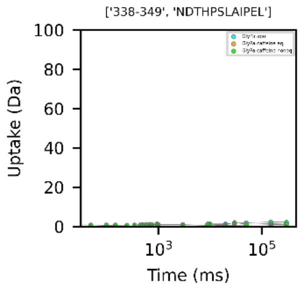

74

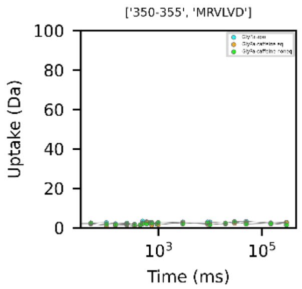

75

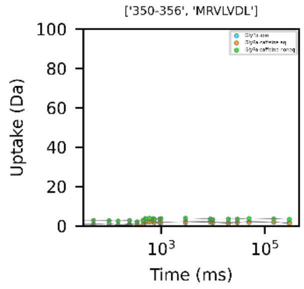

76

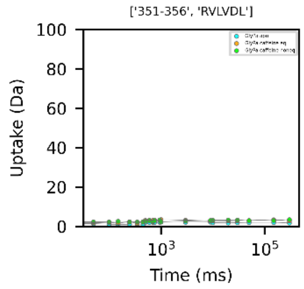

77

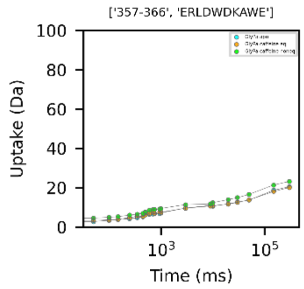

78

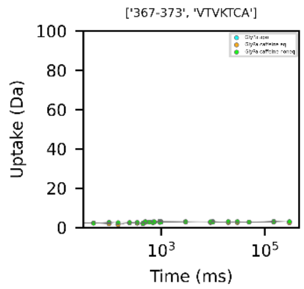

79

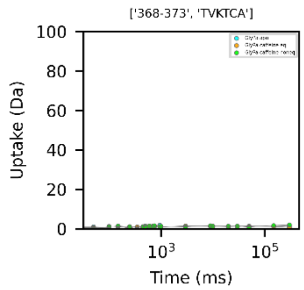

80

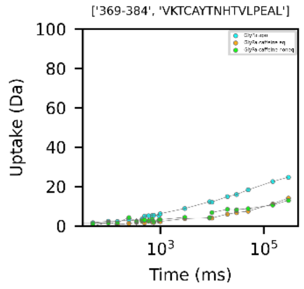

81

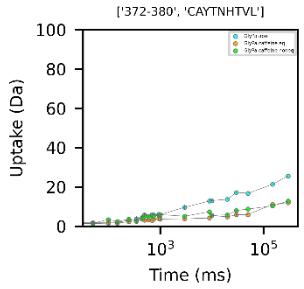

82

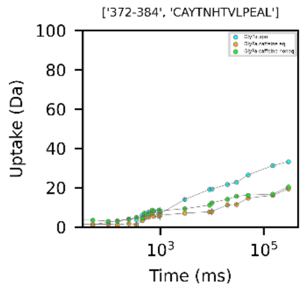

83

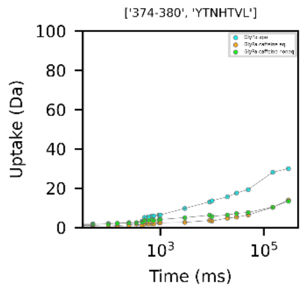

84

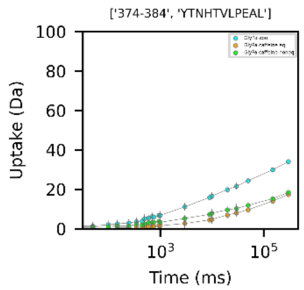

85

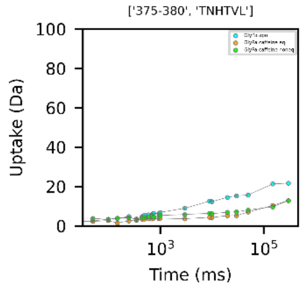

86

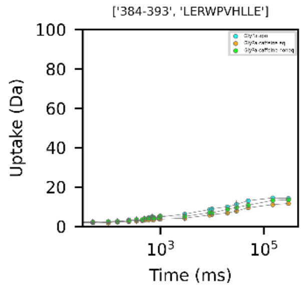

87

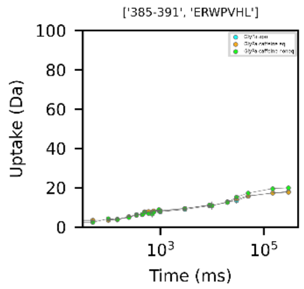

88

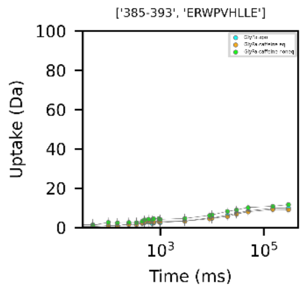

89

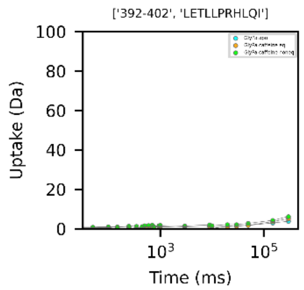

90

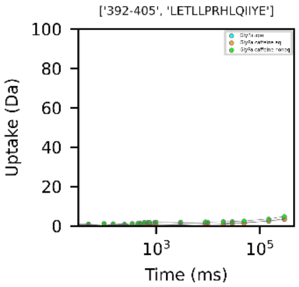

91

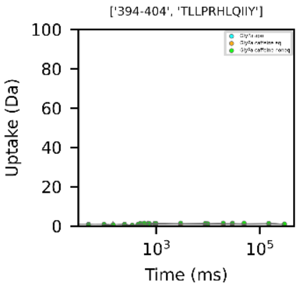

92

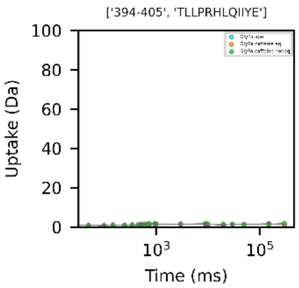

93

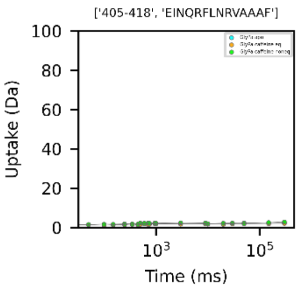

94

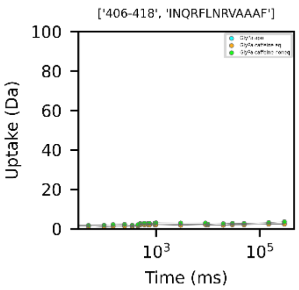

95

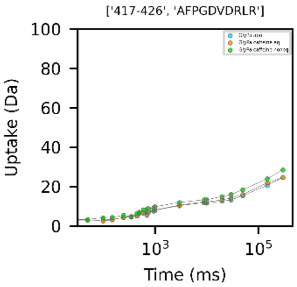

96

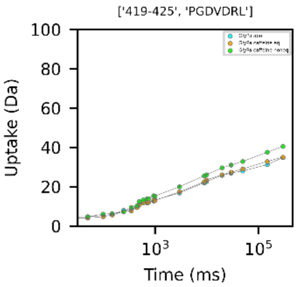

97

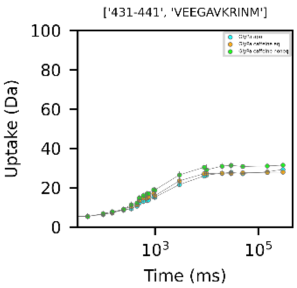

98

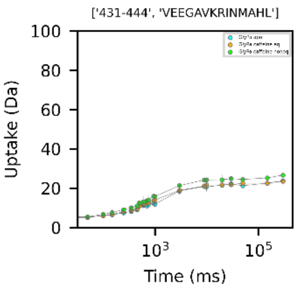

99

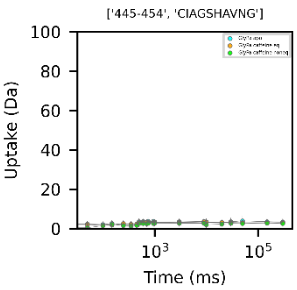

100

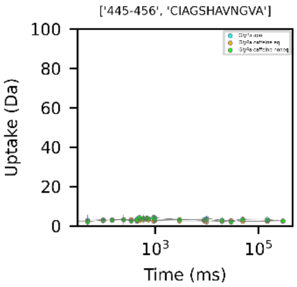

101

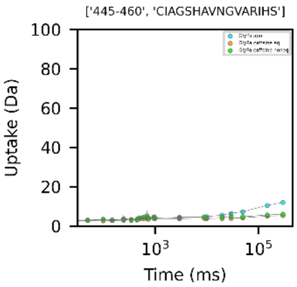

102

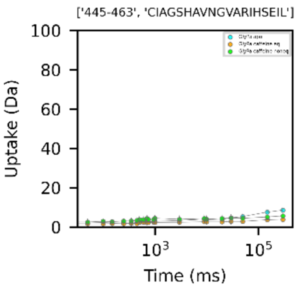

103

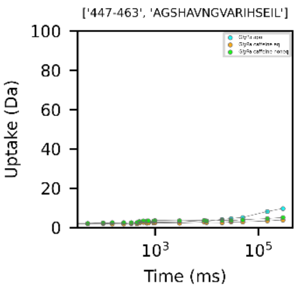

104

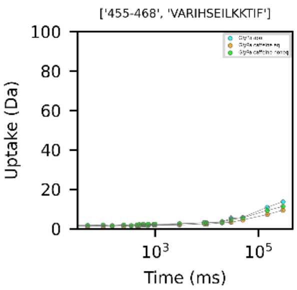

105

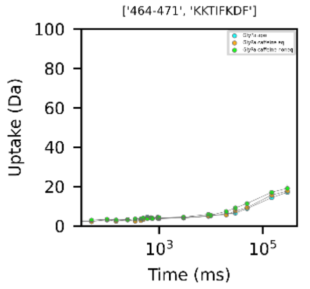

106

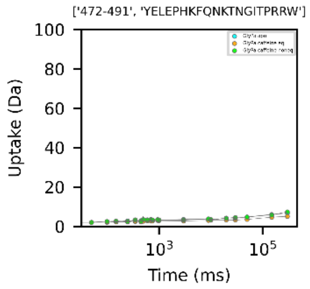

107

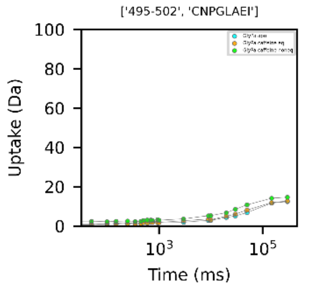

108

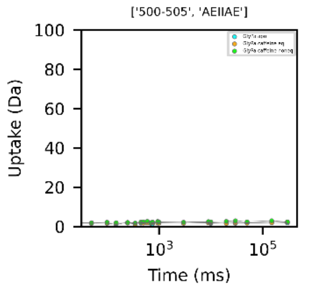

109

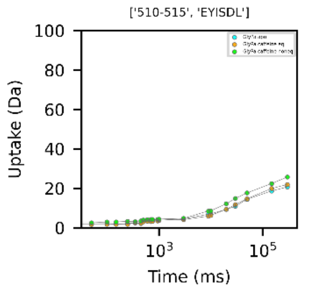

110

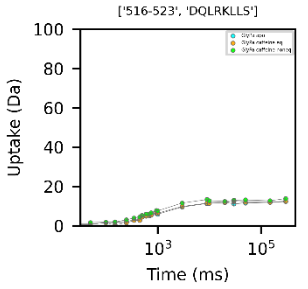

111

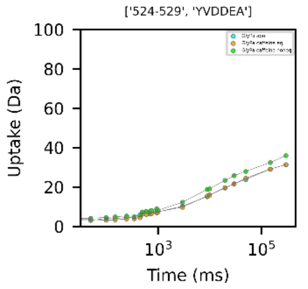

112

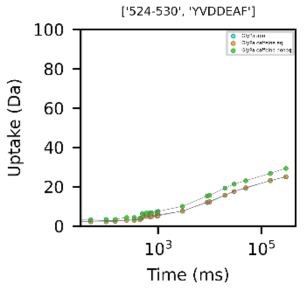

113

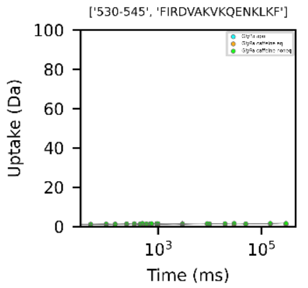

114

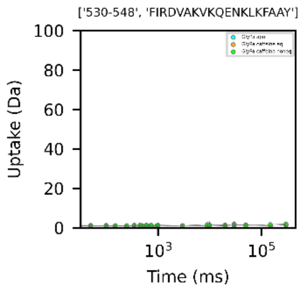

115

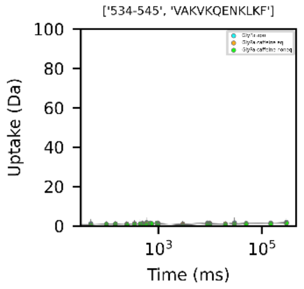

116

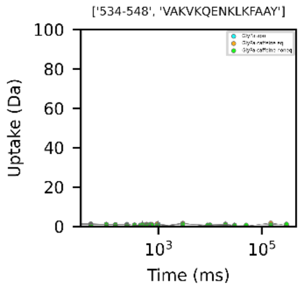

117

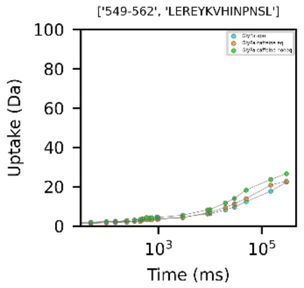

118

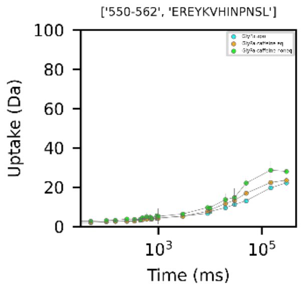

119

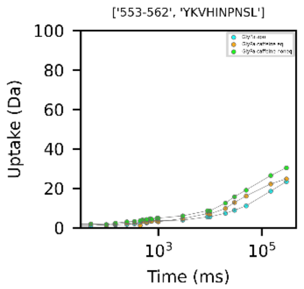

120

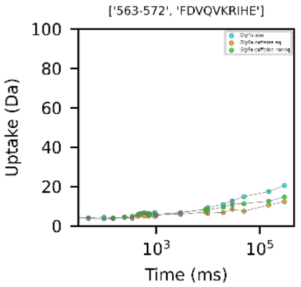

121

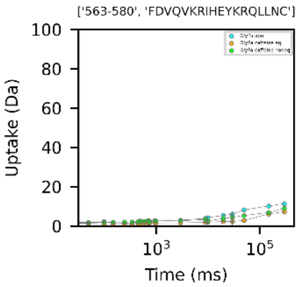

122

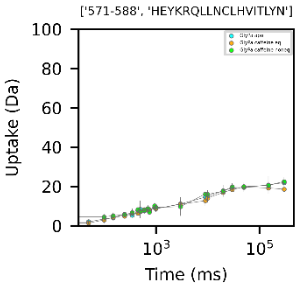

123

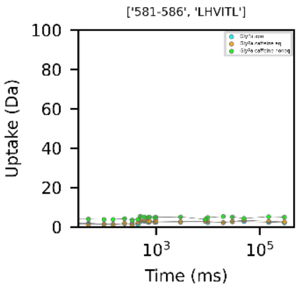

124

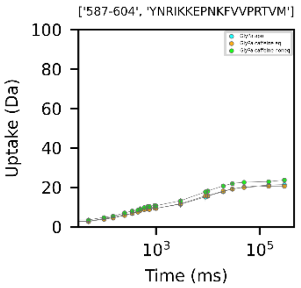

125

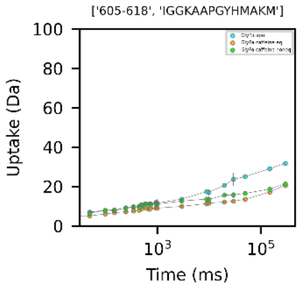

126

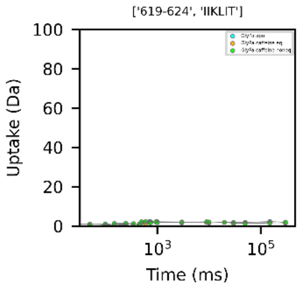

127

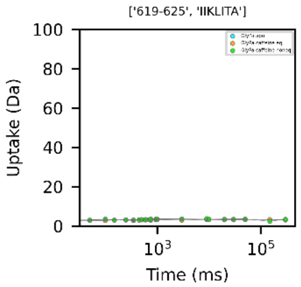

128

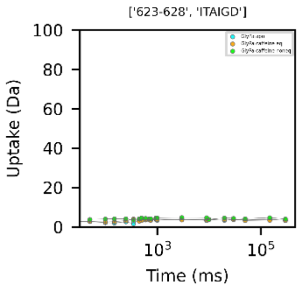

129

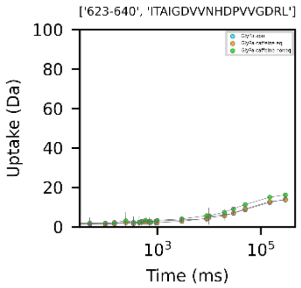

130

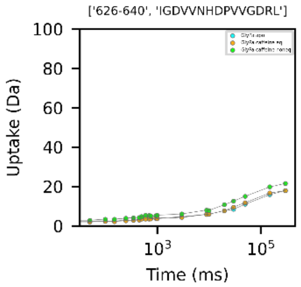

131

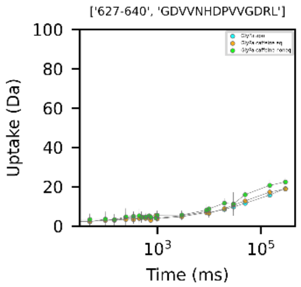

132

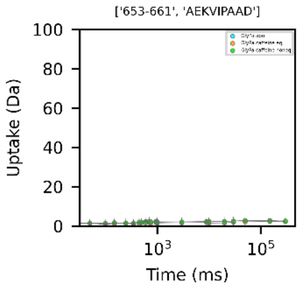

133

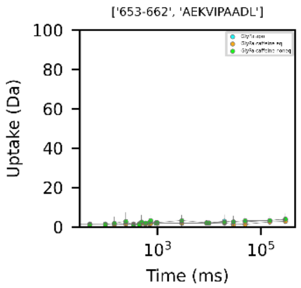

134

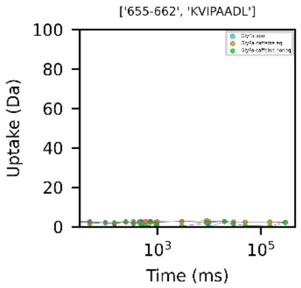

135

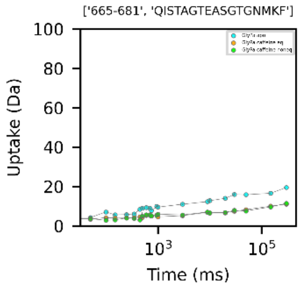

136

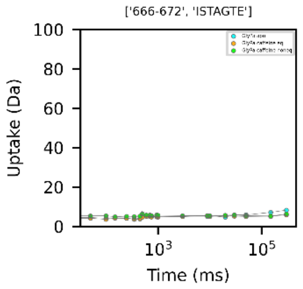

137

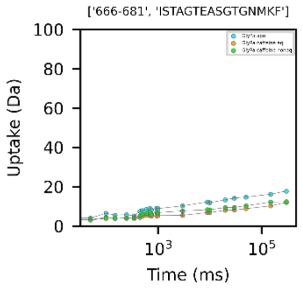

138

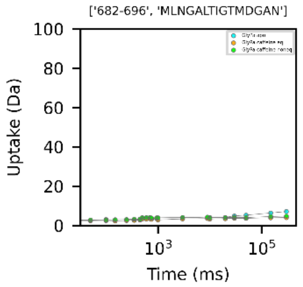

139

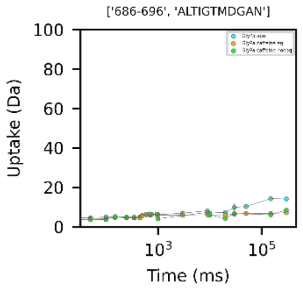

140

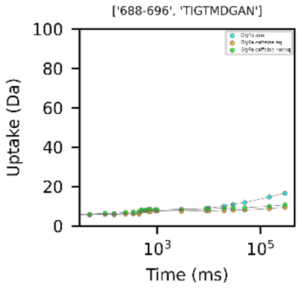

141

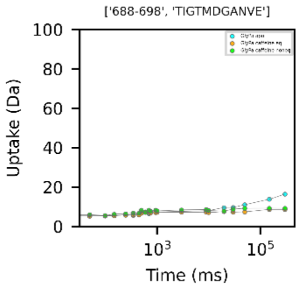

142

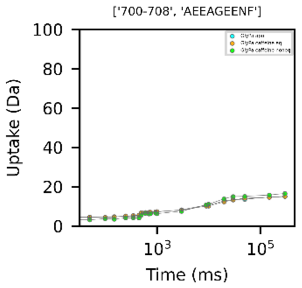

143

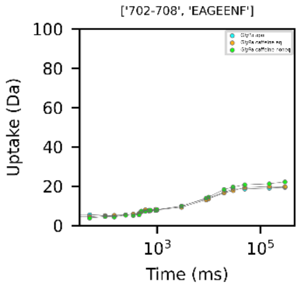

144

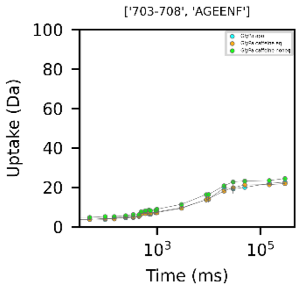

145

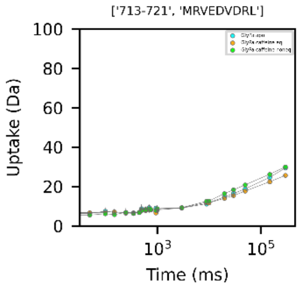

146

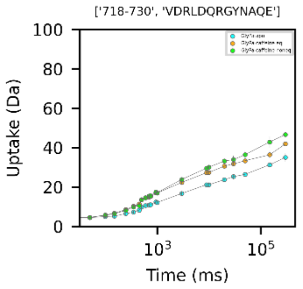

147

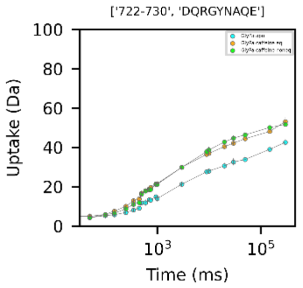

148

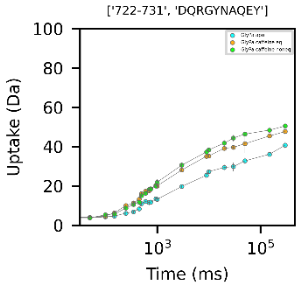

149

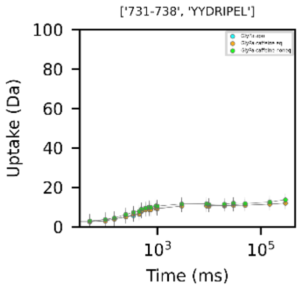

150

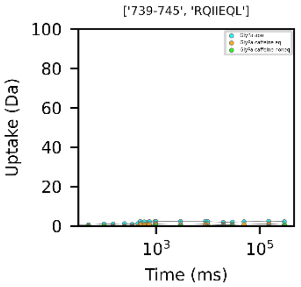

151

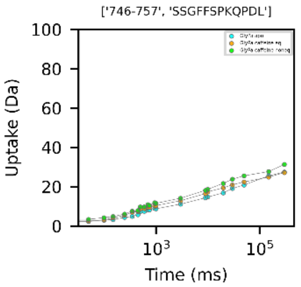

152

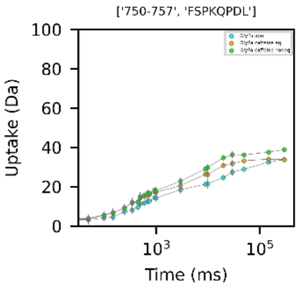

153

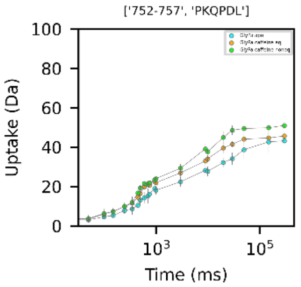

154

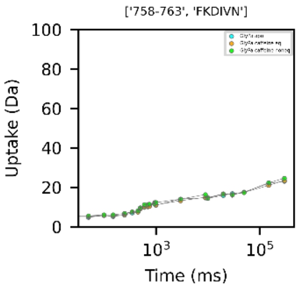

155

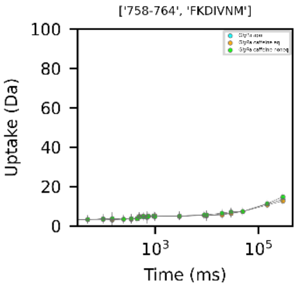

156

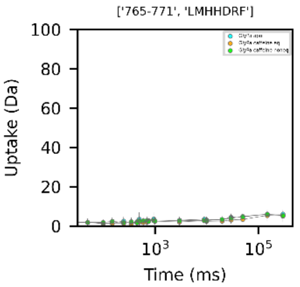

157

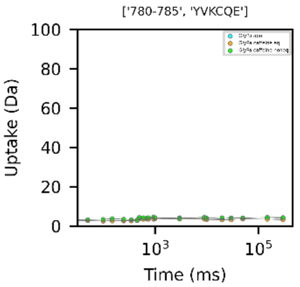

158

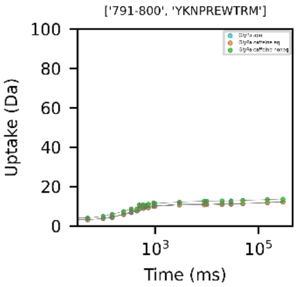

159

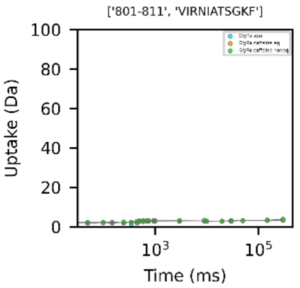

160

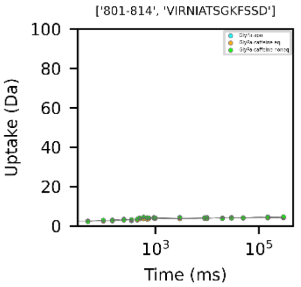

161

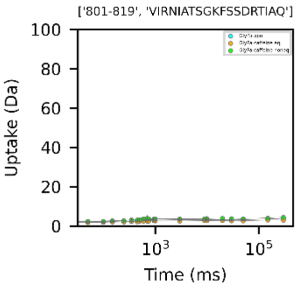

162

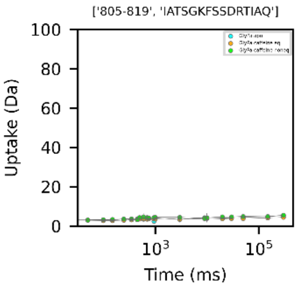

163

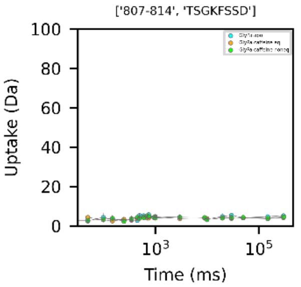

164

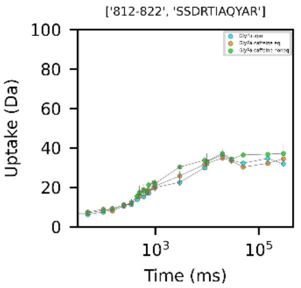

165

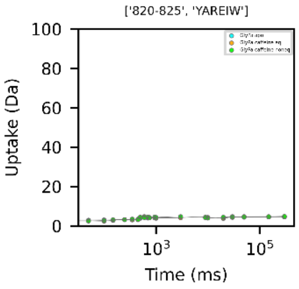

166

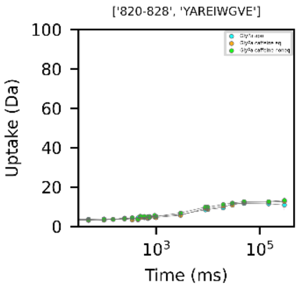

167

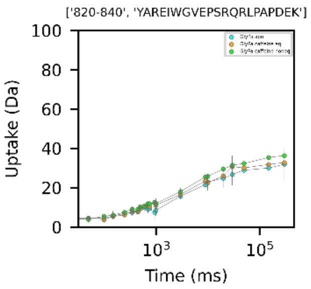

168

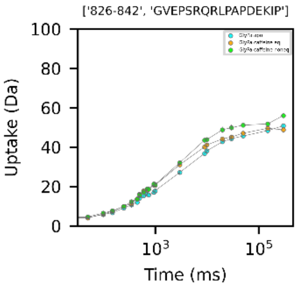

169

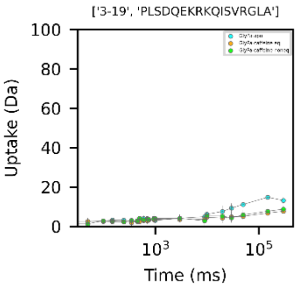

170

Table S2. Local hydrogen exchange rate (k_{obs}) of GlyPb peptides. Exchange rates were calculated by fitting a stretched exponential model to HDX-MS data with 31 time points for 219 peptide segments, for three conditions: (i) inactive GlyPb (apo), (ii) fully activated GlyPb (denoted eq) equilibrated for one hour with 25 mM AMP, 25 mM AS and (iii) GlyPb activated at non-equilibrium with 25 mM AMP, 25 mM AS (denoted by non-eq).

Sequence	Start	End	q	k_{obs} apo (s^{-1})	k_{obs} eq (s^{-1})	k_{obs} non-eq (s^{-1})
VENVTE	21	26	5	3.60E-02	5.95E-02	7.47E-02
NVTELKKNFNRHLHF	23	37	14	1.60E-04	2.53E-04	3.84E-05
LKKNFNRHLHFTL	27	39	12	1.01E-06	7.87E-07	1.03E-06
TLVKDRNVAT	38	47	9	6.31E-05	6.46E-06	1.15E-05
TLVKDRNVATPRD	38	50	11	2.44E-04	1.32E-04	3.89E-04
TLVKDRNVATPRDYY	38	52	13	1.19E-04	1.70E-05	1.41E-04
VKDRNVATPRD	40	50	9	4.73E-04	1.14E-04	7.35E-04
VKDRNVATPRDYY	40	52	11	1.71E-04	7.90E-05	3.41E-05
DYYFALAHTVR	50	60	10	2.39E-07	4.70E-06	2.40E-07
YYFALA	51	56	5	8.03E-07	6.28E-07	7.85E-07
FALAHTVRDHLVGRW	53	67	14	2.57E-05	1.41E-06	4.88E-06
ALAHTVRDHLVGRW	54	67	13	3.20E-06	1.68E-06	5.57E-05
IRTQQHY	68	74	6	3.01E-06	3.75E-07	3.83E-07
IRTQQHYYEKDPKRIYY	68	84	15	3.92E-04	3.77E-05	3.93E-05
YEKDPKRIYY	75	84	8	1.92E-04	1.50E-04	5.36E-04
PKRIYY	79	84	4	1.25E-03	1.99E-03	9.62E-04
EFYMGRTLQNT	88	98	10	2.05E-04	1.83E-04	2.01E-04
EFYMGRTLQNTM	88	99	11	3.66E-04	3.32E-04	3.95E-04
FYMGRTLQNTM	89	99	10	9.23E-05	8.31E-05	1.57E-04
YMGRTLQNT	90	98	8	7.73E-05	3.77E-06	9.11E-06
YMGRTLQNTM	90	99	9	4.13E-05	2.29E-06	8.22E-06
ALENACD	103	109	6	3.04E-03	2.17E-03	6.53E-04
ALENACDE	103	110	7	8.64E-04	2.04E-03	3.21E-03
EATYQLGLD	110	118	8	5.49E-04	5.78E-04	4.20E-04
ATYQLGL	111	117	6	4.13E-05	3.54E-06	5.80E-05
ATYQLGLD	111	118	7	3.84E-04	4.91E-04	1.27E-03
YQLGLD	113	118	5	7.31E-04	1.49E-04	1.07E-04
EEIEEDAGLGNGGLGRL	123	139	16	3.06E-04	5.71E-05	1.34E-04
IEEDAGLGNGGLGRL	125	139	14	3.87E-04	1.08E-04	1.56E-04
IEEDAGLGNGGLGRLAA	125	141	16	3.74E-04	4.15E-05	6.02E-05
IEEDAGLGNGGLGRLAAC	125	142	17	8.63E-05	3.13E-05	4.97E-05
DAGLGNGGLGRL	128	139	11	4.26E-04	1.57E-04	4.77E-04
DAGLGNGGLGRLAA	128	141	13	2.98E-04	7.79E-05	9.48E-05
DAGLGNGGLGRLAAC	128	142	14	2.46E-04	8.08E-05	4.69E-04
LDSMAT	144	149	5	0.00E+00	0.00E+00	0.00E+00
LDSMATLGL	144	152	8	0.00E+00	0.00E+00	0.00E+00
MATLGL	147	152	5	0.00E+00	0.00E+00	0.00E+00
MATLGLAA	147	154	7	1.47E-06	1.16E-06	2.72E-06
ATLGLAA	148	154	6	1.73E-04	5.09E-05	2.31E-04
AAYGYG	153	158	5	0.00E+00	0.00E+00	0.00E+00
AAYGYGIRYEF	153	163	10	1.81E-04	0.00E+00	5.16E-05
GYGIRYEF	156	163	7	5.99E-07	2.22E-11	1.09E-04
IRYEFGIF	159	166	7	2.24E-05	3.54E-04	1.64E-04
GIFNQKICGGWQME	164	177	13	9.00E-07	7.95E-07	7.36E-07
NQKICGGWQM	167	176	9	1.69E-06	1.11E-06	1.49E-06
QMEEADD	175	181	6	8.51E-05	1.90E-04	1.91E-04
MEEADD	176	181	5	5.74E-04	1.21E-03	1.36E-03
WLRYGNPWEKARPEF	182	196	12	2.51E-06	5.08E-06	3.40E-06
WLRYGNPWEKARPEFTL	182	198	14	1.45E-06	1.14E-05	7.93E-06
WLRYGNPWEKARPEFTLPVHF	182	202	17	2.22E-11	0.00E+00	0.00E+00
LRYGNPWEKARPEFTL	183	198	13	5.01E-06	9.48E-06	8.19E-06
LRYGNPWEKARPEFTLPVHF	183	202	16	2.55E-06	4.26E-06	3.66E-06
YGRVEHTSQGAKWVDT	203	218	15	1.35E-03	1.23E-03	1.08E-03
YGRVEHTSQGAKWVDTQ	203	219	16	1.71E-03	5.90E-04	1.26E-03
QVVLAM	219	224	5	2.22E-11	9.59E-06	9.33E-06
QVVLAMPYDTPVPGYRN	219	235	13	8.37E-07	4.57E-06	8.39E-07
QVVLAMPYDTPVPGYRNNVVNT	219	240	18	1.69E-06	7.95E-07	1.60E-06
VVLAMPYDTPVPGYRNNVVNT	220	240	17	1.26E-06	5.50E-07	1.33E-06

LAMPYDTPVPGYRN	222	235	10	8.98E-07	7.64E-07	9.35E-07
LAMPYDTPVPGYRNNVVNT	222	240	15	3.11E-06	5.92E-06	2.25E-06
AMPYDTPVPGYRNNVVNT	223	240	14	9.30E-07	5.59E-07	1.35E-06
PYDTPVPGYRNNVVNT	225	240	12	1.26E-06	1.25E-06	1.39E-06
PGYRNNVVNTMRLWS	231	245	13	6.97E-07	6.78E-07	9.88E-07
WSAKAPNDF	244	252	7	1.13E-04	2.78E-05	4.03E-05
WSAKAPNDFNLKD	244	256	11	2.48E-03	2.39E-03	1.67E-03
AKAPNDF	246	252	5	2.86E-04	4.50E-04	8.71E-04
NLKDFNVGGYIQ	253	264	11	1.17E-02	1.62E-02	1.57E-02
FNVGGYIQ	257	264	7	3.09E-04	1.37E-03	1.51E-03
FNVGGYIQA	257	265	8	8.44E-06	8.71E-03	6.76E-03
YIQAVL	262	267	5	4.33E-05	1.39E-03	2.30E-03
VLDRNLAEN	266	274	8	3.72E-04	8.95E-04	1.91E-03
VLDRNLAENI	266	275	9	6.55E-04	1.19E-03	1.58E-03
VLDRNLAENIS	266	276	10	2.08E-05	8.86E-04	1.61E-03
DRNLAEN	268	274	6	4.99E-04	1.01E-03	2.52E-03
DRNLAENIS	268	276	8	5.77E-04	9.40E-04	1.88E-03
DRNLAENISRVLYPNDNF	268	285	16	9.31E-04	6.78E-04	1.79E-03
NISRVLYPNDNF	274	285	10	1.74E-03	1.63E-03	1.08E-03
ISRVLYPNDNF	275	285	9	1.41E-03	1.04E-03	1.03E-03
ISRVLYPNDNFF	275	286	10	2.79E-03	1.39E-03	1.24E-03
SRVLYPNDNF	276	285	8	3.89E-03	4.85E-03	2.59E-03
RVLYPNDNF	277	285	7	4.81E-03	3.38E-03	2.48E-03
FFEGKE	285	290	5	2.10E-03	3.40E-03	1.07E-03
LRLKQEY	291	297	6	3.26E-07	2.95E-07	2.67E-07
FVVAAT	298	303	5	0.00E+00	0.00E+00	0.00E+00
VVAATL	299	304	5	0.00E+00	0.00E+00	0.00E+00
DAFPDKVAIQL	327	337	9	6.39E-07	9.15E-07	2.38E-07
FPDKVAIQL	329	337	7	1.52E-06	4.99E-06	3.14E-07
PDKVAIQL	330	337	6	0.00E+00	0.00E+00	0.00E+00
DKVAIQL	331	337	6	2.22E-11	0.00E+00	0.00E+00
NDTHPSL	338	344	5	8.34E-07	1.44E-06	6.34E-07
NDTHPSLAIPELM	338	350	10	0.00E+00	0.00E+00	0.00E+00
MRVLVD	350	355	5	0.00E+00	0.00E+00	0.00E+00
MRVLVDL	350	356	6	0.00E+00	0.00E+00	0.00E+00
RVLVDL	351	356	5	0.00E+00	0.00E+00	0.00E+00
LERLDWDKA	356	364	8	5.35E-05	5.16E-05	4.96E-05
LERLDWDKAWE	356	366	10	7.81E-06	6.87E-06	9.89E-06
ERLDWDKA	357	364	7	4.52E-05	4.14E-05	4.30E-05
ERLDWDKAWE	357	366	9	2.93E-05	2.08E-05	1.44E-05
WEVTVKTCA	365	373	8	1.01E-04	2.79E-05	2.70E-04
TVKTCA	368	373	5	0.00E+00	3.75E-06	0.00E+00
CAYTNHTVLPEAL	372	384	11	6.91E-04	1.88E-03	1.20E-03
YTNHTVL	374	380	6	1.40E-04	1.84E-04	1.32E-04
YTNHTVLPEAL	374	384	9	2.06E-03	2.07E-03	1.97E-03
TNHTVL	375	380	5	7.42E-04	8.58E-04	8.99E-04
ERWPVHL	385	391	5	1.28E-05	1.30E-05	1.59E-05
ERWPVHLLE	385	393	7	1.10E-05	1.31E-05	5.37E-05
LETLLPRHL	392	400	7	3.36E-05	1.33E-05	3.98E-05
LETLLPRHLQI	392	402	9	4.90E-06	2.04E-06	3.15E-06
LETLLPRHLQIIY	392	404	11	6.12E-05	4.36E-05	4.46E-05
LETLLPRHLQIIYE	392	405	12	1.97E-05	0.00E+00	0.00E+00
ETLLPRHLQIIY	393	404	10	2.81E-05	1.77E-05	2.22E-11
TLLPRHLQIIY	394	404	9	3.55E-06	1.20E-06	0.00E+00
TLLPRHLQIIYE	394	405	10	0.00E+00	0.00E+00	0.00E+00
LPRHLQIIYE	396	405	8	1.27E-06	1.48E-06	1.91E-07
PRHLQIIYE	397	405	7	3.82E-06	3.61E-06	3.61E-06
IYEINQRFLNRVAAAF	403	418	15	0.00E+00	0.00E+00	0.00E+00
EINQRFLNRVAAAF	405	418	13	6.15E-07	5.07E-06	3.42E-06
EINQRFLNRVAAAFPGDVDRL	405	425	19	1.68E-06	1.88E-06	1.57E-06
INQRFLNRVAAAF	406	418	12	4.89E-06	2.87E-06	5.72E-07
INQRFLNRVAAAFPGDVDRL	406	425	18	2.03E-06	1.74E-06	1.66E-06
LNRVAAAFPGDVDRL	411	425	13	1.88E-06	3.33E-06	2.17E-06
PGDVDRL	419	425	5	5.75E-04	5.73E-04	2.99E-04

VEEGAVKRINM	431	441	10	7.80E-05	1.17E-04	8.47E-05
EEGAVKRINM	432	441	9	1.49E-05	6.76E-06	3.24E-06
CIAGSHAVNG	445	454	9	8.01E-07	8.19E-07	8.33E-07
CIAGSHAVNGVARIHS	445	460	15	8.78E-07	3.27E-06	1.30E-06
CIAGSHAVNGVARIHSE	445	461	16	6.52E-07	3.91E-06	7.66E-07
CIAGSHAVNGVARIHSEILKKTIF	445	468	23	3.56E-05	6.86E-05	3.56E-04
AGSHAVNGVARIHSE	447	461	14	6.51E-07	4.54E-06	8.96E-07
AGSHAVNGVARIHSEIL	447	463	16	6.76E-07	1.52E-06	5.80E-07
AGSHAVNGVARIHSEILKKTIF	447	468	21	5.31E-05	1.08E-04	2.53E-04
KKTIFKDF	464	471	7	4.58E-04	7.73E-04	7.09E-04
YELEPHKFQNKTNGITPRRW	472	491	17	1.13E-06	1.10E-06	4.88E-07
YELEPHKFQNKTNGITPRRWLVL	472	494	20	6.80E-07	5.53E-07	5.20E-07
LVLCNPGL	492	499	6	6.41E-04	5.01E-04	5.67E-04
CNPGLAE	495	501	5	4.63E-05	1.15E-04	6.34E-04
CNPGLAEI	495	502	6	3.03E-04	4.13E-04	4.33E-04
GLAEII	498	503	5	0.00E+00	0.00E+00	0.00E+00
EIIAERIGEE	501	510	9	6.09E-04	5.51E-04	1.55E-04
IAERIGE	503	509	6	2.90E-06	4.03E-06	1.63E-05
IAERIGEE	503	510	7	2.75E-04	4.68E-05	6.81E-04
AERIGEE	504	510	6	1.12E-04	4.19E-04	7.79E-04
YISDLDQ	511	517	6	6.95E-04	5.49E-04	1.90E-03
DQLRKLLS	516	523	7	2.49E-06	1.05E-05	2.67E-06
YVDDEA	524	529	5	3.66E-03	5.61E-03	9.17E-04
YVDDEAF	524	530	6	2.33E-03	2.41E-03	2.66E-03
FIRDVAKVKQENKLKFAAY	530	548	18	2.02E-06	1.92E-06	5.89E-06
IRDVAKVKQENKLKFAAY	531	548	17	2.83E-07	1.59E-06	4.46E-06
YLEREYKVHINPNSL	548	562	13	3.21E-04	2.95E-04	7.74E-04
LEREYKVHINPNSL	549	562	12	2.77E-04	2.67E-04	3.24E-04
LEREYKVHINPNSLF	549	563	13	1.69E-04	1.41E-04	1.87E-04
EREYKVHINPNSL	550	562	11	3.57E-04	3.31E-04	3.88E-04
YKVHINPNSL	553	562	8	1.81E-04	1.55E-04	2.20E-04
FDVQVKRIHE	563	572	9	1.89E-06	5.14E-06	3.13E-06
FDVQVKRIHEYKRQLLNC	563	580	17	1.30E-04	5.41E-05	1.25E-04
DVQVKRIHEYKRQLLNC	564	580	16	3.41E-05	2.68E-05	7.76E-05
LHVITL	581	586	5	0.00E+00	0.00E+00	0.00E+00
YNRIKKEPNKFVVPRTVM	587	604	15	1.88E-04	2.67E-04	2.49E-04
IGGKAAPGYHM	605	615	9	7.43E-05	3.85E-05	1.70E-05
IGGKAAPGYHMAKM	605	618	12	8.62E-05	4.72E-05	2.76E-05
GGKAAPGYHMAKM	606	618	11	1.15E-04	3.07E-05	3.59E-05
IIKLIT	619	624	5	0.00E+00	0.00E+00	0.00E+00
IIKLITA	619	625	6	4.10E-06	3.31E-06	1.74E-07
IIKLITAIGDVVNHDPVVGDRL	619	640	20	2.70E-04	9.62E-05	5.04E-04
ITAIGD	623	628	5	7.07E-07	7.28E-07	2.66E-07
TAIGDVVNHDPVVGDRL	624	640	15	8.13E-05	6.77E-05	2.74E-04
AIGDVVNHDPVVGDRL	625	640	14	2.66E-05	2.37E-05	7.50E-05
IGDVVNHDPVVGDRL	626	640	13	2.98E-05	2.84E-05	7.76E-05
LENYRVSL	645	652	7	2.66E-05	3.30E-06	3.25E-05
YRVSLAE	648	654	6	8.08E-05	3.71E-05	1.90E-05
AEKVIPAAD	653	661	7	2.51E-06	2.64E-06	8.61E-06
AEKVIPAADL	653	662	8	5.02E-07	9.88E-07	4.52E-07
SEQISTAGTEASGTGNMKF	663	681	18	2.14E-06	2.93E-06	2.05E-06
ISTAGTEASGTGNMKF	666	681	15	2.67E-06	4.99E-06	2.32E-06
MLNGALT	682	688	6	0.00E+00	0.00E+00	0.00E+00
MLNGALTIGTM	682	692	10	2.71E-06	2.76E-06	1.26E-06
MLNGALTIGTMDGAN	682	696	14	1.02E-06	1.07E-06	9.82E-07
MLNGALTIGTMDGANVE	682	698	16	1.12E-06	7.82E-07	1.09E-06
LTIGTMDGAN	687	696	9	1.20E-06	1.09E-06	9.60E-07
TIGTMDGAN	688	696	8	1.11E-06	7.16E-07	6.75E-07
TIGTMDGANVE	688	698	10	1.30E-06	5.98E-07	9.46E-07
GTMDGAN	690	696	6	7.44E-07	1.59E-06	9.82E-07
MAEEAGEENF	699	708	9	1.83E-06	2.56E-06	2.87E-06
AEEAGEENF	700	708	8	4.82E-05	4.40E-05	4.66E-05
EAGEENF	702	708	6	3.81E-04	1.97E-04	5.19E-04
AGEENF	703	708	5	1.47E-03	1.54E-03	2.65E-03

FIFGMRV	709	715	6	6.72E-04	6.63E-04	8.60E-04
FIFGMRVED	709	717	8	4.54E-04	5.12E-04	4.94E-04
RVEDVDRL	714	721	7	4.97E-05	6.69E-05	8.21E-05
VDRLDQRGYNAQE	718	730	12	1.01E-03	7.24E-04	7.06E-04
VDRLDQRGYNAQEY	718	731	13	1.00E-03	7.99E-04	6.70E-04
DQRGYNAQE	722	730	8	3.43E-03	2.33E-03	2.32E-03
DQRGYNAQEY	722	731	9	4.82E-03	1.63E-03	2.06E-03
YDRIPEL	732	738	5	6.36E-07	1.33E-06	1.52E-06
ELRQIIEQLSSGFFSPKQPDLFK	737	759	20	1.69E-04	2.56E-04	2.20E-04
RQIIEQL	739	745	6	2.22E-11	0.00E+00	0.00E+00
SSGFFSPKQPDL	746	757	9	3.79E-04	2.83E-04	4.20E-04
FFSPKQPDL	749	757	6	8.22E-04	6.45E-04	8.95E-04
FSPKQPDL	750	757	5	2.33E-03	1.84E-03	1.85E-03
SPKQPDL	751	757	4	3.15E-03	1.60E-03	2.85E-03
PKQPDL	752	757	3	1.69E-02	1.15E-02	8.36E-03
FKDIVNM	758	764	6	4.22E-05	3.79E-05	9.91E-05
YVKCQE	780	785	5	4.13E-06	1.36E-06	2.96E-07
YVKCQERVSA	780	789	9	0.00E+00	0.00E+00	0.00E+00
YVKCQERVSAL	780	790	10	0.00E+00	0.00E+00	0.00E+00
LYKNPRE	790	796	5	1.65E-06	1.75E-06	7.03E-07
YKNPREWTRM	791	800	8	9.95E-07	1.44E-06	1.39E-06
VIRNIATSGKF	801	811	10	9.75E-07	8.48E-07	7.37E-07
VIRNIATSGKFSSD	801	814	13	9.38E-07	9.72E-07	9.20E-07
VIRNIATSGKFSSDRTIAQ	801	819	18	1.01E-06	8.51E-07	9.47E-07
IATSGKFSSD	805	814	9	8.20E-07	6.45E-07	6.62E-07
IATSGKFSSDRTIAQ	805	819	14	8.36E-07	5.80E-07	7.78E-07
TSGKFSSD	807	814	7	6.11E-07	7.91E-07	8.81E-07
SGKFSSD	808	814	6	9.08E-07	8.97E-07	9.57E-07
SSDRTIAQ	812	819	7	9.46E-07	8.44E-07	7.85E-07
YAREIW	820	825	5	1.02E-06	9.90E-07	9.76E-07
YAREIWGVE	820	828	8	1.13E-04	2.86E-06	6.60E-05
IWGVEPSRQRLPAPDEKIP	824	842	14	1.11E-02	4.29E-03	5.05E-03
GVEPSRQRLPAPDEKIP	826	842	12	1.51E-02	1.71E-02	1.91E-02

Table S3. Local hydrogen exchange rate (k_{obs}) of GlyPa peptides. Exchange rates were calculated by fitting a stretched exponential model to HDX-MS data with 20 time points for 171 peptide segments, for three conditions: (i) inactive GlyPa (apo), (ii) fully activated GlyPa (denoted eq) equilibrated for one hour with 32 mM caffeine,(iii) GlyPa activated at non-equilibrium with 32 mM caffeine (denoted by non-eq).

Sequence	Start	End	q	k_{obs} apo (s^{-1})	k_{obs} eq (s^{-1})	k_{obs} non-eq (s^{-1})
PLSDQEKRKQISVRGLA	3	19	15	2.19E-09	1.21E-09	1.12E-09
SVRGLAGVEN	14	23	9	2.29E-09	2.33E-09	3.21E-09
LKKNFNRHLHF	27	37	10	1.02E-09	9.45E-10	1.17E-09
TLVKDRNVATPRD	38	50	11	6.28E-09	3.02E-08	5.30E-08
TLVKDRNVATPRDYY	38	52	13	4.91E-09	9.26E-09	2.53E-07
VKDRNVATPRDYY	40	52	11	1.38E-08	1.26E-08	2.40E-07
RDYYFALAHTVRD	49	61	12	3.44E-09	3.38E-09	4.60E-09
YYFALA	51	56	5	1.61E-09	1.11E-09	9.55E-10
FALAHTVRDHL	53	63	10	8.15E-10	3.63E-10	6.60E-10
FALAHTVRDHLVG	53	65	12	1.13E-09	8.23E-10	1.67E-09
FALAHTVRDHLVGRW	53	67	14	1.23E-09	2.36E-09	1.81E-09
HLVGRW	62	67	5	2.71E-09	1.70E-07	1.40E-07
IRTQQHY	68	74	6	5.42E-10	9.43E-10	7.00E-10
IRTQQHYYEKDPKRIYY	68	84	15	1.05E-09	2.06E-09	1.35E-09
YEKDPKRIYY	75	84	8	7.59E-09	8.34E-09	3.80E-09
PKRIYY	79	84	4	2.71E-09	2.03E-08	2.00E-09
EFYMGRTLQNTM	88	99	11	6.07E-07	6.15E-07	8.24E-07
EFYMGRTLQNTMVN	88	101	13	1.11E-09	3.25E-09	2.62E-09
FYMGRTLQNT	89	98	9	2.66E-09	1.23E-09	3.27E-08
YMGRTLQNTMVN	90	101	11	5.10E-09	2.26E-08	6.39E-09
ALENAC	103	108	5	1.22E-06	4.09E-06	8.75E-06
ALENACD	103	109	6	3.61E-07	5.89E-07	2.32E-06
ALENACDE	103	110	7	3.19E-07	4.37E-07	9.50E-07
EATYQLGLD	110	118	8	4.09E-08	3.58E-08	5.14E-08
ATYQLGLD	111	118	7	5.30E-09	1.19E-07	3.32E-08
ATYQLGLDM	111	119	8	4.02E-08	1.42E-07	4.67E-08
YQLGLD	113	118	5	8.62E-08	3.73E-08	2.24E-08
YQLGLDM	113	119	6	4.23E-08	6.45E-08	3.87E-08
EEIEEDAGLGNGGLGRL	123	139	16	1.12E-07	1.82E-07	5.71E-08
IEEDAGLGNGGLGRL	125	139	14	2.04E-07	1.22E-07	9.80E-08
IEEDAGLGNGGLGRLAA	125	141	16	8.96E-08	9.84E-08	1.50E-08
IEEDAGLGNGGLGRLAAC	125	142	17	5.40E-08	8.41E-08	2.86E-08
DAGLGNGGLGRL	128	139	11	3.98E-07	1.69E-07	1.03E-07
LDSMAT	144	149	5	5.25E-10	5.83E-10	6.26E-10
MATLGL	147	152	5	4.78E-10	8.72E-10	2.95E-10
MATLGLAA	147	154	7	4.31E-10	1.66E-09	5.53E-10
AAYGYG	153	158	5	4.27E-10	3.83E-10	1.57E-10
AAYGYGIRYEF	153	163	10	7.00E-10	9.23E-10	1.01E-09
YGYGIRYEF	155	163	8	4.92E-10	6.16E-10	5.54E-10
GIFNQKICGGWQ	164	175	11	3.46E-09	0.001128767	8.44E-10
NQKICGGWQM	167	176	9	1.35E-09	1.49E-09	1.46E-09
NQKICGGWQMEE	167	178	11	9.78E-10	7.05E-10	9.35E-10
QMEEADD	175	181	6	4.71E-07	5.82E-08	1.82E-07
MEEADD	176	181	5	3.70E-07	5.62E-08	2.39E-07
WLRYGNPWEKARPEF	182	196	12	8.12E-10	4.77E-10	8.79E-10
WLRYGNPWEKARPEFTL	182	198	14	8.62E-10	2.05E-09	3.62E-10
YGRVEHTSQGAKW	203	215	12	1.86E-07	1.61E-07	2.94E-07
YGRVEHTSQGAKWVDT	203	218	15	1.14E-07	6.35E-07	2.62E-07
YGRVEHTSQGAKWVDTQ	203	219	16	2.28E-07	3.72E-07	6.07E-07
QVVLAM	219	224	5	5.06E-10	8.08E-10	4.51E-10
AMPYDTPVPGYRN	223	235	9	1.09E-09	1.20E-09	3.99E-10
AMPYDTPVPGYRNNVVNT	223	240	14	1.16E-09	1.18E-09	1.58E-09
PVPGYRNNVVNT	229	240	9	7.44E-10	6.06E-10	9.80E-10
WSAKAPND	244	251	6	4.41E-09	2.21E-09	2.63E-09
WSAKAPNDF	244	252	7	7.48E-09	1.99E-09	1.50E-09
WSAKAPNDFNLKD	244	256	11	5.93E-07	3.07E-07	1.55E-06
NLKDFNVGG	253	261	8	1.65E-05	0.000776095	2.71E-05
NLKDFNVGGYIQ	253	264	11	4.76E-06	7.39E-06	3.81E-05

FNVGGYIQ	257	264	7	2.88E-06	3.73E-06	4.43E-05
FNVGGYIQA	257	265	8	1.37E-06	3.13E-06	2.70E-05
VLDRNLAEN	266	274	8	1.27E-07	6.01E-06	1.56E-05
DRNLAE	268	273	5	1.46E-08	4.01E-06	1.46E-05
DRNLAEN	268	274	6	1.48E-07	4.03E-06	1.38E-05
LAENISRVLYPNDNF	271	285	13	4.07E-08	2.79E-07	2.68E-07
NISRVLYPNDN	274	284	9	1.49E-08	1.65E-07	5.77E-09
NISRVLYPNDNF	274	285	10	5.02E-08	2.33E-07	2.92E-08
ISRVLYPNDN	275	284	8	1.94E-08	3.36E-08	1.49E-08
ISRVLYPNDNF	275	285	9	4.84E-08	2.16E-07	3.82E-08
FFEGKE	285	290	5	3.27E-07	2.46E-07	3.27E-07
LRLKQEY	291	297	6	8.17E-10	3.79E-09	7.52E-10
IIRRFKSSKFGCRDPVRTNF	307	326	18	2.72E-09	2.17E-09	9.20E-09
DAFPDKVAIQL	327	337	9	8.83E-10	5.15E-10	8.73E-10
PDKVAIQL	330	337	6	8.36E-10	7.81E-10	9.24E-10
NDTHPSL	338	344	5	1.49E-09	5.27E-10	5.14E-10
NDTHPSLAIPEL	338	349	9	9.22E-10	3.14E-10	3.24E-10
MRVLVD	350	355	5	5.08E-10	3.71E-10	9.15E-10
MRVLVDL	350	356	6	8.40E-10	8.69E-10	1.07E-09
RVLVDL	351	356	5	9.07E-10	3.54E-10	7.23E-10
ERLDWDKAWE	357	366	9	3.80E-09	2.54E-09	3.16E-09
VTVKTCA	367	373	6	1.93E-10	8.78E-10	1.06E-09
TVKTCA	368	373	5	8.75E-10	4.33E-10	7.94E-10
VKTCAYTNHTVLPEAL	369	384	14	1.98E-07	1.10E-08	2.15E-09
CAYTNHTVL	372	380	8	3.60E-08	1.69E-09	6.37E-10
CAYTNHTVLPEAL	372	384	11	7.92E-07	1.02E-07	4.90E-09
YTNHTVL	374	380	6	1.43E-07	5.25E-09	1.12E-09
YTNHTVLPEAL	374	384	9	2.89E-07	1.08E-07	2.18E-08
TNHTVL	375	380	5	5.06E-08	8.23E-10	7.18E-10
LERWPVHLLE	384	393	8	1.70E-08	1.51E-09	2.03E-09
ERWPVHL	385	391	5	1.36E-09	1.52E-09	6.05E-09
ERWPVHLLE	385	393	7	2.34E-09	1.41E-09	1.90E-09
LETLLPRHLQI	392	402	9	9.05E-10	8.30E-10	1.14E-09
LETLLPRHLQIIYE	392	405	12	6.95E-10	1.13E-09	6.60E-10
TLLPRHLQIIY	394	404	9	6.16E-10	1.64E-09	7.72E-10
TLLPRHLQIIYE	394	405	10	7.70E-10	6.96E-10	7.67E-10
EINQRFLNRVAAAF	405	418	13	7.46E-10	7.98E-10	8.59E-10
INQRFLNRVAAAF	406	418	12	8.06E-10	8.40E-10	8.21E-10
AFPGDVDRLR	417	426	8	1.52E-08	1.37E-08	2.44E-08
PGDVDRL	419	425	5	1.34E-07	2.11E-07	7.45E-07
VEEGAVKRINM	431	441	10	4.97E-08	1.63E-08	5.18E-08
VEEGAVKRINMAHL	431	444	13	6.37E-09	2.24E-09	8.31E-09
CIAGSHAVNG	445	454	9	9.99E-10	7.77E-10	8.76E-10
CIAGSHAVNGVA	445	456	11	5.42E-10	7.31E-10	5.16E-10
CIAGSHAVNGVARIHS	445	460	15	8.90E-10	1.12E-09	1.09E-09
CIAGSHAVNGVARIHSEIL	445	463	18	1.02E-09	8.68E-10	9.94E-10
AGSHAVNGVARIHSEIL	447	463	16	7.06E-10	8.74E-10	1.07E-09
VARIHSEILKKTIF	455	468	13	3.69E-09	1.64E-09	2.63E-09
KKTIFKDF	464	471	7	2.36E-09	7.72E-09	1.32E-09
YELEPHKFQNKTNGITPRRW	472	491	17	1.18E-09	1.09E-09	1.13E-09
CNPGLAEI	495	502	6	5.21E-09	1.27E-08	4.73E-09
AEIIAE	500	505	5	4.84E-10	4.68E-10	7.84E-10
EYISDL	510	515	5	3.84E-08	1.06E-07	7.34E-08
DQLRKLLS	516	523	7	1.52E-09	3.75E-08	7.51E-09
YVDDEA	524	529	5	2.26E-07	5.36E-07	5.03E-07
YVDDEAF	524	530	6	1.13E-07	9.09E-08	4.92E-07
FIRDVAKVKQENKLKF	530	545	15	6.71E-10	2.09E-09	5.95E-10
FIRDVAKVKQENKLKFAAY	530	548	18	2.08E-09	5.91E-10	6.76E-10
VAKVKQENKLKF	534	545	11	6.97E-10	6.28E-10	3.21E-10
VAKVKQENKLKFAAY	534	548	14	1.86E-10	3.42E-09	4.98E-10
LEREYKVHINPNSL	549	562	12	1.94E-08	7.03E-08	1.07E-07
EREYKVHINPNSL	550	562	11	3.08E-08	1.27E-07	1.06E-07
YKVHINPNSL	553	562	8	1.24E-07	2.30E-07	2.07E-07
FDVQVKRIHE	563	572	9	3.47E-09	6.18E-10	8.67E-10

FDVQVKRIHEYKRQLLNC	563	580	17	2.03E-09	5.10E-10	6.12E-10
HEYKRQLLNCLHVITLYN	571	588	17	1.64E-08	9.24E-07	3.03E-08
LHVITL	581	586	5	3.42E-10	7.64E-10	1.09E-09
YNRIKKEPNKFVVPRTVM	587	604	15	4.14E-08	1.09E-08	1.30E-07
IGGKAAPGYHMAKM	605	618	12	3.36E-08	1.04E-09	1.57E-09
IIKLIT	619	624	5	5.42E-10	2.88E-10	2.54E-10
IIKLITA	619	625	6	9.92E-10	1.20E-09	1.29E-09
ITAIGD	623	628	5	1.11E-09	1.16E-09	1.27E-09
ITAIGDVVNHDPVVGDRL	623	640	16	6.49E-09	8.90E-09	1.44E-08
IGDVVNHDPVVGDRL	626	640	13	5.61E-09	1.10E-08	1.46E-08
GDVVNHDPVVGDRL	627	640	12	2.54E-09	2.48E-09	4.23E-08
AEKVIPAAD	653	661	7	1.02E-09	8.12E-10	8.17E-10
AEKVIPAADL	653	662	8	9.80E-10	9.23E-10	9.39E-10
KVIPAADL	655	662	6	8.95E-10	1.09E-09	3.83E-10
QISTAGTEASGTGNMKF	665	681	16	1.61E-09	2.92E-06	1.22E-09
ISTAGTE	666	672	6	6.81E-10	1.17E-09	3.16E-10
ISTAGTEASGTGNMKF	666	681	15	1.04E-09	5.45E-10	1.40E-09
MLNGALTIGTMDGAN	682	696	14	1.19E-09	1.00E-09	1.02E-09
ALTIGTMDGAN	686	696	10	9.95E-10	4.41E-10	8.50E-09
TIGTMDGAN	688	696	8	9.05E-10	4.03E-10	1.55E-09
TIGTMDGANVE	688	698	10	1.03E-09	1.38E-09	1.39E-09
AEEAGEENF	700	708	8	1.44E-09	1.68E-09	1.89E-08
EAGEENF	702	708	6	2.34E-08	4.27E-09	9.67E-08
AGEENF	703	708	5	2.05E-08	1.43E-07	1.51E-07
MRVEDVDRL	713	721	8	2.64E-08	1.33E-08	3.63E-08
VDRLDQRGYNAQE	718	730	12	2.37E-07	9.72E-07	1.64E-06
DQRGYNAQE	722	730	8	6.32E-07	2.60E-06	2.73E-06
DQRGYNAQEY	722	731	9	6.69E-07	3.04E-06	6.89E-06
YYDRIPEL	731	738	6	6.99E-10	6.37E-10	9.23E-10
RQIIEQL	739	745	6	8.27E-10	3.80E-10	2.22E-14
SSGFFSPKQPDL	746	757	9	6.20E-08	2.74E-08	5.88E-08
FSPKQPDL	750	757	5	1.53E-07	7.32E-07	4.21E-07
PKQPDL	752	757	3	2.84E-06	2.35E-06	5.88E-06
FKDIVN	758	763	5	1.27E-08	2.87E-09	2.47E-09
FKDIVNM	758	764	6	9.48E-10	7.77E-10	8.89E-10
LMHHDRF	765	771	6	1.11E-09	1.05E-09	1.14E-09
YVKCQE	780	785	5	5.99E-10	8.52E-10	9.90E-10
YKNPREWTRM	791	800	8	1.20E-09	1.11E-09	1.37E-09
VIRNIATSGKF	801	811	10	8.93E-10	1.05E-09	9.28E-10
VIRNIATSGKFSSD	801	814	13	1.09E-09	9.93E-10	8.93E-10
VIRNIATSGKFSSDRTIAQ	801	819	18	9.23E-10	9.89E-10	5.91E-10
IATSGKFSSDRTIAQ	805	819	14	1.07E-09	5.76E-10	1.02E-09
TSGKFSSD	807	814	7	1.21E-09	6.58E-10	9.64E-10
SSDRTIAQYAR	812	822	10	1.33E-06	2.39E-06	1.75E-06
YAREIW	820	825	5	1.08E-09	1.08E-09	1.07E-09
YAREIWGVE	820	828	8	1.46E-09	2.42E-09	2.51E-09
YAREIWGVEPSRQRLPAPDEK	820	840	17	6.18E-07	7.45E-07	3.15E-06
GVEPSRQRLPAPDEKIP	826	842	12	3.76E-06	3.37E-06	1.89E-05
PSRQRLPAPDEKIP	829	842	9	4.95E-06	8.63E-06	1.18E-05

References

(1) Hageman, T. S.; Weis, D. D. Reliable Identification of Significant Differences in Differential Hydrogen Exchange-Mass Spectrometry Measurements Using a Hybrid Significance Testing Approach. Anal Chem. 2019, 91 (13), 8008-8016. DOI: 10.1021/acs.analchem.9b01325

(2) Seetaloo, N.; Kish, M.; Phillips, J. J. HDfleX: Software for Flexible High Structural Resolution of Hydrogen/Deuterium-Exchange Mass Spectrometry Data. Anal Chem. 2022, 94 (11), 4557-4564. DOI: 10.1021/acs.analchem.1c05339

(3) Chetty, P. S.; Mayne, L.; Lund-Katz, S.; Stranz, D.; Englander, S. W.; Phillips, M. C. Helical structure and stability in human apolipoprotein A-I by hydrogen exchange and mass spectrometry. Proc Natl Acad Sci U S A 2009, 106 (45), 19005-19010. DOI: 10.1073/pnas.0909708106

(4) Fiser, A.; Do, R. K. G.; Šali, A. Modeling of loops in protein structures. Protein Science. 2000, 9 (9), 1753-1773. DOI: 10.1110/ps.9.9.1753

(5) Sali, A.; Blundell, T. L. Comparative protein modelling by satisfaction of spatial restraints. J Mol Biol. 1993, 234 (3), 779-815. DOI: 10.1006/jmbi.1993.1626
